# Evidence-based consensus guidelines for the management of catatonia: Recommendations from the British Association for Psychopharmacology

**DOI:** 10.1177/02698811231158232

**Published:** 2023-04-11

**Authors:** Jonathan P Rogers, Mark A Oldham, Gregory Fricchione, Georg Northoff, Jo Ellen Wilson, Stephan C Mann, Andrew Francis, Angelika Wieck, Lee Elizabeth Wachtel, Glyn Lewis, Sandeep Grover, Dusan Hirjak, Niraj Ahuja, Michael S Zandi, Allan H Young, Kevin Fone, Simon Andrews, David Kessler, Tabish Saifee, Siobhan Gee, David S Baldwin, Anthony S David

**Affiliations:** 1 Division of Psychiatry, University College London, London, UK; 2 South London and Maudsley NHS Foundation Trust, London, UK; 3 Department of Psychiatry, University of Rochester Medical Center, Rochester, NY, USA; 4 Department of Psychiatry, Massachusetts General Hospital, Boston, MA, USA; 5 Harvard Medical School, Boston, MA, USA; 6 Mind, Brain Imaging and Neuroethics Research Unit, The Royal’s Institute of Mental Health Research, University of Ottawa, Ottawa, ON, Canada; 7 Veterans Affairs, Geriatric Research, Education and Clinical Center, Tennessee Valley Healthcare System, Nashville, TN, USA; 8 Department of Psychiatry and Behavioral Sciences, Vanderbilt University Medical Center, Nashville, TN, USA; 9 Private Practice, Harleysville, PA, USA; 10 Penn State Medical School, Hershey Medical Center, PA, USA; 11 Greater Manchester Mental Health NHS Foundation Trust, Manchester, UK; 12 Institute of Population Health, University of Manchester, Manchester, UK; 13 Kennedy Krieger Institute, Baltimore, Maryland, USA; 14 Department of Psychiatry, Johns Hopkins School of Medicine, Baltimore, Maryland, USA; 15 Department of Psychiatry, Postgraduate Institute of Medical Education and Research, Chandigarh, CH, India; 16 Department of Psychiatry and Psychotherapy, Central Institute of Mental Health, Medical Faculty Mannheim, University of Heidelberg, Mannheim, Germany; 17 Regional Affective Disorders Service, Cumbria, Northumberland, Tyne and Wear NHS Foundation Trust, Newcastle, UK; 18 Queen Square Institute of Neurology, University College London, London, UK; 19 National Hospital for Neurology and Neurosurgery, London, UK; 20 Department of Psychological Medicine, Institute of Psychiatry, Psychology and Neuroscience, King’s College London, UK; 21 School of Life Sciences, Queen’s Medical Centre, The University of Nottingham, Nottingham, UK; 22 Patient and Retired Physician, Liverpool, UK; 23 Centre for Academic Mental Health, University of Bristol, Bristol, UK; 24 Pharmacy Department, South London and Maudsley NHS Foundation Trust, London, UK; 25 Faculty of Life Sciences and Medicine, King’s College London, London, UK; 26 Clinical Neuroscience, Clinical and Experimental Sciences, Faculty of Medicine, University of Southampton, Southampton, UK; 27 Institute of Mental Health, University College London, London, UK

**Keywords:** Catatonia, catatonic schizophrenia, guideline, treatment, benzodiazepine, electroconvulsive therapy, neuroleptic malignant syndrome

## Abstract

The British Association for Psychopharmacology developed an evidence-based consensus guideline on the management of catatonia. A group of international experts from a wide range of disciplines was assembled. Evidence was gathered from existing systematic reviews and the primary literature. Recommendations were made on the basis of this evidence and were graded in terms of their strength. The guideline initially covers the diagnosis, aetiology, clinical features and descriptive epidemiology of catatonia. Clinical assessments, including history, physical examination and investigations are then considered. Treatment with benzodiazepines, electroconvulsive therapy and other pharmacological and neuromodulatory therapies is covered. Special regard is given to periodic catatonia, malignant catatonia, neuroleptic malignant syndrome and antipsychotic-induced catatonia. There is attention to the needs of particular groups, namely children and adolescents, older adults, women in the perinatal period, people with autism spectrum disorder and those with certain medical conditions. Clinical trials were uncommon, and the recommendations in this guideline are mainly informed by small observational studies, case series and case reports, which highlights the need for randomised controlled trials and prospective cohort studies in this area.

## Contents

Introduction 2

Guideline rationale 2

Guideline method 2

Strength of evidence and recommendations 3

Background 3

History 3

Definition 3

Aetiology 5

Catatonia due to a medical condition 5

Catatonia due to another psychiatric disorder 6

Clinical features 6

Descriptive epidemiology 8

Clinical assessment 8

History and physical examination 8

Rating instruments 9

Investigations 10

Challenge tests 12

Lorazepam and other benzodiazepines 12

Zolpidem 13

Other drugs 13

Differential diagnosis 13

Treatment 16

General approach 16

First-line treatment 16

Non-response 17

Underlying condition 17

Complications 17

GABA-ergic pharmacotherapies 17

Electroconvulsive therapy 18

Other therapies 19

NMDA receptor antagonists 19

Dopamine precursors, agonists and reuptake inhibitors  20

Dopamine receptor antagonists and partial agonists  20

Anticonvulsants   21

Anticholinergic agents   21

Miscellaneous treatments   21

Repetitive transcranial magnetic stimulation and transcranial direct-current stimulation as alternatives to ECT  21

Subtypes of catatonia and related conditions  21

Periodic catatonia   21

Malignant catatonia  22

Neuroleptic malignant syndrome  23

Antipsychotic-induced catatonia  25

Considerations in special groups and situations  25

Children and adolescents  25

Older adults  26

The perinatal period  26

The reproductive safety of lorazepam in the perinatal period  26

The use of ECT in the perinatal period  27

Autism spectrum disorder  28

Medical conditions  28

Considerations in kidney disease  28

Considerations in liver disease  28

Considerations in lung disease  29

Research priorities  29

Acknowledgement  30

Declaration of conflicting interests  30

Funding  30

Supplemental material  30

References  30

## Introduction

### Guideline rationale

Catatonia is a severe neuropsychiatric disorder affecting movement, speech and complex behaviour, often involving autonomic and affective disturbances. It has been associated with excess morbidity and, sometimes, mortality compared to other serious mental illnesses (Funayama et al., 2018; Niswander et al., 1963; Rogers et al., 2021). For much of the 20th century, catatonia was considered a subtype of schizophrenia, but, in recent decades, emerging evidence has shown that catatonia can occur in a range of psychiatric, neurological and general medical conditions (Abrams and Taylor, 1976; Gelenberg, 1976). This is now reflected in both the *International Classification of Diseases, Eleventh Edition* (*ICD-11*) and the *Diagnostic and Statistical Manual of Mental Disorders, Fifth Edition, Text Revision* (*DSM-5-TR*), which acknowledge the existence of catatonia in a range of conditions. However, recognition of catatonia is often poor (van der Heijden et al., 2005), and knowledge about the condition and its distinctive treatments is frequently limited among clinicians (Takács et al., 2021; Wortzel et al., 2021). There are no national UK guidelines that adequately cover the management of catatonia. The only UK guidance that mentions catatonia is the 2003 National Institute for Health and Care Excellence (NICE) Technology Appraisal (TA59) on the use of electroconvulsive therapy (ECT), which recognises catatonia as an indication for ECT, but there is no consideration of pharmacological treatment for catatonia (National Institute for Health and Care Excellence, 2003). From an international perspective, the European Association of Psychosomatic Medicine (Denysenko et al., 2015) and the US Academy of Consultation-Liaison Psychiatry (Denysenko et al., 2018) have produced guidelines for the management of the subpopulation of patients with catatonia that occurs in medically ill patients. The schizophrenia guidelines from the World Federation of Societies of Biological Psychiatry, the American Psychiatric Association (APA) and the German Association for Psychiatry, Psychotherapy and Psychosomatics briefly mention catatonia and suggest treatment with benzodiazepines, glutamate antagonists (amantadine and memantine) or ECT (American Psychiatric Association, 2021; Deutsche Gesellschaft für Psychiatrie und Psychotherapie, 2019; Hasan et al., 2012). There is a clear gap in the literature for a multidisciplinary consensus guideline that comprehensively reviews the current evidence and offers treatment recommendations.

### Guideline method

To address this need for a guideline, the British Association for Psychopharmacology (BAP) convened a group of experts with representation from general adult psychiatry, neuropsychiatry, child and adolescent psychiatry, liaison (consultation-liaison) psychiatry, perinatal psychiatry, autoimmune neurology, movement disorder neurology, pharmacy and primary care. Group members spanned the UK, USA, Canada, India and Germany, and were a mixture of disease experts and those with expertise in psychopharmacology, neuroimaging, epidemiology and clinical trials. There was patient representation on the group from its inception.

A virtual meeting was convened in June 2022, where group members presented proposals for separate sections of the guideline, which were discussed by the overall group. Following the meeting, certain group members drafted sections of the guideline, which were edited and synthesised into a first draft. This draft was then disseminated to all authors for further amendments before a second draft was made for further review.

**Figure 1.**
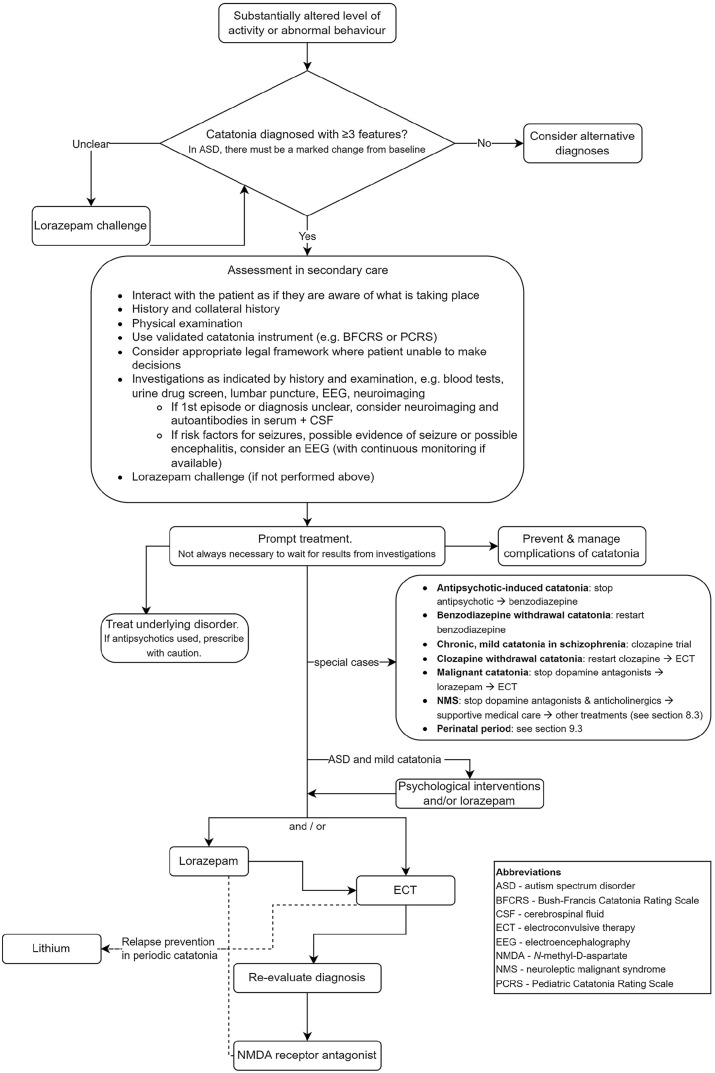
Quick reference algorithm for the management of catatonia.

The recommendations are summarised in an algorithm in Figure 1. A list of the recommendations apart from the rest of the manuscript is provided in Supplemental Material 1. Supplemental Material 2 provides a plain language summary of the guidelines for patients and carers. Example slides, which may be used for presentations of the guidelines, are available in Supplemental Material 3.

### Strength of evidence and recommendations

To assess the strength of evidence and recommendations, the guideline group adopted the schema developed by Shekelle et al. (1999). This system provides categories of evidence for the purposes of assessing causal relationships as well as a classification of the strength of recommendations. To grade the strength of evidence for non-causal relationships, we used the classification employed for the British Association for Pharmacology guidelines for the pharmacological treatment of schizophrenia, as shown in Table 1 (Barnes et al., 2020).

**Table 1.** Categories for strength of evidence and recommendations (Barnes et al., 2020; Shekelle et al., 1999).

Categories of evidence for causal relationships and treatment
Ia: Evidence from meta-analysis of RCTs
Ib: Evidence from at least one RCT
IIa: Evidence from at least one controlled study without randomisation
IIb: Evidence from at least one other type of quasi-experimental study
III: Evidence from non-experimental descriptive studies, such as comparative studies, correlation studies and case–control studies
IV: Evidence from expert committee reports or opinions and/or clinical experience of respected authorities
Categories of evidence for non-causal relationships
I: Evidence from large representative population samples
IIa: Evidence from small, well-designed, but not necessarily representative samples
IIb: Evidence from pharmacovigilance studies
III: Evidence from non-representative surveys, case reports
IV: Evidence from expert committee reports or opinions and/or clinical experience of respected authorities
Strength of recommendations
A: Directly based on category I evidence
B: Directly based on category II evidence or extrapolated recommendation from category I evidence
C: Directly based on category III evidence or extrapolated recommendation from category I or II evidence
D: Directly based on category IV evidence or extrapolated recommendation from category I, II or III evidence
S: Derived from a consensus view in the absence of systematic evidence

RCT: randomised controlled trial.

## Background

### History

Descriptions of what was likely catatonia date back to antiquity (Berrios, 1981; Jeste et al., 1985). However, major interest in motor manifestations of psychiatric disorders began only in the mid-19th century. At that time, Griesinger drew a distinction between abnormal movements that were the product of agency and those that were unconscious processes (Berrios and Marková, 2018). The term ‘catatonia’ was coined by Karl Ludwig Kahlbaum in 1874, who described an early phase of alternation between excitement and stupor, followed by a phase of qualitatively abnormal movements (Kahlbaum, 1874; Kendler, 2019), though other 19th-century authors had described similar phenomena (Hirjak et al., 2022).

By the end of the 19th century, Kraepelin’s diagnostic classifications of psychiatric disorders incorporated catatonia into an enlarged concept of dementia praecox where motor signs were the result of psychological processes (Foucher et al., 2022; Shorter and Fink, 2018), and therefore catatonia was subsumed under the diagnosis of schizophrenia by Eugen Bleuler. This differed from Kahlbaum, who had conceived of catatonia as an independent disorder with motor, behavioural and affective signs as primary manifestations of the disorder (Foucher et al., 2022; Hirjak et al., 2022). Moreover, Kahlbaum emphasised the strong occurrence of affective symptoms in combination with motor and behavioural abnormalities (Hirjak et al., 2020, 2022; Hirjak et al., 2021a; Northoff et al., 2021).

Catatonia as a subtype of schizophrenia went on to be the conceptual model used by earlier editions of the *ICD* and *DSM*. However, two papers published in 1976 challenged this assumption, arguing that catatonia appears in a range of psychiatric and medical disorders, not exclusively (or even mainly) in schizophrenia (Abrams and Taylor, 1976; Gelenberg, 1976). The current major diagnostic manuals (*ICD-11* and *DSM-5-TR*) have since endorsed a broader concept of catatonia and permit diagnosis in the context of other mental and physical disorders, as well as providing an ‘unspecified’ category.

### Definition

Unlike many psychiatric disorders, where there is an emphasis on symptoms, the clinical features of catatonia largely consist of observed or elicited signs. More than 50 such signs have been identified (Sienaert et al., 2011). These signs cover focal motor activity (e.g. catalepsy, posturing, mannerisms, stereotypies, grimacing and echopraxia), generalised motor activity (stupor and agitation), speech (mutism, verbigeration and echolalia), affect (affective blunting, anxiety and ambivalence), complex behaviour (negativism, reduced oral intake and withdrawal) and autonomic activity (tachycardia and hypertension). They concern failures in initiation of activity (stupor, mutism and reduced oral intake) and in cessation of activity (perseveration, catalepsy and posturing).

With such a wide range of clinical signs, there is a need to identify which may be specific to catatonia. Those that have little specificity (e.g. tachycardia and anxiety) are unlikely to be very useful diagnostically, although they may be helpful in gauging severity and treatment response. In terms of sensitivity, studies have failed to identify any catatonic feature that is invariably present in catatonia (Dawkins et al., 2022; Wilson et al., 2015), which is the case for many psychiatric disorders.

If there are no clinical signs that are pathognomonic of catatonia, it is reasonable to use a combination of clinical signs. The question then is how many signs should be used. Between two and four signs have been proposed as an appropriate threshold (Rasmussen et al., 2016; Zingela et al., 2022). One important study had an a priori threshold of two catatonic signs and found that there was a high response rate to a lorazepam challenge, but ultimately all included patients had at least three signs (Bush et al., 1996a, 1996b). Others propose the presence of at least one motor, one behavioural and one affective sign (Northoff et al., 1999a). Such a definition of catatonia conforms to the psychomotor concept introduced by Kahlbaum (1874), and it does not regard any of the catatonic signs as pathognomonic for catatonia (Northoff et al., 1999a).

Without a gold-standard biomarker, there can only be moderate confidence around the validity of diagnostic criteria. There is also a certain circularity to defining a syndrome based on response to benzodiazepines, then testing the same drugs as treatments. However, benzodiazepine response can perhaps be considered as a surrogate marker for some form of as yet not fully characterised pathophysiological process, although the response to benzodiazepines is not universal.

One of the more compelling pieces of evidence for a requirement of three catatonic signs derives from a cluster analysis of potential catatonic features, which distinguished patients with and without catatonia. Using this as a gold standard, the authors ascertained that a combination of at least three signs best fitted the cluster-derived catatonic syndrome (Peralta and Cuesta, 2001). A threshold of four catatonic signs is highly specific but may miss some cases and thus have poorer sensitivity (Peralta et al., 2010).

Definitions of different forms of catatonia are shown in Table 2.

**Table 2.** Key definitions.

Term	Definition
Medication-induced catatonia	Catatonia induced by administration or withdrawal of prescribed medications
Substance-induced catatonia	Catatonia induced by administration or withdrawal of psychotropic substances
Malignant (pernicious/febrile/lethal) catatonia*	A life-threatening form of catatonia that, in addition to the usual signs of catatonia, is accompanied by pronounced autonomic abnormalities. In some cases, this can lead to a life-threatening elevation in blood pressure, heart rate and body temperature with a poor outcome. Malignant catatonia occurs in only a small fraction of patients with catatonia
Catatonia in critical illness	Catatonia in patients requiring medical ICUs (e.g. intubation, ventilation or vasopressors). Although current diagnostic criteria for catatonia exclude delirium, some patients may meet syndromal criteria for both and benefit from treatment for catatonia and delirium
Periodic catatonia	A rare form of catatonia with relatively high heritability characterised by alternation between stupor and excitement
Catatonic schizophrenia	A historical subtype of schizophrenia (e.g. in *ICD-10* and *DSM-IV*) in which psychomotor disorders predominate. Other features of schizophrenia such as hallucinations, delusions and thought disorder can also be present
Organic catatonic disorder	A diagnosis listed in *ICD-10* that describes a catatonic syndrome due to a known physiological condition. Catatonic schizophrenia, delirium and stupor (e.g. dissociative) according to *ICD-10* must be excluded prior to the diagnosis. In *ICD-11*, this is listed as ‘secondary catatonia syndrome’ and in *DSM-5-TR* as ‘catatonic disorder due to another medical condition’
Psychomotor concept of catatonia	A clinical/neurobiological concept that understands catatonia as a psychomotor syndrome (in the tradition of Karl Ludwig Kahlbaum) and defines it by motor, affective and behavioural domains with their associated brain networks
Motor concept of catatonia	A clinical/neurobiological concept that understands catatonia as a primarily motor syndrome (in the tradition of Emil Kraepelin and Eugen Bleuler) and defines it mainly by motor and behavioural features and their associated brain networks

DSM: Diagnostic and Statistical Manual of Mental Disorders; ICD: International Classification of Diseases; ICU: intensive care unit; LSD: lysergic acid diethylamide; MDMA: 3,4-methylenedioxymethamphetamine.

* ‘Malignant catatonia’ is now the preferred term.

*Recommendation on the definition of catatonia*

- Catatonia should be diagnosed based on the presence of three or more catatonic signs, as in *DSM-5-TR* or *ICD-11*. (B)

### Aetiology

Catatonic signs are not uncommon and can occur in many psychiatric and medical disorders. The lingering nosological legacy of catatonic schizophrenia, whereby catatonia necessarily implied schizophrenia, has been laid to rest by *ICD-11* and *DSM-5-TR*, where catatonia can now be diagnosed in the context of many different conditions ( American Psychiatric Association, 2013; World Health Organization, 2018 ). The terms ‘organic’ or ‘secondary’ catatonia have been used in the past to signify underlying medical or neurological aetiological conditions (Ahuja, 2000). However, the distinction between ‘organic’ and ‘functional’ is perhaps best avoided due to their differing connotations in disparate clinical settings.

Our consideration of the medical and psychiatric conditions underlying catatonia is largely based on clinical judgements in the published literature about what is likely to have led to catatonia, rather than on robust epidemiological associations. Often there is a close temporal relationship and sometimes a concomitant response to treatment. However, the literature largely rests on heterogeneous case reports and series, sometimes lacking standardised assessment. Many reports do not fulfil the Bradford Hill criteria for causation (Hill, 1965). Moreover, as prolonged or severe catatonia can, in turn, result in medical complications, it can be difficult to elucidate the cause-and-effect dilemma in some cases. However, it is hard to design studies to test for aetiological links, as there is under-detection and a lack of comprehensive investigations in many cases with catatonia. This may lead to a publication bias at both ends, with many cases going under-reported but the more dramatic ones finding favour for publication.

#### Catatonia due to a medical condition

There is evidence to suggest that in about 20% of patients with catatonia in unselected populations and more than 50% of patients with catatonia in acute medical and surgical settings there is an associated medical disorder that may be contributing to their presentation; this percentage rises to almost 80% in older patients (Oldham, 2018). These figures exclude catatonic signs seen in neuroleptic malignant syndrome (NMS). There are several clinical features that suggest a higher likelihood of ‘medical catatonia’, and these include comorbid delirium, clinically significant autonomic disturbances, catatonic excitement, presence of the grasp reflex, pneumonia, known history of a neurological condition and history of seizures (Oldham, 2018).

Oldham (2018) describes the common underlying medical disorders associated with catatonia in a systematic review of 11 studies, with inflammatory brain disorders contributing 28.8% out of a total of 302 patients. These disorders include encephalitis (most common) and systemic lupus erythematosus (SLE), followed by neural injury (19.2%; with vascular and degenerative conditions the most common causes of injury), toxins or medications (11.6%; such as benzodiazepine withdrawal), structural brain pathology (9.6%; such as space occupying lesions) and epilepsy (9.3%), with miscellaneous disorders and states (such as hyponatremia, postpartum, renal failure and sepsis) contributing 19.5%. Unlike delirium, where metabolic and systemic disorders predominate, 68.9% of medical disorders underlying catatonia were secondary to a central nervous system (CNS)-specific disease (Oldham, 2018).

The medical disorders underlying catatonia listed in this guideline are not a comprehensive list, as such a compilation is out of the scope of this guidance. In Table 3, we provide a selection of the most important underlying disorders.

**Table 3.** Selected important medical conditions that may underlie catatonia.

Medical conditions associated with catatonia	
CNS autoimmunity or inflammation	Medication or drug withdrawal
• Anti-NMDA receptor encephalitis	• Alcohol
• Multiple sclerosis	• Benzodiazepines
• Other causes of autoimmune encephalitis, including paraneoplastic syndromes	• Clozapine
	• Gabapentin
• SLE	• Zolpidem
CNS infection	Metabolic disorders and states
• Bacterial meningitis or encephalitis	• Diabetic ketoacidosis
• Cerebral malaria	• Glucose-6-phosphate dehydrogenase deficiency
• HIV encephalopathy	• Hepatic encephalopathy
• Prion disease	• Homocystinuria
• Subacute sclerosing panencephalitis	• Hyperammonaemia
• Syphilis	• Hypercalcaemia
• Viral meningitis or encephalitis	• Hyponatraemia
Endocrine	• Pellagra
• Addison’s disease	• Porphyria
• Cushing’s disease	• Uraemia or renal failure
• Hyperthyroidism	• Vitamin B12 deficiency or pernicious anaemia
• Hypoparathyroidism	• Wernicke’s encephalopathy
• Hypothyroidism	• Wilson’s disease
• Panhypopituitarism	Neurodegenerative
• Phaeochromocytoma	• Dementia with Lewy bodies
Focal neurological lesions	• Frontotemporal dementia
• Lesions of varying pathophysiology to the frontal lobes, temporal lobes, parietal lobes, limbic regions, diencephalon, basal ganglia and cerebellum	• Parkinson’s disease
• Space-occupying lesion	Seizure
• Traumatic brain injury	• NCSE
• Tumour	Toxins
• Vascular injury	• Bulbocapnine
Medication or drug administration or overdose	• Carbon monoxide
• Antiretroviral drugs	• Coal gas
• Azithromycin	• Fluorinated hydrocarbons
• Antipsychotics (see section ‘Antipsychotic-induced catatonia’)	• Isopropanol
• Baclofen	Miscellaneous
• Beta-lactam antibiotics	• Burns
• Cannabis and synthetic cannabinoids	• Electrocution
• Ciclosporin	• Extrapontine myelinolysis
• Corticosteroids	• Narcolepsy
• CNS stimulants	• Posterior reversible encephalopathy syndrome
• Disulfiram	• Postoperative, including post-transplant
• Fluoroquinolones	• Respiratory failure
• Inhalants	• Systemic infection or sepsis
• Ketamine	• Toxic epidermal necrolysis
• Levetiracetam	• Tuberous sclerosis
• Lithium	
• LSD	
**• Methoxetamine**	
• Opioids	
• Phencyclidine	
• Tacrolimus	

Source: Ahuja (2000), Carroll and Goforth (2004), Denysenko et al. (2018), Fink and Taylor (2003), Oldham (2018), Rogers et al. (2019), Tatreau et al. (2018), Yeoh et al. (2022).

CNS: central nervous system; HIV: human immunodeficiency virus; LSD: lysergic acid diethylamide; NCSE: Non-convulsive status epilepticus; NMDA: *N*-methyl-D-aspartate; SLE: Systemic lupus erythematosus.

In terms of focal neurological lesions in catatonia, there are case reports of catatonia associated with lesions to the frontal, parietal and temporal lobes, basal ganglia, diencephalon and cerebellum and lesions around the third ventricle. However, larger studies have found that most of the structural neuroimaging abnormalities in catatonia consist of generalised atrophy or non-specific white matter abnormalities (Jeyaventhan et al., 2022; Magnat et al., 2022).

In terms of functional neuroimaging, decreased activation in the contralateral motor cortex, decreased regional cerebral blood flow (r-CBF) in right fronto-parietal cortex (Northoff et al., 1999c) and decreased density of γ-aminobutyric acid (GABA)-A receptors in the left sensorimotor cortex and right parietal cortex (Northoff et al., 1999b) have all been found.

#### Catatonia due to another psychiatric disorder

In *DSM-5-TR*, catatonic signs represent a specifier for autism spectrum disorder, mood disorders (major depressive disorder, bipolar I disorder and bipolar II disorder), psychotic disorders (schizophrenia, schizoaffective disorder, schizophreniform disorder, brief psychotic disorder and substance-induced psychotic disorder) and another medical condition. The *DSM-5-TR* also includes a category for unspecified catatonia (American Psychiatric Association, 2013). The *DSM-IV Handbook of Differential Diagnosis* (First et al., 1995) provided a helpful hierarchy of diagnosis for catatonia, with medical aetiology first, followed by antipsychotic-induced catatonia, then substance intoxication or withdrawal, and then bipolar disorder and major depression, and then other psychiatric disorders including schizophrenia. This remains a useful hierarchy for clinical use.

Among primary psychiatric disorders, observational studies have reported catatonia in association with depression, mania, schizophrenia, autism spectrum disorder, anxiety disorders and postpartum psychosis (Abrams and Taylor, 1976; Babu et al., 2013; Dutt et al., 2011; Kline et al., 2022; Krüger and Bräunig, 2000; Nahar et al., 2017; Starkstein et al., 1996; Stompe et al., 2002; Vaquerizo-Serrano et al., 2021). Other psychiatric disorders with evidence from case reports or case series include obsessive-compulsive disorder and post-traumatic stress disorder (Ahmed et al., 2021; Dhossche et al., 2010b; Jaimes-Albornoz et al., 2020; Shiloh et al., 1995).

### Clinical features

Given that catatonic signs can fluctuate over time, catatonic signs should be examined both cross-sectionally and longitudinally using the diagnostic systems *ICD-11* and *DSM-5-TR* or one of the available clinical rating scales (for details, see sections ‘History and physical examination’ and ‘Rating instruments’). The characteristic motor signs include mannerisms, stereotypy, festination, athetotic movements, dyskinesias, *Gegenhalten*, posturing, catalepsy, waxy flexibility (*flexibilitas cerea*), rigidity, muscular hypotonus, sudden muscular tone alterations and akinesia. The characteristic affective features include compulsive emotions, emotional lability, impulsivity, aggression, excitement, affect-related behaviour, flat affect, affective latency, anxiety, ambivalence, staring and agitation. The cognitive-behavioural catatonic features include grimacing, verbigeration, perseveration, aprosodic speech, abnormal speech, automatic obedience, echolalia/echopraxia, *Mitgehen*/*Mitmachen*, compulsive behaviour, negativism, autism/withdrawal, mutism, stupor, loss of initiative and vegetative abnormalities. From a longitudinal perspective, catatonic signs often fluctuate and patients can show different forms of catatonia at different points in their illness.

The courses and outcomes of catatonia vary. A rare form of catatonia is ‘periodic’ catatonia (see Table 2 for overview of different forms of catatonia), characterised by a cyclic pattern of akinesia (stupor) and hyperkinesia (excitement), with intervals of remission (see section ‘Periodic catatonia’ for more details). Acute catatonic states can be rapidly relieved due to early therapy or may become a residual state. The clinical profile of catatonia observed in patients with chronic psychotic disorders appears to be different from that seen in acutely emerging mostly stuporous catatonic states (see e.g. (Ungvari et al., 2005, 2010)).

### Descriptive epidemiology

Many estimates of catatonia prevalence in various populations of patients seen in mental health services are available. Solmi et al. (2018) provided a synthesis of these results and the headline figure is that about 9% (95% confidence interval (CI): 6.9–11.7%) of mental health patients have features of catatonia. However, there are some important considerations to keep in mind. First, there is considerable variation across the studies that is not explained by sampling variation alone. For example, the larger studies reported much lower prevalence. For studies where *n* was greater than 1000, the prevalence was 2.3% (95% CI: 1.3–3.9%). Some of these studies also estimated prevalence within a series of patients with schizophrenia, which might be expected to have a higher prevalence than in individuals with some other mental disorders. There did not appear to be a consistent relationship between catatonia prevalence and whether the study was conducted in high-income or low- and middle-income countries. Second, many of these studies relied upon clinical diagnoses. It is probable that catatonia is under-diagnosed clinically (van der Heijden et al., 2005) and the smaller studies were far more likely to have used a systematic means of identifying catatonia, thereby explaining higher reported prevalence.

Rogers et al. (2021) estimated an incidence of catatonia in the general population, in London, UK, finding that catatonia occurred in 10.6 (95% CI: 10.0–11.1) per 100,000 person-years, but this also relied upon the mention of catatonia in the healthcare notes. In a large recent study in US non-federal general hospitals, a discharge diagnosis with an *ICD-10* catatonia code occurred in 0.05% of hospital admissions (Luccarelli et al., 2022).

Some reports indicate a temporal decline in the diagnosis of catatonia in routinely collected data. Tanskanen (2021) described a drop in incidence of catatonic schizophrenia between the 1950s and 1970s in Finnish registry data, especially in the age group of 25–40 years. However, it is possible that this apparent decline is a result of changes in diagnostic practice rather than a true change in incidence. Van der Heijden et al. (2005) reported that the apparent decline in catatonia between 1980 and 2000 in routine diagnostic data from the Netherlands could be explained by a change in diagnostic habits. A sample of patients with more detailed clinical data illustrated a high frequency of catatonic presentations from 2001 to 2003. Rogers et al. (2021) reported an increase in incidence between 2007 and 2016. The varying interest in catatonia and changes in diagnostic practice over time make the interpretation of time trend data very difficult.

Several studies conducted in Western nations have found that catatonia was more common among individuals from ethnic minorities (Chandrasena, 1986; Dealberto, 2008; Hutchinson et al., 1999; Lee et al., 2000; Rogers et al., 2021), often by a large margin.

## Clinical assessment

### History and physical examination

Studies commonly identify at least three (Cuevas-Esteban et al., 2020; Grover et al., 2015; Krüger et al., 2003; McKenna et al., 1991; Subramaniyam et al., 2020; Ungvari et al., 2007; Wilson et al., 2015) factors or principal components of catatonia, which include hyperkinetic, hypokinetic and parakinetic (i.e. abnormal movements) phenotypes. Therefore, as a rule, catatonia should be considered as a differential diagnosis whenever a patient exhibits substantially altered levels of motor activity or abnormal behaviour, especially where it is grossly inappropriate to context.

The diagnosis of catatonia can typically be made on clinical assessment alone, even though patients with catatonia are often unable to provide a clear narrative history. Collateral sources of information should be sought to clarify potential explanations for the presenting syndrome and time course. The clinician should seek detailed information regarding the patient’s medical, neurological and psychiatric history, along with exposure to or withdrawal from medications (plasma concentration measurement may be used to ascertain concordance where available), recreational substances and blood-borne or sexually transmitted infections (Table 4). It is also important to obtain a detailed family medical, neurological and psychiatric history to identify potentially specific biological vulnerability. Physical examination is also essential (Table 5).

**Table 4.** Selected salient points in a history from a person with catatonia.

Personal and family history	Personal history
Psychiatric conditions	Psychotropic drugs
Prior catatonia or NMS	Serotonergics (including lithium)
Mood disorder	D_2_ antagonists (including antiemetics)
Psychotic disorder	Clozapine discontinuation
Neurodevelopmental disorder	Benzodiazepine or alcohol discontinuation
Prior ECT	Other medications
Neuro-medical conditions	Immune checkpoint inhibitors
Seizure disorder	Recreational drugs
Known/risk for CNS pathology	Cannabis/cannabinoids
Space-occupying lesions	Stimulants
Neurodegenerative condition	Exposures (e.g. HIV or syphilis)
Encephalitis (*esp*. autoimmune)	Sexual history
Lupus or other vasculitis	Intravenous drug use

ECT: electroconvulsive therapy; NMS: neuroleptic malignant syndrome; CNS: central nervous system; HIV: human immunodeficiency virus.

**Table 5.** Physical examination for patients with catatonia.

Volume/nutritional status
Temperature
Cardiovascular examination (especially if considering ECT)
Respiratory status (especially if on opioids, prior to benzodiazepine administration)
Neurological examination for localising signs
Evidence of deep vein thrombosis
Pressure ulcers on all potential pressure points

ECT: electroconvulsive therapy.

The overwhelming majority of patients with catatonia are assessed within secondary care (Rogers et al., 2021), which seems appropriate given the complexities of management and the risks to the patient. Every patient presenting with a first lifetime episode of catatonia should receive a thorough evaluation for potential underlying medical disorders with a focus on relevant neurological conditions (see section ‘Aetiology’) (Oldham, 2018). When a patient presents with a recurrent episode of catatonia, the assessing clinician should not presume that an adequate workup was completed previously; instead, the adequacy of prior medical evaluation should be confirmed. In addition, every time a patient presents with catatonia, a medical evaluation is important to address potential complications of catatonia (Clinebell et al., 2014), as well as for care planning.

Patients who do not participate in clinical evaluation should be assessed for the capacity to refuse evaluation and care. This is particularly important whenever catatonia is considered because several features (e.g. stupor, mutism, negativism or withdrawal) can be hard to distinguish from volitional acts. The fluctuating nature of catatonic signs can also reinforce the misinterpretation of wilful non-engagement. It is also important to keep in mind that patients with catatonia often understand what others are saying yet are unaware of their inability to respond (Northoff, 2002). As such, clinicians should speak to patients with catatonia *as though* they comprehend what is being told to them because they may; in fact, once catatonia resolves, patients may have vivid recall of what they experience while in a catatonic state.

Reliable identification of catatonia requires deliberate assessment (Table 6). Three primary means of assessment include clinical observation, elicitation and physical examination. The clinician should observe the patient before evaluation, often casually without drawing attention to the fact, while no one is interacting with them to evaluate for spontaneous expression of catatonic features. Observation should continue throughout and then after direct evaluation. Next, several features of catatonia must be elicited by environmental stimuli. For instance, demonstration of negativism requires that an instruction or prompt be given, and echophenomena require speech or behaviours to be mimicked. Assessment for catalepsy, rigidity and waxy flexibility (variously defined, see Table 8) requires physical examination. Collateral information is needed to assess the extent and duration of withdrawal, and evaluation for autonomic abnormality involves assessment of vital signs, either by chart review or by obtaining them directly.

**Table 6.** Means of assessment of catatonia.

Means of assessment	Examples		
	DSM-5-TR *and* ICD-11	ICD-11 *only*	In neither
Observation	Stupor, agitation, posturing, mannerism, stereotypy, grimacing	Impulsivity, combativeness, staring, verbigeration	
(pre, during and post exam)			
Elicitation	Mutism, negativism, echolalia, echopraxia	Ambitendency	
Physical examination	Catalepsy, waxy flexibility	Rigidity	
Collateral			Withdrawal
Review of vital signs			Autonomic abnormality

DSM: Diagnostic and Statistical Manual of Mental Disorders; ICD: International Classification of Diseases.

*Recommendations on the assessment of catatonia*

- Initial assessment and treatment of catatonia should be conducted within secondary care. (S)
- Catatonia should be considered as a differential diagnosis whenever a patient exhibits a substantially altered level of activity or abnormal behaviour, especially where it is grossly inappropriate to the context. (D)
- A collateral history should be sought wherever possible. (S)
- The history should include identification of possible medical and psychiatric disorders underlying catatonia, as well as prior response to treatment. (S)
- Physical examination should include assessment for catatonic signs, signs of medical conditions that may have led to the catatonia and signs of medical complications of catatonia. (D)
- When assessing a patient with catatonia, clinicians should interact with the person as if they are able to understand what is being said to them. (S)
- In an individual who is suspected to have catatonia, non-engagement with clinical assessment should not automatically be assumed to be wilful. Mental capacity to engage in an assessment should be assessed and, if found lacking, consideration should be given to acting in an individual’s best interests within the appropriate legal framework. (S)

### Rating instruments

Most catatonia rating instruments approach catatonia scoring in a polythetic fashion (i.e. any combination of a diverse range of clinical features can contribute towards reaching a threshold for caseness), with the Northoff Catatonia Rating Scale (NCRS) a notable exception (Table 7) (Oldham, 2022).

**Table 7.** A comparison of commonly used catatonia rating scales.

	Rogers catatonia scale* (Starkstein et al., 1996)	BFCRS (Bush et al., 1996a)	NCRS (Northoff et al., 1999a)	Bräunig Catatonia Rating Scale (Bräunig et al., 2000)	Kanner Scale (Carroll et al., 2008)
Year	1996	1996	1999	2000	2008
Sample (*n*)	Depressed with catatonia (79)	Psychosis (3)	Schizophrenia (13)	Schizophrenia (34)	None
		Mania (11)		Mania (17)	
		Depression (4)	Bipolar (15)	Depression (14)	
	Non-depressed with Parkinson disease (41)	Medical (6)	Unipolar (6)	Medical (6)	
		Other (4)			
Reference standard	*DSM-IV*	Barnes	Lohr/Wisniewski	*DSM-III-R*	None
		Lohr/Wisniewski	Rosebush		
		Rosebush			
Items	22	14 (screening^†^)	40	21	18
		23 (full scale)			
Individual item scoring	0–2	0–3	0–2	0–4	0–8
Assessment	Based on motor examination	Standard 5- to 10-min assessment	Unspecified	Semi-structured 45-min exam	Standard assessment
Item description	MRS Appendix*^1^	On scale	On scale	On scale	Only in Part 2
Threshold	8 or more	2 or more (on 14 screening items^†^)	1 or more in each domain	At least 4 scored ⩾ 2	2 or more on Part 1
All *DSM-5-TR* criteria?	Yes^‡^	Yes (all in screening instrument^†^)^‡^	Yes	Yes^‡^	Yes (8/12 in Part 1)
All *ICD-11* criteria?	Omits 7 features	Yes^‡^	Yes	Yes^‡^	Yes, but misinterprets verbigeration^§^
Notes	Uncertain generalisability*	Most widely used in clinical & research	Most comprehensive scale	Based on 45-min exam, though not described	Yet to be validated clinically
	Incomplete assessment of *ICD-11* criteria	Predicts response to lorazepam	Motor features consistent with *DSM*/*ICD*^‡^		
		Video references & Training Manual available	Assessment unspecified		

* Derived from the Modified Rogers Scale, which was validated in a schizophrenia cohort.

† The first 14 items of the BFCRS comprise the Bush-Francis Catatonia Screening Instrument (BFCSI).

‡ The definitions of posturing, catalepsy, waxy flexibility, and rigidity differ among scales (see Table 8 below). Only the NCRS defines these consistently with *DSM-5-TR* and *ICD-11*. These findings can be derived from the Bush-Francis with slight modification. Deriving these from the Bräunig would require a more substantial scoring modification.

§ Kanner incorrectly describes verbigeration as ‘gibberish’.

BFCRS: Bush-Francis Catatonia Rating Scale; DSM: Diagnostic and Statistical Manual of Mental Disorders; ICD: International Classification of Diseases; NCRS: Northoff Catatonia Rating Scale.

The Rogers Catatonia Scale (Starkstein et al., 1996) was designed to differentiate catatonic depression from non-depressed patients with Parkinson’s disease. Its exclusive focus on motoric features of catatonia means that it has uncertain generalisability to other populations. It also omits several diagnostic criteria included in the *ICD-11*. The Kanner scale (Carroll et al., 2008) also has a significant weakness in that it has yet to be validated in a clinical cohort. As such, both the Rogers and Kanner scales should be disfavoured from routine clinical use at this time.

The Bräunig Catatonia Rating Scale (Bräunig et al., 2000) has good psychometric properties and has been validated against the criteria for catatonia in *DSM-III-R*, although *DSM-III-R* is somewhat different from *DSM-5-TR* in this regard. The Bräunig scale was scored using a robust 45-min semi-structured interview, which is likely infeasible in routine clinical practice. It also has some idiosyncratic definitions of its motor signs (Table 8).

**Table 8.** Representation of items on motor tone across diagnostic manuals and major rating scales.

	DSM-5-TR	ICD-11	Northoff	Bush-Francis	Bräunig
Posturing	‘Active maintenance of a posture against gravity’				Posturing with ‘limp’ tone
Catalepsy	‘Passive induction of a posture held against gravity’				Combined as ‘waxy flexibility’
Waxy flexibility	‘Slight, even resistance to positioning’			‘Initial resistance before allowing. . .to be repositioned’	Notes: ‘“waxy” muscular resistance may be felt’
Rigidity	Not included	‘Increased muscle tone’	*ICD-11*	*ICD-11*	Posturing with ‘increased muscle tone’
			Include cogwheel	Exclude cogwheel or tremor	
		Mild to severe	Exclude tremor		

DSM: Diagnostic and Statistical Manual of Mental Disorders; ICD: International Classification of Diseases.

The two leading catatonia instruments are the Bush-Francis Catatonia Rating Scale (BFCRS) (Bush et al., 1996a) and NCRS (Northoff et al., 1999a), each with its unique strengths and weaknesses. The BFCRS is the most widely cited and clinically used scale worldwide. It has good psychometric properties and is the only scale to be validated by a lorazepam challenge (Bush et al., 1996b). Its primary limitation is its idiosyncratic definition of waxy flexibility (Table 8); however, with slight adaptation, it assesses all *DSM-5-TR* criteria in its screening instrument alone (Wilson et al., 2017), which makes for an efficient clinical evaluation. The full 23-item scale evaluates all *ICD-11* catatonia criteria. The BFCRS scale was originally validated using a standardised clinical exam against other clinical criteria (Bush et al., 1996a). It has been found to be sensitive to change in clinical status in response to treatment (Bush et al., 1996b; Girish and Gill, 2003). The exam has been further refined in a Training Manual for the BFCRS and depicted in videographic educational resources, all freely available online at https://bfcrs.urmc.edu (Oldham and Wortzel, 2022).

The NCRS has good psychometric properties and offers the most comprehensive evaluation of catatonic signs. It divides its 40 items into three categories: behaviour (15 items), motor (13 items) and affective (12 items). The NCRS assesses for all diagnostic criteria of catatonia in the *DSM-5-TR* and *ICD-11*, and its definitions of motoric findings are consistent with their definitions in these diagnostic systems as well. Among catatonia scales, the NCRS uniquely emphasises affective features. Notably, the NCRS differs from other scales by requiring the presence at least one feature in each of its three domains (i.e. motor, affective and behavioural). Although such an approach is supported by Kahlbaum’s original description and some studies on subjective reports of catatonia (Hirjak et al., 2020; Northoff et al., 1996, 2021), it is not supported by *DSM-5-TR* or *ICD-11*. With such a broad range of clinical features evaluated, the NCRS’s lack of a standardised clinical assessment is a significant limitation to its reliability.

Although most scales report high interrater reliability in published studies (see Sienaert et al. (2011) for a detailed overview), this finding does not necessarily translate to the accurate use of a scale in clinical practice. There is evidence that training using videographic resources can improve use of the BFCRS (Oldham and Wortzel, 2022; Wortzel et al., 2021, 2022). The results of a catatonia rating scale should be converted to diagnostic criteria for clinical diagnosis (Oldham, 2022).

*Recommendation on the use of rating instruments*

- When assessing for the presence of catatonia or its response to treatment, a validated instrument such as the BFCRS or the NCRS should be used. (C)
- Research on catatonia should report how individual items have been defined, including thresholds. (S)

### Investigations

The diagnosis of catatonia is made through clinical observation, interview and physical examination of the patient, as well as from collateral information from carers and review of the medical record, and in general is not established through clinical investigations (e.g. laboratory tests, brain imaging, EEG, cerebrospinal fluid (CSF) analysis, urine drug screen). Clinical investigations should be ordered based on history and clinical examination findings, taking into consideration the overall severity of illness as well as medical and psychiatric comorbid illnesses. Medical investigations are typically performed to *rule out* catatonia-like conditions or to understand the underlying aetiology of catatonia as this informs treatment and prognosis.

Although catatonia is not diagnosed through neuroimaging, given the large number of neurological conditions associated with catatonia (see Table 3), brain imaging is often requested as part of the medical evaluation of a patient with catatonia. A systematic review of structural and functional brain imaging in catatonia, which identified 137 case reports and 18 studies with multiple patients (pooled *n* = 186), found that more than 75% of cases of catatonia were associated with non-focal brain imaging abnormalities affecting several brain regions, and associated with a variety of underlying conditions, including neuroinflammatory conditions (SLE, encephalitis) (Haroche et al., 2020). The most common abnormalities in catatonia are generalised atrophy and non-specific white matter abnormalities (Haroche et al., 2020; Jeyaventhan et al., 2022; Magnat et al., 2022).

Even less is known about laboratory abnormalities present in patients experiencing catatonia. In a case–control study of 1456 patients with catatonia and 24,956 psychiatric inpatient controls, serum iron was reduced in catatonia cases (11.6 vs 14.2 μmol/L, odds ratio (OR): 0.65; 95% CI: 0.45–0.95), creatine kinase (CK) was raised (2545 vs 459 IU/L, OR: 1.53; 95% CI: 1.29–1.81), but there was no difference in C-reactive protein or white blood cell count (Rogers et al., 2021), though analysis relied on a small subset of the patients with laboratory results. *N*-methyl-D-aspartate (NMDA) receptor antibodies were significantly associated with catatonia, but there were only a small number of cases (Rogers et al., 2021). However, it should be noted that there is a strong association between anti-NMDA receptor encephalitis and catatonia, with most patients with this form of autoimmune encephalitis experiencing catatonia at some point in their illness (Espinola-Nadurille et al., 2016; Rogers et al., 2019). Other autoantibodies have also been identified in association with catatonia including anti-Hu antibodies, anti-myelin oligodendrocyte glycoprotein antibodies, antinuclear antibodies (ANA), antiphospholipid antibodies, anti-ribosomal P antibodies, anti-Ro antibodies, anti-Smith antibodies, double-stranded DNA antibodies, GABA-A receptor antibodies, GAD-65 antibodies, leucine-rich glioma-inactivated 1 antibodies, ribonucleoprotein antibodies and septin-7 antibodies (Boeke et al., 2018; Chuck et al., 2022; Endres et al., 2020; Ferrafiat et al., 2021; Fujimori et al., 2021; Harmon et al., 2022; Hinson et al., 2022; Inagaki et al., 2020; Kusztal et al., 2014; Pettingill et al., 2015; Samra et al., 2020; Witek et al., 2018). However, the prevalence and pathogenicity of these antibodies in catatonia is unclear, although it is a rapidly expanding field (Rogers et al., 2019).

In terms of neurophysiology, there is a clear case for an electroencephalogram (EEG) in the context of possible non-convulsive status epilepticus (NCSE), which can present as catatonia (Ogyu et al., 2021; Volle et al., 2021). Red flags for NCSE include subtle ictal phenomena (such as twitching of the face or extremities), comorbid neurological disease and a change in medications that affect seizure threshold (Ogyu et al., 2021; Volle et al., 2021). Another quite specific EEG finding of relevance to catatonia is the extreme delta brush, which occurs in some patients with anti-NMDA receptor encephalitis (Schmitt et al., 2012). The literature on the value of ‘encephalopathic’ findings on EEGs suggests that this is not entirely specific for a medical disorder underlying catatonia (Carroll and Boutros, 1995; Smith et al., 2012).

Any hospital work-up must weigh the potential risks and benefits of detailed investigation. Hospital investigations may contribute to anxiety (Carney et al., 2004; Lindholm et al., 1997; Puglisi et al., 2005). Given that several studies have associated catatonia with intense anxiety (Cuevas-Esteban et al., 2020; Dawkins et al., 2022; Kline et al., 2022; Northoff et al., 1996), prolonged uncertainty amid medical testing may be expected to worsen this in some patients. In addition, the costs and potential harms of investigation (e.g. radiation exposure with computed tomography (CT) imaging, or magnetic resonance imaging (MRI) scans in patients who are unable to communicate whether they have any metallic implants) must be considered.

*Recommendations on the use of investigations in catatonia*

- Investigations, such as blood tests, urine drug screen, lumbar puncture, electroencephalography and neuroimaging, should be considered based on history and examination findings, taking into account the possible diagnoses that may mimic catatonia and the possible underlying aetiology of the catatonia. (D)
- In patients experiencing a first episode of catatonia or where the diagnosis underlying catatonia is unclear, consider a CT or MRI scan of the brain. (C)
- In patients experiencing a first episode of catatonia or where the diagnosis underlying catatonia is unclear, consider assessing for the presence of antibodies to the NMDA receptor and other relevant autoantibodies in serum and CSF. (D)
- In patients with risk factors for seizures, possible evidence of a seizure or possible encephalitis, consider performing an EEG (with continuous monitoring if available). (C)

### Challenge tests

*DSM-5-TR* has included a diagnosis of unspecified catatonia to encourage early treatment while a search for an underlying disorder can continue. Challenge tests may provide support in clarifying diagnosis and appropriate treatment. This section is limited to the use of benzodiazepines and zolpidem as a diagnostic and therapeutic ‘challenge test’. These agents are discussed in greater detail in section ‘GABA-ergic pharmacotherapies’.

In 1930, Bleckwenn described the use of short-acting barbiturates to ‘render catatonic patients responsive’ (Bleckwenn, 1932; Gershon and Shorter, 2019). Lorazepam (and to a limited extent, other benzodiazepines, such as diazepam, midazolam, clonazepam and oxazepam (Abrams et al., 1978; Benazzi, 1991; Mustafa, 2017; Schmider et al., 1999)) have now replaced the use of barbiturates (such as amobarbital and sodium thiopental) as a diagnostic challenge (sometimes called the lorazepam test or the diazepam test) for confirming the diagnosis of catatonia (Kavirajan, 1999).

#### Lorazepam and other benzodiazepines

Lorazepam is an effective and clinically useful diagnostic challenge test for catatonia. It is available in oral, liquid, intramuscular (IM) and intravenous (IV) forms, and is available in a variety of clinical settings. Lorazepam is a non-selective positive allosteric modulator of GABA-A receptors. Possible therapeutic mechanisms in catatonia are discussed in section ‘GABA-ergic pharmacotherapies’.

The recommended dose for a lorazepam challenge is 1–2 mg IV (Bush et al., 1996b; Suchandra et al., 2021), IM (Bush et al., 1996b; Lin and Huang, 2013) or 2 mg oral (Ungvari et al., 1994). The response to an oral challenge is often slower than for parenteral administration and oral formulations can be harder to administer to both hyperkinetic and hypokinetic patients. A positive response to a lorazepam challenge, commonly defined as a 50% reduction in catatonic signs on a standardised scale, makes a diagnosis of catatonia more likely, but it is not 100% specific. A good response on the first day appears predictive of overall response to lorazepam (Bush et al., 1996b; Payee et al., 1999). Low serum iron has been reported as a predictor of poor response with benzodiazepines (Lee, 1998). An example protocol is provided in Table 9.

**Table 9.** Example protocol for a lorazepam challenge for catatonia.

1. Assess baseline catatonic features using a standardised instrument such as the BFCRS
2. Administer lorazepam 1–2 mg IV, or 1–2 mg IM, or 2 mg oral.
3. Re-assess catatonic features after 5 min (following IV lorazepam), 15 min (following IM lorazepam) or 30 min (following oral lorazepam). A positive response is considered a 50% reduction in score on a standardised catatonia instrument
4. If there is not a positive response, consider a further challenge (ideally parenterally), as in step 2, and re-assess

Source: Bush et al. (1996b), Sienaert et al. (2014).

BFCRS: Bush-Francis Catatonia Rating scale; IM: intramuscular; IV: intravenous.

Based on their clinical effectiveness in these conditions, benzodiazepines may also be considered as a therapeutic test in antipsychotic-induced catatonia (Fricchione et al., 1983), NMS (Kontaxakis et al., 1990) and malignant catatonia.

#### Zolpidem

Mastain et al. (1995) described a serendipitous dramatic response to oral zolpidem 10 mg in a woman with a subcortical stroke whose catatonia was largely unresponsive to lorazepam or ECT. This was followed by other positive reports (Amorim and McDade, 2016; Baptista and Choucha, 2019; Bastiampillai et al., 2016; Isomura et al., 2013; Javelot et al., 2015; Kumar and Kumar, 2020; Peglow et al., 2013; Sayadnasiri and Rezvani, 2019; Seetharam and Akerman, 2006; Thomas et al., 1997; Zaw and Bates, 1997). The response is transitory, as with benzodiazepines, and is usually observed for 3–6 h (Bastiampillai et al., 2016; Thomas et al., 2007), which is consistent with zolpidem’s short elimination half-life of 1–4 h (Hiemke et al., 2018). Catatonia has also been reported in zolpidem withdrawal (Hsieh et al., 2011).

Several reports have been published of zolpidem’s effectiveness following neurological injury due to a variety of different brain insults (Sutton and Clauss, 2017). It is not clear whether some of these cases following brain injury had undiagnosed catatonia. It appears that the positive effect of zolpidem in post-brain injury states occurs at a sub-sedative dose (Hall et al., 2010; Sutton and Clauss, 2017), and there is a suggestion of a differential response in patients with traumatic or anoxic brain injury (Zhang et al., 2021).

Zolpidem is an imidazopyridine that is a selective positive modulator of the GABA-A alpha-1 subunit and this action appears to be important for its clinical efficacy (Hall et al., 2010). It seems selective for the gamma-2 subunit of the GABA-A receptor (alpha1-beta2-gamma2 GABA-A receptor) in animal experiments (Richter et al., 2020), but the implications of this in zolpidem’s efficacy as a diagnostic challenge tool are not entirely clear.

The recommended dose of zolpidem is usually 10 mg orally for a diagnostic and/or therapeutic test (Thomas et al., 2007), but 5 mg has sometimes been used in older patients (Amorim and McDade, 2016; Isomura et al., 2013; Sayadnasiri and Rezvani, 2019). Zolpidem is available in oral formulation (and as a sublingual preparation in some countries), with no parenteral preparation available, which somewhat limits its use. A therapeutic plasma concentration of 80–150 ng/L has been suggested, with an onset of action within 10–30 min of ingestion of 10 mg zolpidem (Thomas et al., 2007).

Narayanaswamy et al. (2012) showed that mutism is not a good prognostic sign for lorazepam response, so it is interesting that zolpidem may differentially help improve impairment of verbal fluency in patients with catatonia (Sayadnasiri and Rezvani, 2019; Thomas et al., 2007).

#### Other drugs

In contrast to reports of ketamine causing catatonic signs, there is at least one report of slow IV injection of sub-anaesthetic doses of ketamine (12.5 mg) producing dramatic improvement in catatonic signs (Iserson and Durga, 2020). More studies, including randomised controlled trials (RCTs), are needed before this translates into clinical practice as a diagnostic test.

*Recommendations on the use of challenge test*

- When a diagnosis of catatonia is uncertain, a diagnostic challenge using lorazepam should be considered. (B)
- When a diagnosis of catatonia is uncertain, a diagnostic challenge using zolpidem may be considered. (C)
- In suspected or confirmed cases of catatonia, a lorazepam challenge may be used to predict future response to benzodiazepines. (B)

### Differential diagnosis

There is some overlap between the differential diagnosis of catatonia (i.e. mimics of catatonia) and the conditions that may underlie catatonia. For example, NMS is sometimes listed in both categories, probably because of diverging views as to what extent it represents a form of catatonia (see section ‘Neuroleptic malignant syndrome’). For some conditions, their status is subject to debate. In Table 10, we provide a list of some of the more important conditions that may mimic catatonia, what the similarities are and how they can be differentiated.

**Table 10.** Differential diagnosis of catatonia.

Category	Example differential diagnoses	Similarities to catatonia	Distinguishing features from catatonia
Neurological movement disorders	Stiff person syndrome	Muscle spasms and rigidity	Head retraction reflex
	Progressive encephalomyelitis with rigidity and myoclonus	Immobility in severe cases	GAD-65, glycine or DPPX antibodies usually present
		Associated with anxiety	
		Emotional stimuli can trigger muscle spasms	
		Respond to benzodiazepines	
	Causes of parkinsonism (e.g. Parkinson’s disease, drug-induced parkinsonism, cerebrovascular disease, juvenile Huntington’s disease, dementia with Lewy bodies, progressive supranuclear palsy, multiple system atrophy, corticobasal degeneration)	Poverty of movement, staring and rigidity	Patients usually interactive and cooperative
		Freezing can resemble catatonic posturing	Tremor usually present
			Insidious onset
	Dystonia	Can resemble catatonic posturing	Stupor absent
			Generally responds to anticholinergics
	Akathisia	Hyperactivity can resemble catatonic excitement	Lack of other ‘positive’ signs of catatonia (e.g. echophenomena, posturing, verbigeration)
	SS	TachycardiaPyrexiaMuscle rigidity	Triggered by serotonergic drugsMyoclonus and hyperreflexiaDiarrhoea
	NMS	See section ‘Neuroleptic malignant syndrome’	
Speech disorders	Aphasia	Transcortical sensory aphasia can feature echolalia, as patient repeats back questions rather than answers themIn severe cases, speech may be absent	Motor function intact
	Anarthria	Absence of speech	Language preserved in written form
	Selective mutism	Some variability	Communication completely comfortable in certain settings
Seizure	NCSE	Can be clinically indistinguishableMay respond to benzodiazepines	Often history of epilepsyEEG usually helpful
Locked-in syndrome	Locked-in syndrome	Near-complete absence of movement	Usually have preserved vertical gaze and blinking – generally keen to attempt to communicate using these
			MRI shows pontine lesions
			No response to benzodiazepines
Encephalopathy and disorders of consciousness	Delirium	Can coexist with catatonia	Tends to resolve with reversal of underlying medical condition (though may be delayed)
	Coma	Unresponsiveness	No resistance to eye opening
	Vegetative state	Unresponsiveness	No volitional responses and no visual trackingNo resistance to eye opening
Disorders of motivation	Abulia	Reduction/absence of *spontaneous* activity	Respond to external stimuli
	Autoactivation deficit syndrome		
	Akinetic mutism	Flat affectSeveral disorders associated with both catatonia and akinetic mutism	Sometimes a ‘telephone effect’: sudden sensory stimulus causes return of movement and speechLack of emotional disturbancePossibly no response to lorazepam
Psychiatric disorders	Mania	Can resemble catatonic excitementCan co-occur with catatonia	Irritable or expansive moodAbsence of stuporous phases
	Functional neurological disorder	Mutism and paralysis in severe cases	Usually progression from milder states of functional paralysis
	Autism spectrum disorder	See section ‘Autism spectrum disorder’	
	Intellectual disability	Stereotypies and mannerismsAbsence of speech	Chronic without sudden decompensation
**Volitional uncooperativeness**	Malingering	Mutism	Past benzodiazepine misuse
		Lack of cooperation	Simulating clinical features (e.g. pouring water to simulate incontinence)
	Factitious disorder		

Source: Arnts et al. (2020), Cuevas-Esteban et al. (2022), Denysenko et al. (2018), Harten et al. (1999), Ishizuka et al. (2022), Morrison (2006), Oldham and Lee (2015), Rasmussen et al. (2016), Taylor and Fink (2003), Wang and Rehman (2021), Wong (2010).

NCSE: non-convulsive status epilepticus; NMS: neuroleptic malignant syndrome; SS: serotonin syndrome.

As general principles, the positive features of catatonia (such as echophenomena, catalepsy and posturing) may have greater discriminatory value than some of the negative features (such as mutism and stupor). Challenge tests are useful in many situations (see section ‘Challenge tests’), but their sensitivity and specificity are imperfect; importantly, stiff person syndrome and NCSE are likely to improve with a lorazepam challenge.

Although it has been asserted that serotonin syndrome (SS) is a form of catatonia (Fink and Taylor, 2001), there is currently insufficient systematic evidence to support this claim (Katus and Frucht, 2016; Keck and Arnold, 2000; Mann et al., 2022; Rosebush and Mazurek, 2010). Furthermore, although ECT, a core intervention for catatonia, has been advocated for the treatment of SS (Fink, 1996; Fink and Taylor, 2003, 2009), recent reports suggest that it is ineffective and, in fact, may exacerbate SS (Cheng et al., 2015; Katus and Frucht, 2016; Klysner et al., 2014).

## Treatment

### General approach

The evidence base for the treatment of catatonia is not extensive. Several RCTs have been conducted, but they have usually been at high risk of bias, inadequately reported, using outdated treatments or applicable to only a small subset of patients with catatonia (Girish and Gill, 2003; McCall et al., 1992; Merlis, 1962; Miller et al., 1953; Phutane et al., 2013; Schmider et al., 1999; Ungvari, 2010; Ungvari et al., 1999; Wetzel et al., 1997; Zaman et al., 2019). One systematic review found only four studies that had more than 50 participants (Pelzer et al., 2018). Nonetheless, where there is converging evidence from multiple sources, some clinically relevant inferences can be made.

Many treatments for catatonia are unlicensed applications for licensed medicines. Relevant guidance on this issue has been produced by the General Medical Council, the Royal College of Psychiatrists in association with the BAP, and the Royal College of Paediatrics and Child Health (General Medical Council, 2022; Royal College of Paediatrics and Child Health and Neonatal & Paediatric Pharmacists Group, 2013; Royal College of Psychiatrists Psychopharmacology Committee, 2017). While this guidance recommends that prescribing should usually be within a product’s licence, it is recognised that there are situations in which prescribing off-licence is appropriate. Beyond the common standards for good prescribing, it is advised to use licensed medications in preference where appropriate, to be familiar and satisfied with evidence for safety and efficacy, to seek advice where necessary, giving sufficient information to patients, to inform patients that a medicine is being used outside its licence, to take consent or to document where this is not possible, to start at a low dose and to inform other professionals that the medicine is being used off-licence.

There are two distinct aspects to treating catatonia: specific treatments for catatonia per se and treatments for the disorder(s) underlying catatonia, where identified. While employing either one of these approaches may be effective in some cases, there are many cases where using either one of these strategies alone fails but using the other or a combination of the two is successful (Asnis, 2020; Bogdan et al., 2022; Ekici et al., 2021; Johnson et al., 2022; Lee and House, 2017; Marques Macedo and Gama Marques, 2019; Sundaram et al., 2021). In addition, consideration must be given to the prevention and management of the medical complications of catatonia.

#### First-line treatment

Several studies have found that response to catatonia treatment is more likely or more rapid in patients with a shorter duration of illness (Bush et al., 1996b; Raveendranathan et al., 2012; Shukla et al., 2012; Swain et al., 2017), although this has not universally been the case (Payee et al., 1999). Given this preponderance of evidence and the likely explanation that catatonia becomes less treatment-responsive with time, we recommend treating catatonia as soon as possible after its identification.

In terms of first-line treatments, there is most evidence for benzodiazepines and ECT (Pelzer et al., 2018). We provide more detail about these treatments in sections ‘GABA-ergic pharmacotherapies’ and ‘Electroconvulsive therapy’, but here we consider the question of which to use as first-line therapy. Response rates are similar: 59–100% for ECT and 66–100% in Western studies of benzodiazepines (although some Asian studies found lower response rates) (Pelzer et al., 2018). If one treatment is contraindicated, this makes the decision simpler. Beyond this, consideration should be given to the potential of ECT to ameliorate a disorder underlying the catatonia (NICE recommends ECT for severe depression and prolonged or severe mania in certain circumstances; National Institute for Health and Care Excellence, 2003, 2022), balancing the side effects of ECT (particularly the small risk associated with a general anaesthetic, risk of status epilepticus, post-ictal confusion and autobiographical memory loss) and the side effects of benzodiazepines (particularly respiratory depression, sedation and amnesia). Other considerations more specific to ECT include often limited availability, delays in accessing care, legal issues obtaining consent and patient preferences. There are several studies of ECT after benzodiazepines have been ineffective, reporting high response rates (Bush et al., 1996b; Dutt et al., 2011; Girish and Gill, 2003; Medda et al., 2015). There is a case series and uncontrolled cohort study suggesting that the combination of benzodiazepines and ECT may be effective (Petrides et al., 1997; Unal et al., 2013).

There are several special cases to these recommendations about first-line treatment, which are as follows:

1. Clozapine-withdrawal catatonia: a systematic review of case reports found that restarting clozapine or using ECT were the most effective treatment strategies, while benzodiazepines were less effective (Lander et al., 2018).
2. Benzodiazepine-withdrawal catatonia: a systematic review of case reports found that reinstating benzodiazepines was generally effective (Lander et al., 2018).
3. Catatonia in autism spectrum disorder: see section ‘Autism spectrum disorder’.
4. Chronic, milder catatonia in the context of schizophrenia: there is some evidence that this tends not to respond to benzodiazepines (Ungvari et al., 1999) or ECT (Miller et al., 1953). There is some evidence based on observational data that these patients may respond to clozapine (Saini et al., 2022). There have been rare cases of cardiorespiratory arrest associated with the concomitant use of clozapine and benzodiazepines (Faisal et al., 1997; Saini et al., 2022), so caution should be exercised if there is co-administration.
5. Malignant catatonia: see section ‘Periodic catatonia’.
6. NMS: see section ‘Neuroleptic malignant syndrome’.
7. Antipsychotic-induced catatonia: see section ‘Antipsychotic-induced catatonia’.
8. Women in the perinatal period: see section ‘The perinatal period’.

#### Non-response

Where benzodiazepines or ECT do not succeed in achieving remission of catatonia, it is important to re-evaluate the diagnosis. In one study of 21 patients who entered an RCT for catatonia, 2 of the non-responders were subsequently diagnosed with Parkinson’s disease (Schmider et al., 1999). For alternative treatment approaches, see section ‘Other therapies’.

#### Underlying condition

Alongside treating the catatonia, it is important to treat any underlying disorder. This may involve psychotropic medications (e.g. antidepressants), other medical therapies (e.g. antibiotics, immunosuppressants) or even occasionally surgical treatments (e.g. tumour resection in the case of a paraneoplastic syndrome). Guidelines for treating relevant psychiatric disorders are available from the BAP (Baldwin et al., 2014; Barnes et al., 2020; Cleare et al., 2015; Goodwin et al., 2016; Howes et al., 2018; Lingford-Hughes et al., 2012; O’Brien et al., 2017). There is some controversy over the use of antipsychotic medications in catatonia, which is discussed in section ‘Dopamine receptor antagonists and partial agonists’.

#### Complications

Some, though not all, studies have associated catatonia with an increased mortality (Funayama et al., 2018; Niswander et al., 1963; Rogers et al., 2021). There is an extensive case report literature on the medical complications of catatonia and a large cohort study of patients with schizophrenia found that those with catatonic stupor had an increased risk of various infections (pneumonia, urinary tract infection and sepsis), disseminated intravascular coagulation, rhabdomyolysis, dehydration, deep vein thrombosis, pulmonary embolus, urinary retention, decubitus ulcers, cardiac arrhythmia, renal failure, NMS, hypernatraemia and liver dysfunction (Funayama et al., 2018). Guidance has been developed for averting such complications, which include recommendations such as pharmacological thromboprophylaxis, frequent assessment of pressure areas, stretching to avoid muscle contractures and consideration of artificial feeding (Clinebell et al., 2014; Connell et al., 2022).

*Recommendations on the general approach to treating catatonia*

- Treatment for catatonia should be instituted quickly after identification of catatonia and it is not always necessary to await results of all investigations before commencing treatment. (D)
- Prescribing outside of a product licence is often justified in catatonia, but where a prescriber does this, they should take particular care to provide information to the patient or carer and obtain consent, where possible, taking advice where necessary. (S)
- Catatonia treatment should consist of specific treatment for the catatonia, treatment of any underlying disorder and prevention and management of complications of catatonia. (S)
- First-line treatment for catatonia should usually consist of a trial of benzodiazepines and/or ECT, (C) but see references to special cases in ‘First-line treatment’ and below.
- ECT should be available in any settings where catatonia may be treated, including in psychiatric and general hospitals. (S)
- When deciding between benzodiazepines and ECT as a first-line treatment, consider the following factors: side effect profile, whether there is an underlying disorder that is likely to be responsive to ECT (such as depression or mania) and availability of ECT. (S)
- Where benzodiazepines have not resulted in remission, ECT should be used. (B) For details of what an adequate trial of benzodiazepines consists of, see section ‘GABA-ergic pharmacotherapies’.
- Where catatonia has resulted from clozapine withdrawal, restart clozapine if possible and, if necessary, use ECT. (D)
- Where catatonia has resulted from benzodiazepine withdrawal, restart a benzodiazepine. (D)
- If catatonia is chronic and mild in the context of schizophrenia, consider a trial of clozapine. (C)
- If clozapine and benzodiazepines are administered concomitantly, titrate slowly and closely monitor vital signs. (S)
- Where catatonia does not respond to first-line therapy, re-evaluate the diagnosis. (D)

### GABA-ergic pharmacotherapies

Evidence for pharmacotherapies for catatonia that augment GABA-ergic signalling pathways is supported by neuroimaging studies. Northoff et al. (1999b) conducted an iomazenil GABA-SPECT study and found that patients with catatonia (in a post-acute state) showed significantly lower iomazenil binding in the sensorimotor cortex as well as in the parietal cortex and prefrontal cortex (PFC). The same group was followed up in post-acute catatonia with a subsequent functional MRI (fMRI) study where emotional stimulation was applied before and after lorazepam administration: the orbitofrontal-ventromedial PFC was particularly responsive to a lorazepam challenge, normalising its activity (Richter et al., 2010).

The involvement of the orbitofrontal-ventromedial PFC was further supported by a separate fMRI study where post-acute catatonia patients showed significantly lower emotion-induced activity in this region compared to psychiatric patients without catatonia with the same underlying diagnosis and healthy controls (Northoff et al., 2004). Given that the orbitofrontal-ventromedial PFC is strongly involved in emotion processing, which is mediated by GABA activity, these findings provide further evidence for GABA-ergic mechanisms in catatonia including both GABA-A and GABA-B receptors (Hirjak et al., 2021a; Northoff, 2002; Plevin et al., 2018).

In terms of clinical findings, a double-blind RCT investigated the effect of the barbiturate derivative amobarbital in 1992, finding that of 10 patients randomised to the drug, 6 responded, compared to none of the 10 randomised to a saline infusion (McCall et al., 1992). However, barbiturate use has largely been abandoned since due to safety concerns (López-Muñoz et al., 2005).

Acute catatonia often shows a rapid and dramatic response to benzodiazepines in case series and observational studies (Bush et al., 1996b; Greenfeld et al., 1987; Northoff et al., 1995; Rosebush et al., 1990; Schmider et al., 1999), although a Cochrane review found no placebo-controlled RCTs evaluating benzodiazepines in catatonia (Zaman et al., 2019). Pelzer et al. (2018) reported 17 studies describing benzodiazepine use in patients with catatonia. Most used lorazepam 1–4 mg per day, with some using up to 16 mg per day. Some sources recommend a maximum dose of 24 mg and there are cases of such doses being helpful (Taylor et al., 2021; Weder et al., 2008). Some studies have used other benzodiazepines, such as oxazepam, diazepam, clonazepam or flurazepam (Pelzer et al., 2018) and a small RCT found no difference in outcome between lorazepam and oxazepam treatment (Schmider et al., 1999). However, lorazepam is the most commonly used benzodiazepine for catatonia, it is available in several formulations and its use has a large amount of clinical experience, including at high doses.

Administration can be oral, IM or IV (Bush et al., 1996b; Girish and Gill, 2003; Pelzer et al., 2018). Parenteral administration can be particularly useful if oral administration is not possible, for example due to negativism. Lorazepam is usually administered in 2–4 divided doses each day (Pelzer et al., 2018).

Reported response ranges from 66% up to 100% (Edinoff et al., 2021; Northoff et al., 1995; Pelzer et al., 2018; Rasmussen et al., 2016; Rosebush et al., 1990). These studies were mainly conducted in Western countries. Studies conducted in India and Asia show more variable response, ranging from 0% to 100% (Pelzer et al., 2018). The reason for these differences remains unclear, but it is possible that – given that lorazepam is unstable at room temperature (De Winter et al., 2013; Gottwald et al., 1999) – storage conditions may play a role. Usually, administration of lorazepam is well tolerated, and major side effects are rare. Even a dose as high as 16 mg of lorazepam is often well tolerated without sedation (Pelzer et al., 2018).

Therapeutic response may entail partial or complete remission within hours, though it may sometimes take several days (Lee et al., 2000; Rasmussen et al., 2016; Ungvari et al., 1994). The therapeutic response seems to be strongest in acute catatonia where the patient presents with a rapid-onset catatonic state (Northoff et al., 1995; Northoff et al., 1999a; Pelzer et al., 2018; Rasmussen et al., 2016; Rosebush et al., 1990). This is especially the case in patients suffering from bipolar disorder and major depressive disorder (Rasmussen et al., 2016). In contrast, patients with chronic catatonia, especially in the context of schizophrenia, show a less strong response to lorazepam and are more likely to receive ECT (Pelzer et al., 2018; Rasmussen et al., 2016; Ungvari et al., 1999).

One important issue is the weaning of benzodiazepines. There is a need to balance the therapeutic benefits and the risks of withdrawal effects against dependence and the various risks of long-term benzodiazepine use (Baldwin et al., 2013). Withdrawal schedules for benzodiazepines exist, but these are generally designed for individuals who have been treated with benzodiazepines for months or years (Lader and Kyriacou, 2016), whereas benzodiazepines in catatonia are often used for days or weeks. Nonetheless, such withdrawal schedules are associated with higher retention in treatment and better tolerability than abrupt discontinuation (Denis et al., 2006) and the latter risks potentially fatal withdrawal seizures. In one case series of seven patients who had a relapse of their catatonia on withdrawal of lorazepam (the speed of withdrawal ranging from abrupt discontinuation to dose reduction by 1 mg per week), all had resolution of catatonia once lorazepam was restored to its previous dose and four were able to successfully wean off more slowly over 6 weeks, although three received long-term lorazepam treatment to prevent relapse (Ali et al., 2017). There are other reports of long-term benzodiazepines being used to prevent re-emergence of catatonia (Grover and Aggarwal, 2011; Mader et al., 2020; Saddichha et al., 2007). Therefore, some form of taper seems reasonable and, in the event that catatonia re-emerges following benzodiazepine withdrawal, it is sensible to ensure that an underlying condition has been appropriately treated as well as undertaking a slower taper.

*Recommendations on the use of GABA-ergic medications in catatonia*

- Where benzodiazepines are used for catatonia, available routes of administration may include oral, sublingual, IM and IV. The choice of route should be decided based on clinical appropriateness, rapidity of the required response, patient preference, local experience and availability. (S)
- Where benzodiazepines are used for catatonia, lorazepam is generally the preferred agent. (S)
- Where lorazepam is used for catatonia, high doses above the licensed maximum may be necessary to achieve maximal effect. An adequate trial may be considered complete when catatonia is adequately treated, titration has been stopped due to side effects or dose has reached at least 16 mg per day. (C)
- Benzodiazepines for catatonia should not be stopped abruptly but rather tapered down. The speed of the taper depends on a balance of the therapeutic benefits and the risks of withdrawal effects against the possibility of dependence and the risks of long-term harm from benzodiazepines. (S)
- If catatonia relapses on withdrawal of benzodiazepines, a clinician should ensure that any underlying condition has been adequately treated and a slower taper may be tried. (S)

### Electroconvulsive therapy

The first patients treated with convulsive therapy, both for chemically induced seizures by Meduna in 1934 and for electrically induced seizures by Cerletti and Bini in 1938, had catatonic illnesses (Fink et al., 2022). Since then, governmental authorities, authors of textbooks on ECT or catatonia, and most publications discussing treatment options for catatonia endorse ECT, usually as the most effective treatment even where medications or other interventions have failed. For example, the United States FDA panel endorsed ECT for catatonia under a less restrictive Class 2 safety/efficacy designation (Food and Drug Administration, HHS, 2018) and in the UK NICE recommends ECT for catatonia (National Institute for Health and Care Excellence, 2003). In the UK and many other countries, there are specific legal requirements for administering ECT in a patient who is unable to consent.

Despite this extensive clinical recognition in common practice, a rigorous base of high-quality published evidence is lacking. This deficiency of RCTs arises principally from practical difficulties in conducting sham or placebo treatment arms in people who are usually severely ill with catatonia and often lack an ability to participate in informed-consent processes for such clinical trials.

Among several reviews of existing evidence on ECT for catatonia, the most recent comprehensive one was a meta-analysis (Leroy et al., 2018). Three RCTs involving ECT for patients with catatonia have been conducted, all of which were in patients with primary psychotic disorders (Girish and Gill, 2003; Miller et al., 1953; Phutane et al., 2013). Comparisons were between ECT and risperidone (Girish and Gill, 2003); ECT, sham ECT and sodium thiopental (Miller et al., 1953); and bifrontal ECT and bitemporal ECT (Phutane et al., 2013). Two of the trials were conducted specifically in patients with catatonia (Girish and Gill, 2003; Miller et al., 1953), while one had a catatonic subgroup (Phutane et al., 2013). Unfortunately, none of these contained both standardised ratings for outcome and quantitative results that would allow for statistical determinations of effect size (Leroy et al., 2018). The review did, however, identify 10 studies with such data on quantitative outcomes, but they lacked control groups. Bilateral forms of ECT were the typical treatment modality. From these 10 studies, a meta-analysis showed a standardised mean difference between pre-post severity scores of −3.14, which represents a highly effective treatment. Reported side effects were similar to those seen generally in the use of ECT for depression.

Since Leroy et al.’s review, four additional studies of ECT with pre/post-quantitative outcomes have been published (Perugi et al., 2017; Pierson et al., 2021; Tor et al., 2021; Tripodi et al., 2021). All were naturalistic case series or retrospective analyses, using Clinical Global Impression (CGI) or BFCRS quantitative outcomes. Results ranged from decreases in scores of 40% to 82%, and of response (final CGI ⩽2) rates from 83% to 90%. Pierson et al. studied adolescents ⩽18 years, reporting 90% met the CGI criteria for response (Pierson et al., 2021).

Most published reports describing ECT for catatonia have used bilateral forms of ECT (Leroy et al., 2018), which are generally recommended for severe, medication-resistant or malignant forms of catatonia. No studies were found comparing bilateral versus unilateral ECT for catatonia.

In terms of ECT sessions, most studies captured by Leroy et al.’s review that reported ECT frequency described ECT as taking place three times weekly, although this ranged between daily and twice weekly (Leroy et al., 2018). Number of sessions ranged from 3 to 35 sessions with a mean of 9 sessions (Leroy et al., 2018). There is a lack of data on the superiority of these differing protocols.

*Recommendations for the use of ECT in catatonia*

- Where ECT is administered, bilateral ECT should be considered. (S)
- Where ECT is administered in acute catatonia, it should be given at least two times weekly. (S)
- Number of ECT sessions should be decided on the basis of treatment response, risks and side effects. (S)

### Other therapies

While the majority of patients with catatonia respond robustly to benzodiazepines or ECT, some patients have a partial or non-response (Lee et al., 2000; Pelzer et al., 2018; Rosebush et al., 1990; Unal et al., 2017). Catatonia associated with schizophrenia may be less likely to respond to benzodiazepines (Rosebush and Mazurek, 2010; Ungvari et al., 1999). In addition, benzodiazepines and ECT are cautioned in some circumstances. There are also barriers to ECT use such as legal restrictions and stigma.

These factors have prompted the trialling of several alternative agents, either as monotherapies or as augmentation strategies. The studies examining adjunctive medications for catatonia have consisted of prospective cohort studies, open prospective studies, prospective open label studies, retrospective chart review studies, case series and an open label double blind trial.

#### NMDA receptor antagonists

The NMDA receptor may be allosterically more available to glutamate in catatonia leading to dysfunction in cortico-striato-thalamo-cortical (CSTC) circuits. The NMDA receptor antagonists, amantadine and memantine, may reset the problems related to reduced dopamine and GABA in the CSTC circuitries by balancing NMDA receptor effects on PFC GABA-A parvalbumin interneurons that inhibit PFC pyramidal corticostriatal glutamatergic projections to the striatum while also reducing NMDA action in the striatum itself (Fricchione and Beach, 2019). Medications such as amantadine and memantine serve as uncompetitive antagonists of the NMDA receptor and thus may be helpful in patients with catatonia. Amantadine has the added theoretical benefit of enhancing central dopamine release and delaying dopamine reuptake from the synapse and since catatonia is hypothesised to be to some degree a disorder of hypodopaminergic tone, this profile may also benefit patients with catatonia (Carroll et al., 2007).

In their systematic review, Beach et al (2017) reported on 11 articles that described the use of amantadine in 18 cases (Babington and Spiegel, 2007; Carroll et al., 2006, 2007; de Lucena et al., 2012; Ellul et al., 2015; Ene-Stroescu et al., 2014a, 2014b; Hervey et al., 2012; Muneoka et al., 2010; Northoff et al., 1997; Northoff et al., 1999d). Most patients had schizophrenia spectrum disorders, and some had medical comorbidities. Amantadine as monotherapy often abolished catatonia after a few doses. Five cases involved IV use and the others involved oral dosing. Oral doses ranged from 100 to 600 mg daily, with most patients receiving 200 mg daily. Daily IV doses ranged in 400–600 mg. In 2018, Theibert and Carroll (2018) updated the cases and reported three more amantadine cases that used a mean oral dose of 306 (standard deviation (SD): 189) mg a day. In two of these cases, ECT was also used and in another one the results were equivocal. In a review by Carroll et al. (2007), seven further cases of catatonia, six of whom were diagnosed with schizophrenia, were treated successfully with oral amantadine 200 mg a day. Another patient with atypical psychosis with catatonia showed no improvement with amantadine, though upon removal of amantadine the condition worsened (Brown et al., 1986).

In a clinical study of catatonia in neurologic and psychiatric patients in a tertiary neurological centre, 23 of 42 patients with catatonia related to a neurological disorder received adjunctive amantadine (mean dose 243 (SD: 57) mg/day) most often in addition to first-line oral lorazepam (mean dose 7.3 (SD: 2.8) mg/day) treatment. All patients achieved remission of their catatonia except for two patients who died of encephalitis or encephalomyelitis (Espinola-Nadurille et al., 2016).

In Beach et al.’s (2017) review, nine papers reported memantine treatment in nine cases (Brown et al., 2016; Carpenter et al., 2006; Carroll et al., 2006; Caudron et al., 2016; Mukai et al., 2011; Munoz et al., 2008; Obregon et al., 2011; Thomas, 2005; Utumi et al., 2013). Again schizophrenia-spectrum illnesses were predominantly represented in this sample. Memantine was commonly prescribed as an adjunctive treatment in combination with benzodiazepines. Theibert and Carroll (2018) added three unpublished memantine cases and reported the mean daily dose used for all 12 cases was 12.5 (SD: 6.2) mg.

A few additional articles cite the benefits for catatonia of other medications that may act as glutamate antagonists. These include four cases of minocycline use and one case of dextromethorphan–quinidine use (Carroll et al., 2007; Miyaoka et al., 2007; Theibert and Carroll, 2018; Turner et al., 2016).

In summary, reviews show that in 58 published cases plus other additional reports of amantadine and memantine use in catatonia of various aetiologies, substantial improvement was reported. This improvement usually occurred within a 7-day window (Sienaert et al., 2014). A bias towards the non-reporting of negative results must be considered, making the lack of RCTs and controlled studies an important shortcoming.

#### Dopamine precursors, agonists and reuptake inhibitors

The dopamine system modulates motivation and movement by informing the anterior cingulate cortex/mid-cingulate cortex when a task is associated with high predictive value (tonic dopamine) as well as when circumstances abruptly change to better or worse than predicted (phasic dopamine) (Holroyd and Yeung, 2012). Hong (2013) proposes that the dopamine system in the midbrain ventral tegmental area/substantia nigra functions as a manager of sorts for the CSTC circuits thought to be implicated in catatonia. The dopamine agonists and precursors can be hypothesised to treat catatonia by increasing dopamine modulation and by favouring the striosomal direct pathway as they do in akinetic mutism, leading to opening of the thalamic filter with feedforward activation of cortical regions including the supplementary motor area and primary motor cortex.

Levodopa is a dopamine precursor that is often used in combination with a peripheral DOPA decarboxylase inhibitor (e.g. carbidopa and benserazide) in the treatment of Parkinson’s disease. A case report and small case series found marked improvement after treatment with levodopa, although the case series reported worsening of psychosis (Neppe, 1988; Rogers, 1991). Bromocriptine, a dopamine D_2_ receptor agonist was used successfully in a 16-year-old girl with catatonia (Mahmood, 1991). There is also a literature on the use of dopamine agonists in the related conditions of NMS (see section ‘Neuroleptic malignant syndrome’) and akinetic mutism, a neurological condition associated with lesions to frontal-subcortical circuits (Arnts et al., 2020).

Methylphenidate is a noradrenaline and dopamine reuptake inhibitor. There have been five case reports of successful use of methylphenidate for catatonia (Bajwa et al., 2015; Corchs and Teng, 2010; Frost, 1989; Neuhut et al., 2012; Prowler et al., 2010). Most of these cases were due to mood disorders, and most used methylphenidate as monotherapy.

#### Dopamine receptor antagonists and partial agonists

The use of antipsychotics is one of the most controversial areas in catatonia management (Sienaert et al., 2014). Antipsychotic medications can induce catatonia (see section ‘Antipsychotic-induced catatonia’) and worsen it (Goetz et al., 2013; Lee, 2010; Lewis and Kahn, 2009). Catatonia is also a risk factor for NMS (Berardi et al., 1998; Funayama et al., 2018; Lee, 2010; Rosebush and Mazurek, 2010), a severe antipsychotic-induced movement disorder. Moreover, in some studies of catatonia, the use of antipsychotics has been associated with poor outcomes (Hawkins et al., 1995; Zingela et al., 2022).

Nevertheless, dopamine receptor antagonists and partial agonists have been reported in some cases as beneficial in catatonia (Sienaert et al., 2014; Van den Eede et al., 2005). This may particularly be the case in catatonic schizophrenia (Gazdag and Sienaert, 2013). There have been reports of the use of olanzapine (Babington and Spiegel, 2007; Chang et al., 2009; Kumagai et al., 2016; Nicolato et al., 2006; Numata et al., 2002; Spiegel and Klaiber, 2013; Spiegel and Varnell, 2011; Ueda et al., 2012), risperidone (Ahmed et al., 2009; Duggal, 2005; Girish and Gill, 2003; Grenier et al., 2011; Hesslinger et al., 2001; Serata et al., 2015; Valevski et al., 2001), ziprasidone (Angelopoulos et al., 2010; Levy and Nunez, 2004), quetiapine (Yoshimura et al., 2013) and aripiprazole (Bastiampillai and Dhillon, 2008; Lin et al., 2016; Nakagawa et al., 2012; Sasaki et al., 2012; Voros et al., 2009).

Second-generation antipsychotics (SGAs) theoretically would be less likely to strongly antagonise dopamine receptors making them potentially less dangerous adjunctive treatments than first-generation antipsychotics (FGAs) in terms of NMS risk. Aripiprazole’s partial agonism might balance the dopaminergic effects and be of some benefit for catatonia (Huffman and Fricchione, 2005). A recent Cochrane review found only one RCT of antipsychotics for schizophrenia spectrum disorders with catatonic features, and considered the evidence to be of very low quality due to a small sample size, short duration, risk of bias and other methodological issues (Huang et al., 2022). This RCT compared risperidone to ECT, finding greater improvement in the ECT-treated group (Girish and Gill, 2003).

Given that D_2_ receptor antagonists can worsen catatonia and trigger NMS in an at-risk group, some reviews have urged caution, especially in malignant catatonia (Edinoff et al., 2021; Sienaert et al., 2014; Van den Eede et al., 2005). It has also been suggested that SGAs – or an FGA with weaker dopamine receptor affinity – should be preferred (Edinoff et al., 2021). Some sources suggest that antipsychotics should only be given in catatonia if co-administered with a benzodiazepine (Edinoff et al., 2021). One review concluded that there does not seem to be evidence to support the use of SGAs in patients with catatonia without an underlying psychosis (Pelzer et al., 2018). Two small studies have suggested that low serum iron in catatonia is associated with the development of NMS, leading some to suggest that serum iron may be used in catatonia to predict those who may develop NMS, but the evidence is not of a high quality (Carroll et al., 1995; Lee, 1998).

Regarding clozapine, a systematic review found there is some evidence from case reports and small uncontrolled observational studies that clozapine may be effective in catatonic schizophrenia (Saini et al., 2022). In the largest identified study, 55 patients with catatonic schizophrenia received clozapine, resulting in 2 cases of complete remission, 48 cases of partial remission and 5 cases of no remission (Naber et al., 1992). Where catatonia occurs in the context of clozapine withdrawal, a systematic review of case reports found that re-initiation of clozapine or the use of ECT was usually effective, while benzodiazepines were less reliable (Lander et al., 2018).

#### Anticonvulsants

Leaving aside the cases where catatonia is a presentation of NCSE (Silva Gadelho and Gama Marques, 2022), catatonia has occasionally been treated with anticonvulsant medications. Evidence consists of case series and case reports.

Three articles have reported using carbamazepine to treat catatonia in seven cases (Kritzinger and Jordaan, 2001; Rankel and Rankel, 1988; Spear et al., 1997). Most cases were associated with a mood disorder, and carbamazepine was found to be effective without the need for benzodiazepines. Doses ranged from 100 to 1000 mg daily, with six cases receiving 600 mg daily or greater.

Valproic acid use in catatonia has been reported in four papers in which five patients were suffering with psychoses, mostly schizophrenia spectrum in nature. In three instances, excited catatonia was noted as part of the presentation. These patients were treated successfully with valproic acid (Bowers and Ajit, 2007; Ene-Stroescu et al., 2014b; Krüger and Brӓunig, 2001; Yoshida et al., 2005). Doses ranged from 600 to 4000 mg daily.

Another case series involving four cases highlighted the benefits of topiramate in the treatment of catatonia (McDaniel et al., 2006). Here too most of these patients had been diagnosed with schizophrenia-like illnesses. Topiramate was used as an adjunctive treatment along with a benzodiazepine. All four cases improved on 200 mg daily.

Phenytoin has been reported to be effective in cases where catatonia has appeared in the context of bacterial meningoencephalitis, NCSE and frontal lobe seizures (Coffey, 2013; Lim et al., 1986; Orland and Daghestani, 1987). Levetiracetam and zonisamide have each been used in one case along with aripiprazole (Muneer, 2014; Nakagawa et al., 2012).

#### Anticholinergic agents

Two case reports described using benztropine IV as monotherapy to treat catatonia in two cases (Albucher et al., 1991; Panzer et al., 1990). In another case, trihexyphenidyl was used in combination with clozapine to treat catatonia (Yeh et al., 2004). All patients had a schizophrenia-spectrum illness. And in a fourth case, several medications including trihexyphenidyl were used to treat catatonia in a young woman with Wilson’s disease (Davis et al., 2021).

#### Miscellaneous treatments

Muscle relaxants, calcium channel blockers and corticosteroids have all anecdotally been associated with improvement in isolated patients with catatonia (Philbrick and Rummans, 1994). Lithium and other treatments for prophylaxis in periodic catatonia warrant particular attention and are considered in section ‘Periodic catatonia’.

#### Repetitive transcranial magnetic stimulation and transcranial direct-current stimulation as alternatives to ECT

There are conditions and situations that discourage the use of ECT after non-response to benzodiazepines and second-line agents, and when maintenance ECT is required that offers a potential niche for newer neuromodulatory treatments such as repetitive transcranial magnetic stimulation (rTMS) and transcranial direct-current stimulation (tDCS) for the treatment of catatonia. Two systematic reviews have covered this topic and found that the majority of case reports and case series in the literature reported a positive response (Hansbauer et al., 2020; Stip et al., 2018). rTMS over the bilateral dorsolateral PFC has been particularly emphasised (Stip et al., 2018). Adverse effects appear to be minimal (Hansbauer et al., 2020).

*Recommendations on the use of other therapies*

- Where first-line therapies for catatonia are unavailable, cautioned, ineffective or only partially effective, consider a trial of an NMDA receptor antagonist, either amantadine or memantine. (C)
- Where first-line therapies and NMDA receptor antagonists are unavailable, cautioned, ineffective or only partially effective, consider a trial of levodopa, a dopamine agonist, carbamazepine, valproate, topiramate or a SGA. (D)
- Antipsychotic medications should be avoided where there is no underlying psychotic disorder. (C)
- Where catatonia exists in the context of an underlying psychotic disorder, if antipsychotic medications are used, they should be prescribed with caution after an evaluation of the potential benefits and risks, including the risk of NMS. Additional caution should be exercised if there is low serum iron or a prior history of NMS. If antipsychotic medications are used, a SGA should be used with gradual titration, and co-administration of a benzodiazepine should be considered. (S)
- Where ECT is indicated but unavailable, consider treatment with rTMS or tDCS. (D)

## Subtypes of catatonia and related conditions

### Periodic catatonia

Periodic catatonia is a rare form of catatonia characterised by rapid-onset, brief, recurring episodes of hypokinetic or hyperkinetic catatonia (Hervey et al., 2013). The typical episode may last 4–10 days, with an interepisodic period lasting weeks to years. Kraepelin first described it in the context of schizophrenia. Gjessing extensively studied this entity and published data mainly in German. His work has been summarised by Minde (1966). Leonhard considered periodic catatonia to be a form of unsystematic schizophrenia (i.e. genetically determined schizophrenia) compared to the systematic (nonperiodic and nonfamilial) form of schizophrenia (Beckmann et al., 2000). Based on this conceptualisation, subsequent research showed that periodic catatonia has an autosomal dominant pattern of transmission (Stöber et al., 2000).

Classically periodic catatonia has been reported to occur in association with schizophrenia, but it has also been reported in patients with affective disorders (Barroso Cañizares et al., 1999; Bräunig, 1991; Hervey et al., 2013; Yeh et al., 2010) and occasionally in patients with substance use disorders (Bajaj et al., 2011), underlying medical illnesses (Aragon et al., 2016; Boyce, 1958; Leentjens and Pepplinkhuizen, 1998; Sengul et al., 2005; Sutar and Rai, 2020) and in association with menstrual cycles (Zwiebel et al., 2018). It has also been reported in adolescents (Kinrys and Logan, 2001; Sutar and Rai, 2020) and the geriatric population (Carroll et al., 2011; Tang and Park, 2016). Studies that have focused on the clinical profile of patients with periodic catatonia during the different episodes in the same patients suggest consistency of clinical features across the various episodes (Francis et al., 1997).

There is great uncertainty in the treatment of periodic catatonia, though several sources advise treatment in acute catatonic episodes along the lines of other cases of catatonia with benzodiazepines and ECT (Fink and Taylor, 2001; Ghaffarinejad et al., 2012; Hervey et al., 2013), and several reports support this (Chen and Huang, 2017; Hervey et al., 2013; Saddichha et al., 2007). However, some cases do not respond to these treatments. There is also the important issue of maintenance treatment to prevent catatonic episodes. Lithium is the most frequently reported agent used in the maintenance of periodic catatonia, but even this evidence relies only on case reports and small case series (Gjessing, 1967; Padhy et al., 2011; Petursson, 1976; Sato et al., 2020; Sovner and McHugh, 1974; Wald and Lerner, 1978). Case reports have reported success with mirtazapine (Yeh et al., 2010) and clomipramine (Barroso Cañizares et al., 1999) in the maintenance treatment of periodic catatonia in patients with depressive disorder (Yeh et al., 2010). In another case report, authors reported the effectiveness of fluoxetine (20 mg/day) and fluphenazine in the maintenance treatment of periodic catatonia in a patient with schizoaffective disorder (Białek and Jarema, 1999). Case reports or series have reported the role of lamotrigine (Konstantinou et al., 2021) and carbamazepine (Padhy et al., 2011) in the maintenance treatment of periodic catatonia. Despite the potential risk of NMS when antipsychotics are used in catatonia, some have reported a beneficial role of olanzapine (Guzman et al., 2007, 2008), ziprasidone (Levy and Nunez, 2004) and risperidone (Duggal and Gandotra, 2005) in the long-term treatment of periodic catatonia.

*Recommendation on periodic catatonia*

- In the maintenance phase of periodic catatonia, consider prophylactic treatment with lithium. (D)

### Malignant catatonia

Catatonia may be conceptualised as a continuum, with milder forms at one end (termed simple or benign) and more severe forms, involving hyperthermia and autonomic dysfunction (termed malignant), at the other (Philbrick and Rummans, 1994). Stauder (1934) described ‘lethal catatonia’ as a fulminant psychotic disorder characterised by intense motor excitement, which progressed to stuporous exhaustion, cardiovascular collapse, coma and death. The entire course, passing through excitement into stupor, involved mounting hyperthermia, autonomic instability, delirium, muscle rigidity and prominent catatonic features. The paucity of findings on autopsy was difficult to explain and in sharp contrast to the catastrophic clinical manifestations. This disorder was the subject of numerous publications throughout the pre-antipsychotic drug era. Competing terminology included Bell’s mania, acute delirious mania, pernicious catatonia and delirium acutum, among numerous others. More recently, the term malignant catatonia has been proposed, since not all cases are fatal (Philbrick and Rummans, 1994). Unlike Stauder, some authors have observed that muscle tone in malignant catatonia is flaccid (Mann et al., 1986).

Although the incidence of malignant catatonia appears to have declined following the introduction of modern psychopharmacologic agents, it continues to be reported. Like non-malignant catatonia, malignant catatonia represents a syndrome rather than a specific disease, occurring in association with diverse neuromedical illnesses as well as with psychiatric disorders. Current data suggest that it is likely that a proportion of malignant catatonia cases previously attributed to schizophrenia were more likely the product of autoimmune disorders, particularly anti-NMDAR encephalitis (Mann et al., 2022; Rogers et al., 2019). Mortality, which had exceeded 75% during the pre-antipsychotic drug era, has fallen to 10% in recent reports (Mann et al., 2022).

Although some qualified support may exist for the use of SGAs in non-malignant catatonia (see section ‘Dopamine receptor antagonists and partial agonists’), the literature on antipsychotics for malignant catatonia is rather different. First, there is an issue that malignant catatonia is generally clinically indistinguishable from NMS, so antipsychotics seem injudicious (Philbrick and Rummans, 1994). Second, in a review of 292 malignant catatonia cases (Mann et al., 1986), 78% of those treated with only an antipsychotic died, compared with an overall mortality of 60%. Moreover, this review found that many patients with catatonia developed malignant features only after treatment with antipsychotics (Mann et al., 1986). The evidence for the SGAs in malignant catatonia is minimal and mixed (Van den Eede et al., 2005). Antipsychotic drugs should be withheld whenever malignant catatonia is suspected.

Since RCTs are unavailable, treatment recommendations for malignant catatonia are based on case reports or case series. Five international guidelines for the management of schizophrenia specifically address the treatment of malignant catatonia (Schönfeldt-Lecuona et al., 2020a), although they are based on low levels of evidence. Each of the guidelines recommends ECT either as the initial treatment or as second line after a failed benzodiazepine trial. Although the benefits of benzodiazepines in malignant catatonia are less consistent than in non-malignant catatonia, a review of 44 cases found that there was clear benefit in about a third, transient or partial improvement in a third and no benefit in the remainder (Philbrick and Rummans, 1994), so a benzodiazepine trial seems reasonable. Doses as high as 24 mg of lorazepam per day may be required. However, if benzodiazepines are not rapidly effective, ECT should be started within 48–72 h following the onset of malignant catatonia (Fricchione et al., 1997; Fricchione and Beach, 2019).

ECT appears to be a safe and effective treatment for malignant catatonia occurring in association with a psychiatric disorder. Among 68 patients reported in five series (Mann et al., 2022; Philbrick and Rummans, 1994), 51 of 54 treated with ECT survived, whereas only 6 of 14 who received antipsychotics and supportive care recovered. Still, ECT appears effective only if initiated before severe progression of malignant catatonia. In another series (Arnold, 1952), although 16 of 19 patients receiving ECT within 5 days of malignant catatonia onset survived, none of 14 patients starting ECT beyond that 5-day point recovered. In view of the life-threatening potential of malignant catatonia, bilateral treatments daily or twice daily for 3–5 days are often required to achieve a rapid result, followed by ECT at conventional frequencies until complete resolution (Fink and Taylor, 2003; Petrides et al., 2004). In addition, ECT has been effective as a symptomatic measure in malignant catatonia complicating a diversity of medical conditions, such as anti-NMDAR encephalitis, permitting resolution of the underlying condition.

An older body of case series data had suggested that malignant catatonia could be successfully treated with adrenocorticotropic hormone and corticosteroids (Chrisstoffels and Thiel, 1970; Lingjaerde, 1964). However, the interpretation of these reports may be compromised by the simultaneous use of ECT in many cases. Other proposed treatments have included bromocriptine, amantadine, memantine and calcitonin (Mann et al., 2022). A single case report observed dramatic resolution of malignant catatonia with rTMS (Kate et al., 2011). Although one case report noted rapid improvement in malignant catatonia with tDCS (Haroche et al., 2022), a second found no effect (Baldinger-Melich, 2016). Like non-malignant catatonia, rTMS and tDCS could prove promising in malignant catatonia where ECT is indicated but not possible. However, further investigation is necessary. Finally, a couple of case reports have reported benefit from propofol in malignant catatonia (Alfson et al., 2013; Nomura et al., 2021), which may possibly be useful if ECT is delayed (Hirjak et al., 2019).

*Recommendations for the treatment of malignant catatonia*

- In malignant catatonia, discontinue all dopamine antagonists. (D)
- In malignant catatonia, commence a trial of lorazepam at 8 mg/day (PO, IM or IV), titrating up according to response and tolerability up to a maximum of 24 mg/day. (C)
- If there is partial or no response to lorazepam within 48–72 h in malignant catatonia, institute bilateral ECT once or twice daily for up to 5 days until malignant catatonia abates, followed by ECT three times per week until there is sustained improvement, usually 5–20 treatments in total. (D)

### Neuroleptic malignant syndrome

NMS is a rare and potentially lethal idiosyncratic reaction to treatment with dopamine antagonists. Like malignant catatonia, NMS involves altered consciousness with catatonia, muscle rigidity, hyperthermia and autonomic dysfunction. Recent reports suggest a prevalence of 0.02–0.03%, much lower than the 1–3% reported in the 1980s (Barnes et al., 2020). Mortality has declined over the years to an average of less than 10% (Caroff et al., 2022). Virtually, all classes of drugs that induce dopamine receptor blockade have been implicated in causing NMS, with antipsychotics that have higher affinity for the D_2_ receptor posing the greatest risk (Nielsen et al., 2012). However, SGAs have also been associated with NMS, although they may result in an ‘atypical’ presentation with less severe or absent rigidity or hyperthermia (Caroff et al., 2022). NMS may also occur with dopamine-blocking drugs used as antiemetics, with dopamine depleting drugs, and during dopamine agonist withdrawal. About two-thirds of cases develop within the first 1–2 weeks after drug initiation. Laboratory abnormalities are nonspecific but commonly include elevated serum CK, leucocytosis and low serum iron resembling malignant catatonia.

Several authors have proposed that NMS represents an antipsychotic drug-induced toxic or iatrogenic subtype of malignant catatonia (Fink and Taylor, 2009; Fricchione et al., 1997; Goforth and Carroll, 1995; Koch et al., 2000; Mann et al., 1986; White and Robins, 1991). Two retrospective studies of hospitalised patients meeting stringent criteria for NMS found that, in total, 42 out of 43 episodes also met *DSM-IV* criteria for catatonia (Goforth and Carroll, 1995; Koch et al., 2000). As mentioned in section ‘Dopamine receptor antagonists and partial agonists’, antipsychotic drugs can precipitate and worsen catatonia, while catatonia is a risk factor for NMS. Others, however, have asserted that malignant catatonia and NMS represent two distinct entities, suggesting that excited or agitated behaviour points to malignant catatonia (Castillo et al., 1989; Fleischhacker et al., 1990). A prodromal phase involving agitation and affective disturbance is perhaps more common in malignant catatonia but is not universally present. (Carroll and Taylor, 1981; Fleischhacker et al., 1990; Mann et al., 1986). However, agitation is a common feature of the psychosis preceding NMS for which antipsychotics were originally used. Prominent muscle rigidity has also been proposed as a distinguishing feature (Castillo et al., 1989). Nonetheless, since patients with hyperactivity or psychotic features usually receive medications early in treatment, it may be difficult to know if the presence of rigidity represents NMS or drug-induced extrapyramidal side effects superimposed on malignant catatonia. Furthermore, many malignant catatonia cases in the era prior to antipsychotic therapy did present with rigidity. At a minimum, differentiating between NMS and malignant catatonia where antipsychotic medications have been used is acknowledged to be very challenging (Fleischhacker et al., 1990).

The most important factor in improving survival in NMS is discontinuation of dopamine-blocking medications (Guinart et al., 2021). With cessation of dopamine-blocking drugs and supportive medical care, NMS is in most cases a self-limiting disorder (Mann et al., 2022; Strawn et al., 2007; Woodbury and Woodbury, 1992) with a mean recovery time of 7–10 days. Anticholinergic medications, which impair heat loss through reduction of sweating, should also be discontinued (Mann et al., 2022). Beyond these measures, there is limited consensus regarding the optimal therapeutic approach to NMS. It is difficult to compare specific treatments because NMS is rare, usually self-limiting, and heterogeneous in onset, progression and outcome, which renders RCTs challenging (Caroff et al., 2022; Strawn et al., 2007). Nevertheless, therapies that have been reported as successful in the treatment of NMS include benzodiazepines, dopamine agonists, dantrolene and ECT (Caroff et al., 2022). The use of benzodiazepines for treating NMS is not surprising given the proposed overlap between NMS, catatonia and malignant catatonia. Several case reports and series have found that benzodiazepines have been associated with improvements in some individuals with NMS (Francis et al., 2000; Greenberg and Gujavarty, 1985; Kontaxakis et al., 1990; Lee, 2007; Lew and Tollefson, 1983; Miyaoka et al., 1997), though this response is sometimes transient (Greenberg and Gujavarty, 1985; Lee, 2007; Lew and Tollefson, 1983). However, they are not effective in all patients and one prospective study of 14 episodes of NMS found that while seven out of nine patients with catatonic features responded to benzodiazepines, none of the five patients without catatonic features responded (Lee, 2007). Given that risks are small and benefits possibly marked, several sources suggest a trial of benzodiazepines (Adnet et al., 2000; Berman, 2011; Caroff et al., 1998; Strawn et al., 2007).

Some evidence suggests that NMS results from a reduction of dopaminergic activity in the brain, such that dopamine agonists may reduce that deficit and facilitate resolution of the syndrome (Davis et al., 2000; Mann et al., 2000). Systematic reviews of case reports have found that the dopaminergic medications, bromocriptine and amantadine, are associated with reduced mortality (Sakkas et al., 1991), and bromocriptine is associated with a reduced time to clinical response (Rosenberg and Green, 1989). Although levodopa has been used in only a limited number of reported NMS cases, it was thought to be effective in half the case reports (Sakkas et al., 1991) and dramatic improvements were observed in some cases, even after failure to respond to dantrolene (Nisijima et al., 1997). Newer dopamine agonists developed for transdermal delivery may facilitate administration of dopamine drugs under extreme circumstances (e.g. rotigotine) (Caroff et al., 2022).

Temperature elevation in NMS is theorised to result from antipsychotic drug-induced impairment of central heat loss mechanisms in combination with excess heat production secondary to peripheral hypermetabolism and rigidity of skeletal muscle. Dantrolene, which inhibits contraction and heat production in muscle, may benefit those cases of NMS with extreme temperature elevations, severe rigidity and true hypermetabolism (Caroff et al., 2022). In one systematic review (Sakkas et al., 1991), where dantrolene was used in 101 NMS patients and was the only medication used in 50%, improvement was reported in 81%. Furthermore, mortality was decreased by nearly half compared with supportive care alone (Sakkas et al., 1991). Yamawaki et al. (1993) reported a positive response to dantrolene in 105 (74.5%) of 141 NMS patients. Intravenous dantrolene should not be co-administered with calcium channel blockers (particularly verapamil and diltiazem; amlodipine and nifedipine may be safer alternatives), as hyperkalaemia and cardiovascular collapse can occur (Baxter and Preston, 2022).

The pharmacological agents discussed above are generally effective within the first several days of NMS (Davis et al., 2000). If, despite adequate dosing, a response has not been achieved by 2–3 days, a delayed response is unlikely and ECT should be considered. A review of 40 cases where ECT was used as a treatment primarily for NMS found that there was complete recovery in 25 cases (63%) and partial recovery in a further 11 (28%), although reporting bias is a significant concern (Trollor and Sachdev, 1999). Response often occurs during the first few treatments, although some cases have required multiple ECTs in a single day (McKinney and Kellner, 1997). Furthermore, ECT has the advantages of treating some underlying conditions during acute NMS when antipsychotics must be avoided and in treating a prolonged, residual catatonic or parkinsonian state, which has been observed following NMS (Caroff et al., 2000). Davis et al. (1991) conducted a literature review that found the mortality of 48 NMS patients treated with ECT was 10% compared with 21% for patients treated with supportive care alone. Morcos et al. (2019) retrospectively identified 15 NMS patients treated with ECT at their centre over a 17-year period and reported a mortality rate of 6.7%. ECT should therefore be considered as an initial therapy when NMS is severe and the risk of complications is high. Patients with NMS are not considered at risk for malignant hyperthermia during ECT (Davis et al., 2000). However, succinylcholine can cause hyperkalaemia and arrhythmias in patients with severe rhabdomyolysis, which may explain instances of cardiac complications in NMS patients treated with ECT (Davis et al., 2000). Alternative muscle relaxants should be considered in patients at risk.

Treatment recommendations for NMS are not uniform (Schönfeldt-Lecuona et al., 2020b) and – in the absence of RCTs – any recommendations should be made with caution. In a prospective study of 20 NMS patients, Rosebush et al. (1991) observed that those receiving dantrolene (two patients), bromocriptine (two patients) or both (four patients) had a more prolonged course and more sequalae than those treated only with supportive care, leading the authors to question the efficacy of either agent. More recently, Kuhlwilm et al. (2020) conducted a systematic review of 405 NMS cases comparing patients treated with dantrolene, bromocriptine and ECT with those receiving supportive care alone. Cases were defined as mild, moderate or severe using the Woodbury and Woodbury (1992) criteria. Across the entire sample, independent of severity levels, differences in mortality rates with specific therapies compared to supportive care alone were not statistically significant. However, in severe NMS, mortality rates proved significantly lower with each of dantrolene, bromocriptine and ECT compared to supportive care. The authors concluded that supportive care alone could be sufficient for the treatment of mild to moderate NMS, but that specific therapies were indicated for severe NMS.

A series of international guidelines for the management of schizophrenia contain certain specific recommendations for the treatment of NMS, but these are based on weak levels of evidence and do not consider all relevant treatment options (Schönfeldt-Lecuona et al., 2020a). Schönfeldt-Lecuona et al. (2020a) contend that expert-based treatment algorithms derived from clinical experience, numerous clinical reports and rational theories are of greater value than recommendations provided by the guidelines. These algorithms stress that the specific treatment of NMS be individualised and based on the character, duration and severity or stage of clinical features (Caroff et al., 2022; Davis et al., 2000; Strawn et al., 2007; Woodbury and Woodbury, 1992). In general, the first steps include supportive care and discontinuing dopamine blocking agents and anticholinergics. Benzodiazepines are also widely recommended as an initial intervention for patients with mild NMS characterised by mild rigidity, catatonia or confusion, temperature < 38°C, HR < 100 (Barnes et al., 2020; Caroff et al., 2022; Strawn et al., 2007; Woodbury and Woodbury, 1992). Trials of bromocriptine, amantadine or other dopamine agonists may be a reasonable next step in patients with moderate NMS involving prominent parkinsonian signs and temperatures in the range of 38–40°C. Dantrolene appears beneficial primarily when extreme hyperthermia (>40°C) and severe rigidity develop. Although many patients respond to pharmacotherapy, none of the above medications have been reliably effective in all reported cases of NMS. As reviewed above, ECT may remain effective even late during treatment, as opposed to pharmacotherapies, and after pharmacotherapies have failed (Caroff et al., 2022; Davis et al., 2000; Strawn et al., 2007).

Among patients who recover from NMS, there may be a 30% risk of recurrent episodes following antipsychotic rechallenge (Caroff et al., 2022). However, most patients who require antipsychotics can be safely treated provided measures to reduce risk are followed. Strategies suggested are minimising other risk factors for NMS (such as agitation, medical illness and dehydration), allowing at least 2 weeks from recovery before rechallenge, using a low dose of a SGA with gradual titration and careful monitoring for early signs of NMS (Rosebush and Stewart, 1989; Strawn et al., 2007).

*Recommendations for the treatment of NMS*

- In NMS, discontinue all dopamine antagonists. (C)
- In NMS, discontinue anticholinergic drugs. (S)
- In NMS, supportive care should be provided. This consists of assessment and appropriate management of airway, ventilation, temperature and swallow. Fluid input/output should be monitored, and aggressive fluid resuscitation should be used where required. There should be assessment for hyperkalaemia, renal failure and rhabdomyolysis. There should be careful monitoring for complications such as cardiorespiratory failure, aspiration pneumonia, thromboembolism and renal failure, alongside early consideration of high-dependency care. (S)
- For mild, early NMS, characterised by mild rigidity, catatonia or confusion, temperature < 38°C and HR < 100, consider a trial of lorazepam. (C)
- For moderate NMS, characterised by moderate rigidity, catatonia or confusion, temperature = 38°C–40°C and HR = 100–120, consider a trial of lorazepam. Consider a trial of bromocriptine or amantadine. Consider ECT. (C)
- For severe NMS, characterised by severe rigidity, catatonia or coma, temperature > 40°C and HR > 120, consider a trial of lorazepam and consider dantrolene. Consider bromocriptine or amantadine. Consider ECT. (C).
- If clinical features persist, consider bilateral ECT three times weekly or, in severe cases, once or twice daily, until NMS abates. Continue ECT three times per week until there is sustained improvement to a total of 5–20 treatments. (C)
- Delay restarting antipsychotics by at least 2 weeks after resolution of an NMS episode to reduce the risk of recurrence. (C)

### Antipsychotic-induced catatonia

Antipsychotics with strong dopamine receptor affinity in particular can lead to the development of antipsychotic-induced catatonia (Fricchione et al., 1983; Hirjak, Sartorius, et al., 2021). Antipsychotic-induced catatonia can occur in association with FGAs (Gelenberg and Mandel, 1977; Gugger et al., 2012) and probably less frequently with SGAs (McKeown et al., 2010; Tu et al., 2016; Xiong et al., 2009), and may develop within hours after the first administration of an antipsychotic agent. Diagnosis is often complicated by a question over whether the catatonia is intrinsic to the psychiatric illness or induced by its treatment, the so-called ‘catatonic dilemma’ (Brenner and Rheuban, 1978).

The incidence of and risk factors for antipsychotic-induced catatonia are currently unclear. The catatonic signs of akinesia, stupor and mutism are more often associated with antipsychotics whereas catalepsy and waxy flexibility are less common in antipsychotic-induced catatonia (Gelenberg and Mandel, 1977; Lopez-Canino and Francis, 2007). More complex catatonic behavioural abnormalities, such as echolalia, echopraxia, verbigeration or *Mitgehen*, are not generally reported in association with antipsychotic treatment.

The primary intervention for antipsychotic-induced catatonia is discontinuation of the antipsychotic agent. In some cases, this is sufficient on its own (Gelenberg and Mandel, 1977; McKeown et al., 2010). Other possible options are reducing the dose or switching to an antipsychotic with lower affinity for the dopamine receptors. Benzodiazepines may also be helpful. In one prospective cohort study including 18 patients with antipsychotic-induced catatonia, all were administered lorazepam, of whom 14 had complete remission and 4 had some partial response (Lee, 2010). Of the partial responders, three were administered amantadine, which was associated with a prompt recovery. Anticholinergics were ineffective in six patients before they were administered benzodiazepines. Good response to lorazepam has also been reported in other case series (Fricchione et al., 1983; Gugger et al., 2012). Amantadine has also been reported to be helpful in a case series (Gelenberg and Mandel, 1977).

There is a lack of data on the prophylaxis of antipsychotic-induced catatonia.

*Recommendations for antipsychotic-induced catatonia*

- When catatonia is attributed to antipsychotic administration, consider discontinuing the antipsychotic. (C)
- In more severe cases or cases that do not resolve with antipsychotic discontinuation, consider a trial of a benzodiazepine. (C)
- Once catatonia is treated, if an antipsychotic is still necessary, commence at a low dose and titrate gradually, closely monitoring for side effects. (S)

## Considerations in special groups and situations

### Children and adolescents

The prevalence of catatonia in modern child psychiatry has a wide range from 0.6% to 17%; the lowest prevalence being reported in a French study of adolescents, and the highest in a UK study of young people with autism (Cohen et al., 1999; Wing and Shah, 2000). An Indian study reported an inpatient paediatric prevalence of 5.5% (Thakur et al., 2003). Most cases appear to occur in adolescence: the 119 paediatric cases in a large cohort study had a mean age of 14.6 (SD: 2.7) years, although age ranged from 5 to 17 years (Rogers et al., 2021). The potential aetiologies of catatonia in youth span the same psychiatric and medical categories as adults, and while affective and psychotic processes are most commonly found, appropriate assessment of other potential aetiologies, as detailed in section ‘Clinical assessment’, are indicated based on clinical history, evaluation and examination. In recent years, paediatric catatonia has been increasingly recognised in anti-NMDA receptor encephalitis, being found in over a third of affected children in one study (Sarkis et al., 2019). The Pediatric Catatonia Rating Scale (PCRS) is modified from the BFCRS and has been shown to be applicable in young people (Benarous et al., 2016).

Given the paucity of evidence in children and adolescents, current treatment paradigms are based on those recommended for adults, combined with case reports, case series and international clinical experience. This seems reasonable, particularly where cases occur among adolescents. In terms of benzodiazepines, in a case series of 66 children and adolescents who were hospitalised for catatonia, 51 received benzodiazepines, which were associated with improvement in 33 (65%) (Raffin et al., 2015). The mean dose of lorazepam was 5.4 (SD 3.6) mg/day. A smaller case series of six adolescents with catatonia found IV lorazepam was associated with improvement in all cases (Sorg et al., 2018).

With regard to ECT, a retrospective study of 39 adolescents who received ECT, of whom 17 had catatonia, found that 92% of those with catatonia responded (Grover et al., 2013). In literature reviews, the underlying evidence base is largely case reports and series. In one such review of 59 cases with a range of underlying disorders, at least 45 out of 59 (76%) improved after ECT (Consoli et al., 2010). Another review identified 24 patients with catatonia who had outcome data after ECT, of whom 18 (75%) showed remission or marked improvement (Rey and Walter, 1999). The evidence suggests that side effects of ECT are similar to the adult population and serious complications are very rare (Grover et al., 2013; Rey and Walter, 1999).

Since 2008, several reviews have proposed paediatric catatonia management along the lines of objective catatonia rating scales, medical work-up, removal of offending drugs and lorazepam challenge, followed by lorazepam treatment (sometimes at high doses) and or ECT (Dhossche et al., 2010a; Dhossche and Wachtel, 2013; Hauptman and Benjamin, 2016; Lahutte et al., 2008; Raffin et al., 2015; Withane and Dhossche, 2019).

*Recommendations for catatonia in the children and adolescents*

- Catatonia is known to occur in children as young as 5 years and clinicians should screen for catatonia whenever clinical suspicion exists. (S)
- Evaluation of catatonia aetiologies in children and adolescents should include the same range of disorders as found in adults. (S)
- When assessing for the presence of paediatric catatonia, the PCRS should be used. (C)
- First-line management for paediatric catatonia includes a lorazepam challenge test, lorazepam in increasing doses and bilateral ECT. (D)

### Older adults

The literature on catatonia in the older adult population is limited compared to that in the working-age adult population. As with adult patients, it can be transient or long lasting, varying from weeks to months or years (Jaimes-Albornoz et al., 2022). The studies that have assessed the epidemiology of catatonia among older adults have focused on the acute psychiatric hospital setting, liaison psychiatry setting and intensive care setting (Cuevas-Esteban et al., 2017; Grover et al., 2014; Jaimes-Albornoz et al., 2022; Jaimes-Albornoz and Serra-Mestres, 2013; Kaelle et al., 2016; Serra-Mestres and Jaimes-Albornoz, 2018; Sharma et al., 2017; Takács et al., 2017). The prevalence has varied widely by the study setting and the assessment instruments used.

The phenotype of catatonia among older adults shows a high prevalence of hypokinetic signs, such as immobility/stupor, staring, rigidity, mutism, withdrawal, posturing and negativism (Jaimes-Albornoz et al., 2022), although one study listed excitement among the commonly identified clinical features (Cuevas-Esteban et al., 2017). Catatonia among older adults is often multifactorial in aetiology and a wide range of medical conditions has been implicated, though the outcome is still usually good if it is treated promptly (Jaimes-Albornoz et al., 2022).

Differential diagnosis can be challenging and misdiagnosis of catatonia as delirium, psychosis, stroke, dementia or coma have been reported (Alisky, 2007; Meyen et al., 2018; Ratnakaran et al., 2020). This may result in inappropriate ‘do not resuscitate’ orders (Swartz and Galang, 2001). Reports suggest that medical complications of catatonia, such as deep vein thrombosis, pulmonary embolism and pneumonia may be particular risks in older adults (Hu and Chiu, 2013; Jaimes-Albornoz and Serra-Mestres, 2013; Swartz and Galang, 2001). One small study found that 4 out of 10 older adult patients with catatonia in a liaison psychiatry setting had medical complications and 2 died (Jaimes-Albornoz and Serra-Mestres, 2013).

Benzodiazepines remain the cornerstone of the treatment of catatonia among older adults, although they may respond to lower doses (Ungvari, 1994).

ECT remains the treatment of choice among those not responding to benzodiazepines (Jaimes-Albornoz et al., 2022). Case reports suggest that methylphenidate and zolpidem may also be effective in managing catatonia in older adults. Case reports further suggest a possible beneficial effect of medications including amantadine, memantine, valproate, carbamazepine, topiramate, bromocriptine, propofol, biperiden, bupropion, olanzapine, lithium and tramadol. However, reports are mixed for amantadine, valproate or carbamazepine. Case reports have also reported the beneficial effect of rTMS and tDCS in the management of catatonia (Jaimes-Albornoz et al., 2022).

*Recommendations for catatonia in older adults*

- In older adults, care should be taken to identify medical disorders underlying catatonia. (S)
- Catatonia should be considered in the differential diagnosis for an apparent rapidly progressive dementia or ‘failure to thrive’ clinical presentations in older adults. (S)
- First-line treatment of catatonia in the older adults consists of benzodiazepines, often at lower doses than among younger adults, and ECT. (D)

### The perinatal period

The only systematic study of catatonia in the perinatal period is a retrospective chart review of 200 women consecutively admitted to hospital with postpartum psychosis, which suggests that the condition may be prevalent in women with severe mental illness in the postnatal period: 40 women (20%) were assessed as having catatonic signs (Nahar et al., 2017). The literature in other perinatal groups with psychiatric or medical illnesses does not allow prevalence estimates (Csihi et al., 2022).

In pregnancy, many potential complications of persistent catatonia place the mother and child at exceptionally high risk. These include venous thrombosis and thromboembolism, dehydration, malnutrition, incontinence, infections, communication difficulties, impaired co-operation with assessments and investigations, and impairment of capacity (Clinebell et al., 2014; Csihi et al., 2022; Funayama et al., 2018). Postnatally, the mother’s ability to breastfeed and to care and bond with her infant are key concerns (Csihi et al., 2022).

The sections that follow describe the risks that the two main treatments for catatonia, lorazepam and ECT, may pose to the mother and the child. More details about the general principles of use of psychotropic medications in the perinatal period may be found in the BAP guidance on this topic (McAllister-Williams et al., 2017)

#### The reproductive safety of lorazepam in the perinatal period

Research on the reproductive safety of benzodiazepines remains at an early stage, and studies more typically evaluate benzodiazepines as a group, rather than individual agents. In a meta-analysis of cohort studies of exposure to benzodiazepines, Grigoriadis et al. (2019) found a trend towards increased risks for total (*n* = 5195) and cardiovascular (*n* = 4414) malformations with the lower end of the 95% CI nearly achieving significance. Noh et al. (2022) reported in a nationwide cohort study of 3.1 million pregnancies with a larger sample of benzodiazepine exposures (*n* = 40,846), using propensity scores to account for a large number of potential confounders and several sensitivity analyses, that first trimester exposure to benzodiazepines was associated with a very small increased risk of overall congenital malformations (adjusted relative risk (aRR): 1.09; 95% CI: 1.05–1.13) and specifically, heart defects (adjusted RR: 1.15; 95% CI: 1.10–1.21). A risk of oral clefts, reported by several previous studies (Dolovich et al., 1998), was not confirmed. There were differences between compounds and lorazepam was not associated with significant effects (aRR for overall congenital malformations 1.00, CI: 0.85–1.18; aRR for cardiovascular malformations 1.14, CI: 0.93–1.40).

A systematic review and meta-analysis of prospective studies found that benzodiazepine exposure in pregnancy was associated with increased risks of spontaneous abortion, preterm birth, low birthweight and low Apgar scores with odds ratios of approximately two (Grigoriadis et al., 2020), a value generally regarded as the threshold for clinical significance (Andrade, 2015). These outcomes are determined by other risk factors, many of them associated with mental disorders and difficult to capture from obstetric databases. Therefore, research findings in this area are known to be difficult to interpret and prone to overestimates. The authors highlight this risk of confounding as well as significant heterogeneity in the populations across the included studies. However, the risk of neonatal intensive care unit admission (2.61; CI: 1.64–4.14) was consistently increased and is likely to be related to neonatal benzodiazepine withdrawal.

Cohort studies of neurodevelopmental outcomes following foetal benzodiazepine exposure have been inconclusive (Wang et al., 2022).

A small number of studies suggest that a fully breastfed infant ingests very small amounts of the maternal lorazepam dose (Drugs and Lactation Database (LactMed), 2022; Nishimura et al., 2021), 2022). Clinical observations of infants are scarce but do not report infant sedation or other serious adverse effects following maternal doses within the licensed range (Drugs and Lactation Database (LactMed), 2022; Kelly et al., 2012), but there is a lack of data regarding the effects of the high doses of lorazepam sometimes used in catatonia.

### The use of ECT in the perinatal period

In systematic reviews of the case literature on ECT in the perinatal period (Anderson and Reti, 2009; Calaway et al., 2016; Miller, 1994; Pompili et al., 2014), summarised by Coshal et al (2019), the most common adverse effects attributed to the treatment were foetal bradyarrhythmia, abdominal pain, uterine contractions, premature birth, vaginal bleeding, placental abruption and threatened abortion. In many cases, symptoms were mild and transient (Anderson and Reti, 2009; Miller, 1994). No maternal deaths were reported.

Among 339 cases summarised by Anderson and Reti (2009), 11 foetal or neonatal deaths were reported, one of which was attributed to the treatment: it occurred in the context of maternal status epilepticus following three successive stimuli administered during ECT. Leiknes et al. (2015) found a high rate of complications in their systematic review of case reports and series, including 12 foetal and neonatal deaths among 169 cases. However, the authors did not state whether these outcomes were caused or thought to be caused by ECT. This review included all adverse maternal and foetal outcomes among complications of ECT even if they were highly unlikely to be related to the treatment, such as, for example, anencephaly and other congenital anomalies. This approach led the authors to call for great caution when considering the use of ECT in pregnancy. The authors of the other four systematic reviews (Anderson and Reti, 2009; Calaway et al., 2016; Miller, 1994; Pompili et al., 2014) – while acknowledging the difficulties with interpreting case literature – concluded that ECT is an effective treatment for severe mental illness during pregnancy and that the risks to mother and foetus are relatively low. This view is shared by publications of the Royal College of Psychiatrists (Gregoire and Spoors, 2019), the APA (American Psychiatric Association Committee on Electroconvulsive Therapy, 2001), and the Royal Australian and New Zealand College of Psychiatrists (Weiss et al., 2019).

To achieve the optimal outcomes for mother and child, it is important that professionals with expertise in ECT, perinatal psychiatry and obstetrics are involved in a decision to deliver ECT during pregnancy (Weiss et al., 2019). It is essential that clinicians identify pre-existing risk factors for poor outcomes, appropriately monitor maternal and foetal well-being before, during and after the procedure, and utilise effective preventative interventions. The location and team composition for conducting the ECT and what measures should be taken before, during and after the procedure to prevent maternal and foetal complications depend on the stage of pregnancy (Lakshmana et al., 2014).

There is evidence from three observational studies that ECT is more effective for women with severe affective disorders after childbirth than for non-postnatal patients (Haxton et al., 2016; Reed et al., 1999; Rundgren et al., 2018). The short half-lives of medication used for anaesthesia and muscle relaxation during ECT mean that women should not be prevented from resuming breastfeeding after treatments.

Due to inherent methodological difficulties, considerable uncertainties exist in the evidence, and research findings should be interpreted with caution.

*Recommendations for catatonia in the perinatal period*

- If catatonia is severe and the woman suffers from a mental illness, the psychiatric and obstetric team should make a joint decision as to which inpatient setting is most appropriate for treatment. Contact between the mother and baby should be encouraged as much as is possible and appropriate. Psychiatric care should be provided by a psychiatrist experienced in the management of perinatal mental illness. (S)
- If catatonia is severe and presents high risks to the physical health of the mother and child, and treatment of the underlying condition has been ineffective or would lead to an unacceptable delay, specific treatment for catatonia should be considered. (S)
- The risks of any specific treatment should be carefully weighed against the risks of other treatments or no treatments. (S)

*Recommendations for catatonia during pregnancy*

- Screening and selection of patients for ECT should be conducted by a psychiatrist experienced in ECT, in consultation with both a psychiatrist with appropriate expertise in perinatal psychiatry and an obstetrician. (D)
- If delivery is expected within a few weeks, alternative options, such as induction of labour or Caesarean section should be considered by the obstetrician, anaesthetist, paediatrician and psychiatrist. (S)
- If specific treatment for catatonia is required, lorazepam at doses up to 4 mg/day should be considered initially. (S)
- If lorazepam is not effective at up to 4 mg/day, and the risks to the health of the mother and/or the child are high, the use of ECT can be considered (S)

*Recommendations for catatonia during breastfeeding*

- If treatment with lorazepam at doses higher than 4 mg/day is used, the mother should not breastfeed because of a lack of evidence of its safety. If possible and appropriate, lactation can be maintained during the period of high lorazepam dosing by expressing and discarding milk. (S)
- Women can resume breastfeeding after ECT treatments. (C)

### Autism spectrum disorder

International studies over the past two decades have documented a point prevalence of catatonia ranging from 12% to 20% in individuals with autism, with onset most commonly in adolescence and early adulthood (Billstedt et al., 2005; Breen and Hare, 2017; Wing and Shah, 2000). As the US Center for Disease Control estimated an incidence of autism as 1 in 44 children, it is likely that clinicians will care for individuals with autism and catatonia (Maenner et al., 2021). It is theorised that shared neuronal circuitry and genetic susceptibility loci exist between autism and catatonia (Dhossche et al., 2006b; Dhossche et al., 2005). Catatonia often encompasses the full range of psychomotor retarded and agitated clinical features in autism, and the latter may include dangerous repetitive self-injury with high risk for severe bodily harm (Dhossche and Wachtel, 2013; Wachtel, 2018, 2019).

Diagnosis of catatonia in autism is complicated by the overlap in clinical features between the two conditions (Wing and Shah, 2006). Therefore, several authors have suggested that diagnosis of catatonia in autism should entail a marked change from baseline presentation (Dhossche et al., 2006a; Kakooza-Mwesige et al., 2008; Mazzone et al., 2014; Vaquerizo-Serrano et al., 2021). This is important because no pharmacological or neuromodulatory therapies are indicated for the core symptoms of autism (Howes et al., 2018).

Treatment paradigms are based on case reports and series, as well as international clinical experience. The first blueprints for treatment of catatonia in autism were published by Dhossche et al. (2006a) and begin with standardised assessment of catatonia, taking into consideration baseline autistic features that may mimic catatonia (Dhossche et al., 2006a). Wing and Shah (2000) emphasised that amotivation, prompt dependence, withdrawal and slowness often accompany classic *DSM* catatonia signs in autism, and consideration of catatonia is urged for any change in activity level, self-care or skill. After a catatonia diagnosis and evaluation for underlying medical disorders, clinical features are to be classified as mild, moderate or severe, drawing a clear distinction between impairments such as slowness throughout the day versus immobility, stupor and food refusal. Mild catatonic features may be addressed by the Shah–Wing approach of psychological and supportive interventions with a focus on prompting, structure and stress reduction, and possible lorazepam usage. More severe presentations should be treated with the standard biological anticatatonic regimens including bilateral ECT (Dhossche et al., 2006a). Fink, Taylor and Ghaziuddin offered a medical treatment model in 2006 including catatonia diagnosis with standardised rating scales including the BFCRS, lorazepam trial and ongoing therapy, and bilateral ECT as needed (Fink et al., 2006). In a case series of 22 individuals with catatonia and autism, Wachtel further discussed limited response to benzodiazepines as well optimisation of ECT response, adequate hydration, pre-treatment hyperventilation and limited usage of anaesthetic agents that interfere with seizure threshold (Bailine et al., 1994; Wachtel, 2019). A 2021 systematic review of 12 studies encompassing 969 individuals with autism and catatonia, also noted a lack of clear response to benzodiazepines, which often had to be discontinued due to side effects. This stands in contrast to the overall benefit of benzodiazepines in catatonia in general and is consistent with other reports where ECT was implemented after failed benzodiazepine trials (Bush et al., 1996b; Vaquerizo-Serrano et al., 2021; Wachtel, 2019). The authors also noted that antipsychotics were often used in individuals with catatonia and autism despite a lack of known benefit of such agents in catatonia in general, and urged caution given the risk of worsening catatonia or precipitating its malignant form. Finally, for those patients with autism who require ECT, multiple reports suggest that maintenance ECT may be necessary indefinitely after an index course (Ghaziuddin et al., 2017; Wachtel, 2019; Wachtel et al., 2010).

*Recommendations for catatonia in autism spectrum disorder*

- Clinical vigilance is warranted for the assessment of catatonia in autism spectrum disorder given its high prevalence. (C)
- Diagnosis of catatonia in autism spectrum disorder requires a marked change from baseline presentation. (S)
- First-line interventions in mild cases of catatonia are psychological interventions and/or lorazepam, but the standard treatments for catatonia (i.e. benzodiazepines in escalating dosages and/or bilateral ECT) should be considered in moderate to severe cases. (D)

### Medical conditions

#### Considerations in kidney disease

Catatonia, including malignant catatonia (Nomura et al., 2021), has been described in the context of severe renal impairment (Carroll et al., 1994; Desai et al., 1984; Fekete, 2013), in patients receiving dialysis (Denysenko and Nicolson, 2011) and in the post-transplantation period, often as a result of drug toxicities (Quinn et al., 2014; Sikavi et al., 2019). Patients with renal impairment, even those on dialysis, may still be able to tolerate and benefit from benzodiazepines (Tsai and Huang, 2010) with consideration of the severity of renal impairment, route of administration of benzodiazepines, comorbidities (e.g. frailty), including the risk for delirium.

Typically, no dose adjustments are required even in severe impairment for acute dosing of lorazepam in either oral or parenteral formulation; however, for high or repeated parenteral dosing (Morrison et al., 1984; Verbeeck et al., 1976), monitoring for propylene glycol toxicity and consideration of other therapies such as ECT and NMDA receptor antagonists may be indicated (Goforth, 2007; Quinn et al., 2014) to lessen the impact of the potential side effects of treatment (e.g. falls, confusion, delirium).

#### Considerations in liver disease

Malignant catatonia may be a rare cause of liver failure (Kiparizoska et al., 2021). Catatonia has been reported secondary to Wilson’s disease (Davis et al., 2021), after liver transplantation (Chung et al., 2013; Cottencin et al., 2002; Huang et al., 2006; Kalivas and Bourgeois, 2009; Tatreau et al., 2018), including in post-transplantation delirium (Brown et al., 2016; Kahn, 2016) as well as secondary to post-transplantation drug toxicities (Davis and Tripathi, 2020). The early post-liver transplantation period may be a state of deficiency in GABA signalling (Tatreau et al., 2018), which may place the patient at increased risk for catatonia.

Benzodiazepines may be an effective treatment for catatonia post-transplantation (O’Donnell et al., 2007; Seetharam and Akerman, 2006). In mild to moderate hepatic impairment, typically no dose adjustment for lorazepam is required (oral or parenteral formulations). In severe impairment or failure, use caution (Kraus et al., 1978; Peppers, 1996). Other treatments such as NMDA receptor antagonists (Brown et al., 2016) or ECT may be required when benzodiazepine treatment is cautioned.

#### Considerations in lung disease

Pulmonary complications of catatonia may include pulmonary embolism, aspiration pneumonia, pneumothorax, bronchorrhoea, central hypoventilation, respiratory failure and delayed weaning from mechanical ventilation (Funayama et al., 2018; Gupta et al., 2015; Hayashi et al., 2006; ter Haar et al., 2006; Thomas et al., 1994).

Catatonia has been described in the context of respiratory illnesses, including influenza (Coulonjou et al., 1958; Wender, 1979) and SARS-CoV-2 (Amouri et al., 2020; Brandão et al., 2021; Caan et al., 2020; Cooper and Ross, 2020; Ghaziuddin et al., 2021; Gouse et al., 2020; Kaur et al., 2021; Kopishinskaia et al., 2021; Kwon et al., 2021; Mulder et al., 2021; Naik et al., 2021; Nikayin et al., 2022; Raidurg et al., 2021; Scheiner et al., 2021; Torrico et al., 2021; Vazquez-Guevara et al., 2021; Zain et al., 2021; Zandifar and Badrfam, 2020), as well as in critical illnesses (e.g. sepsis, shock). Catatonia in the context of critical illness including respiratory failure may have high comorbidity with delirium (Grover et al., 2014; Wilson et al., 2017). Respiratory failure due to malignant catatonia has been described and may be especially responsive to ECT (Barnardo et al., 2007; Boyarsky et al., 1999; Hayashi et al., 2006; ter Haar et al., 2006; Thomas et al., 1994), especially in those unable to tolerate a benzodiazepine (Geretsegger and Rochowanski, 1987).

*Recommendations for catatonia in kidney, liver and lung disease*

- In renal impairment, lorazepam dosing does not usually need to be altered, but consider additional monitoring for side effects. (C)
- In mild or moderate hepatic impairment, lorazepam dosing does not usually need to be altered, but caution should be exercised when considering lorazepam in severe hepatic impairment. (B)
- In severe respiratory disease, consider giving ECT as a first-line treatment rather than benzodiazepines. (D)

## Research priorities

One general point for future research is that there is a need to harmonise definitions of catatonia and definitions of specific catatonic signs, as well as thresholds for making a diagnosis (Oldham, 2022).

As this guideline has highlighted, the most urgent research goal for catatonia is to develop a more robust evidence base for its treatment. Despite the wealth of small reports and observational data, a Cochrane systematic review of the use of benzodiazepines for catatonia found that no RCT met its inclusion criteria (Zaman et al., 2019). Although some might consider an RCT infeasible in catatonia, the Cochrane review found several examples. Unfortunately, though, these studies had methodological issues, introducing questions of validity. Conducting clinical trials in catatonia is an important priority for psychiatric research in the next decade.

In the meantime, there is substantial scope to improve the quality of evidence for the treatment of catatonia by using large databases of electronic healthcare records with prescribing data. Additional measures to improve the evidence base would include harmonising the outcomes used in research studies by developing a set of core outcomes. This would facilitate pooling of data across research centres, which is an important tool in researching less common conditions.

We provide a list of priority research questions in Table 11.

**Table 11.** Key research questions.

What are the boundaries of the catatonic syndrome? Specifically, can validation by a lorazepam challenge test confirm how mild or severe the phenotype is?
Given that the last two decades are continuing to reveal medical aetiologies of catatonia, what other medical conditions might account for catatonia?
Do the associations of various neurological and medical conditions with catatonia identified in case reports and series hold in larger observational studies?
What are the genetic or environmental factors that predispose certain individuals to the development of drug-induced catatonia or NMS?
Does the use of benzodiazepines in catatonia stand up to rigorous clinical trial methodology?
How long after recovery should patients with catatonia be treated with benzodiazepines?
What are the optimal conditions for ECT treatment, in terms of electrode placement, frequency and timing?
What is the reproductive safety of ECT?
How might the parameters of rTMS be optimised for catatonia?
What treatments are effective in individuals who do not respond to benzodiazepines or ECT?
What is the role of antipsychotic medications in the treatment of individuals with catatonia?
Are there any psychosocial treatments that might support the prevention or treatment of catatonia?
How does the pathophysiology and treatment of catatonia in children, adolescents and older adults differ from those of younger adults?

ECT: electroconvulsive therapy; NMS: neuroleptic malignant syndrome; rTMS: repetitive transcranial magnetic stimulation.

## Supplemental Material

sj-docx-1-jop-10.1177_02698811231158232 – Supplemental material for Evidence-based consensus guidelines for the management of catatonia: Recommendations from the British Association for Psychopharmacology Supplemental material, sj-docx-1-jop-10.1177_02698811231158232 for Evidence-based consensus guidelines for the management of catatonia: Recommendations from the British Association for Psychopharmacology by Jonathan P Rogers, Mark A Oldham, Gregory Fricchione, Georg Northoff, Jo Ellen Wilson, Stephan C Mann, Andrew Francis, Angelika Wieck, Lee Elizabeth Wachtel, Glyn Lewis, Sandeep Grover, Dusan Hirjak, Niraj Ahuja, Michael S Zandi, Allan Young, Kevin Fone, Simon Andrews, David Kessler, Tabish Saifee, Siobhan Gee, David S Baldwin and Anthony S David in Journal of Psychopharmacology

sj-docx-2-jop-10.1177_02698811231158232 – Supplemental material for Evidence-based consensus guidelines for the management of catatonia: Recommendations from the British Association for Psychopharmacology Supplemental material, sj-docx-2-jop-10.1177_02698811231158232 for Evidence-based consensus guidelines for the management of catatonia: Recommendations from the British Association for Psychopharmacology by Jonathan P Rogers, Mark A Oldham, Gregory Fricchione, Georg Northoff, Jo Ellen Wilson, Stephan C Mann, Andrew Francis, Angelika Wieck, Lee Elizabeth Wachtel, Glyn Lewis, Sandeep Grover, Dusan Hirjak, Niraj Ahuja, Michael S Zandi, Allan Young, Kevin Fone, Simon Andrews, David Kessler, Tabish Saifee, Siobhan Gee, David S Baldwin and Anthony S David in Journal of Psychopharmacology

sj-pptx-3-jop-10.1177_02698811231158232 – Supplemental material for Evidence-based consensus guidelines for the management of catatonia: Recommendations from the British Association for Psychopharmacology Supplemental material, sj-pptx-3-jop-10.1177_02698811231158232 for Evidence-based consensus guidelines for the management of catatonia: Recommendations from the British Association for Psychopharmacology by Jonathan P Rogers, Mark A Oldham, Gregory Fricchione, Georg Northoff, Jo Ellen Wilson, Stephan C Mann, Andrew Francis, Angelika Wieck, Lee Elizabeth Wachtel, Glyn Lewis, Sandeep Grover, Dusan Hirjak, Niraj Ahuja, Michael S Zandi, Allan Young, Kevin Fone, Simon Andrews, David Kessler, Tabish Saifee, Siobhan Gee, David S Baldwin and Anthony S David in Journal of Psychopharmacology
