# Supplementary material for: Evidence-based consensus guidelines for the management of catatonia: Recommendations from the British Association for Psychopharmacology
Source: J Psychopharmacol. 2023 Apr 11;37(4):327–69. doi: 10.1177/02698811231158232 (PMC10101189; doi:10.1177/02698811231158232)
Supplement: sj-docx-1-jop-10.1177_02698811231158232 – Supplemental material for Evidence-based consensus guidelines for the management of catatonia: Recommendations from the British Association for Psychopharmacology [file sj-docx-1-jop-10.1177_02698811231158232.docx]

#
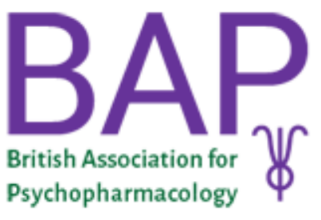
Evidence-based consensus guidelines for the management of catatonia: recommendations from the British Association for Psychopharmacology

List of recommendations

2023

For an explanation of the strength of recommendations (A, B, C, D and S), see Table 1 in the main guideline document.

## Definition of catatonia

- Catatonia should be diagnosed based on the presence of three or more catatonic signs, as in DSM-5-TR or ICD-11. (B)

## Assessment of catatonia

## Initial assessment and treatment of catatonia should be conducted within secondary care. (S)

## Catatonia should be considered as a differential diagnosis whenever a patient exhibits a substantially altered level of activity or abnormal behaviour, especially where it is grossly inappropriate to the context. (D)

## A collateral history should be sought wherever possible. (S)

## The history should include identification of possible medical and psychiatric disorders underlying catatonia, as well as prior response to treatment. (S)

## Physical examination should include assessment for catatonic signs, signs of medical conditions that may have led to the catatonia and signs of medical complications of catatonia. (D)

## When assessing a patient with catatonia, clinicians should interact with the person as if they are able to understand what is being said to them. (S)

## In an individual who is suspected to have catatonia, non-engagement with clinical assessment should not automatically be assumed to be wilful. Mental capacity to engage in an assessment should be assessed and, if found lacking, consideration should be given to acting in an individual’s best interests within the appropriate legal framework. (S)

## Rating instruments

## When assessing for the presence of catatonia or its response to treatment, a validated instrument such as the Bush-Francis Catatonia Rating Scale or the Northoff Catatonia Rating Scale should be used. (C)

## Research on catatonia should report how individual items have been defined, including thresholds. (S)

## Investigations in catatonia

## Investigations, such as blood tests, urine drug screen, lumbar puncture, electroencephalography, and neuroimaging, should be considered based on history and examination findings, taking into account the possible diagnoses that may mimic catatonia and the possible underlying aetiology of the catatonia. (D)

## In patients experiencing a first episode of catatonia or where the diagnosis underlying catatonia is unclear, consider a computed tomography (CT) or magnetic resonance imaging (MRI) scan of the brain. (C)

## In patients experiencing a first episode of catatonia or where the diagnosis underlying catatonia is unclear, consider assessing for the presence of antibodies to the NMDA receptor and other relevant autoantibodies in serum and cerebrospinal fluid. (D)

## In patients with risk factors for seizures, possible evidence of a seizure or possible encephalitis, consider performing an electroencephalogram (with continuous monitoring if available). (C)

## Challenge tests

## When a diagnosis of catatonia is uncertain, a diagnostic challenge using lorazepam should be considered. (B)

## When a diagnosis of catatonia is uncertain, a diagnostic challenge using zolpidem may be considered. (C)

## In suspected or confirmed cases of catatonia, a lorazepam challenge may be used to predict future response to benzodiazepines. (B)

## General approach to treating catatonia

## Treatment for catatonia should be instituted quickly after identification of catatonia and it is not always necessary to await results of all investigations before commencing treatment. (D)

## Prescribing outside of a product licence is often justified in catatonia, but where a prescriber does this, they should take particular care to provide information to the patient or carer and obtain consent, where possible, taking advice where necessary. (S)

## Catatonia treatment should consist of specific treatment for the catatonia, treatment of any underlying disorder, and prevention and management of complications of catatonia. (S)

## First-line treatment for catatonia should usually consist of a trial of benzodiazepines and/or ECT, (C) but see references to special cases in 7.1.1 and below.

## ECT should be available in any settings where catatonia may be treated, including in psychiatric and general hospitals. (S)

## When deciding between benzodiazepines and ECT as a first-line treatment, consider the following factors: side effect profile, whether there is an underlying disorder that is likely to be responsive to ECT (such as depression or mania) and availability of ECT. (S)

## Where benzodiazepines have not resulted in remission, ECT should be used. (B) For details of what an adequate trial of benzodiazepines consists of, see section 7.2.

## Where catatonia has resulted from clozapine withdrawal, restart clozapine if possible and, if necessary, use ECT. (D)

## Where catatonia has resulted from benzodiazepine withdrawal, restart a benzodiazepine. (D)

## If catatonia is chronic and mild in the context of schizophrenia, consider a trial of clozapine. (C)

## If clozapine and benzodiazepines are administered concomitantly, titrate slowly and closely monitor vital signs. (S)

## Where catatonia does not respond to first-line therapy, re-evaluate the diagnosis. (D)

## GABA-ergic medications in catatonia

## Where benzodiazepines are used for catatonia, available routes of administration may include oral, sublingual, IM and IV. The choice of route should be decided based on clinical appropriateness, rapidity of the required response, patient preference, local experience and availability. (S)

## Where benzodiazepines are used for catatonia, lorazepam is generally the preferred agent. (S)

## Where lorazepam is used for catatonia, high doses above the licensed maximum may be necessary to achieve maximal effect. An adequate trial may be considered complete when catatonia is adequately treated, titration has been stopped due to side effects or dose has reached at least 16 mg per day. (C)

## Benzodiazepines for catatonia should not be stopped abruptly but rather tapered down. The speed of the taper depends on a balance of the therapeutic benefits and the risks of withdrawal effects against the possibility of dependence and the risks of long-term harm from benzodiazepines. (S)

## If catatonia relapses on withdrawal of benzodiazepines, a clinician should ensure that any underlying condition has been adequately treated and a slower taper may be tried. (S)

## Electroconvulsive therapy (ECT) in catatonia

## Where ECT is administered, bilateral ECT should be considered. (S)

## Where ECT is administered in acute catatonia, it should be given at least 2 times weekly. (S)

## Number of ECT sessions should be decided on the basis of treatment response, risks and side effects. (S)

## Other therapies in catatonia

## Where first-line therapies for catatonia are unavailable, cautioned, ineffective or only partially effective, consider a trial of an NMDA receptor antagonist, either amantadine or memantine. (C)

## Where first-line therapies and NMDA receptor antagonists are unavailable, cautioned, ineffective or only partially effective, consider a trial of levodopa, a dopamine agonist, carbamazepine, valproate, topiramate or a second-generation antipsychotic. (D)

## Antipsychotic medications should be avoided where there is no underlying psychotic disorder. (C)

## Where catatonia exists in the context of an underlying psychotic disorder, if antipsychotic medications are used, they should be prescribed with caution after an evaluation of the potential benefits and risks, including the risk of NMS. Additional caution should be exercised if there is low serum iron or a prior history of NMS. If antipsychotic medications are used, a second-generation antipsychotic should be used with gradual titration, and co-administration of a benzodiazepine should be considered. (S)

## Where ECT is indicated but unavailable, consider treatment with rTMS or tDCS. (D)

## Periodic catatonia

- In the maintenance phase of periodic catatonia, consider prophylactic treatment with lithium. (D)

## Treatment of malignant catatonia

## In malignant catatonia, discontinue all dopamine antagonists. (D)

## In malignant catatonia, commence a trial of lorazepam at 8 mg / day (PO, IM or IV), titrating up according to response and tolerability up to a maximum of 24 mg / day. (C)

## If there is partial or no response to lorazepam within 48 to 72 hours in malignant catatonia, institute bilateral ECT once or twice daily for up to 5 days until malignant catatonia abates, followed by ECT 3 times per week until there is sustained improvement, usually 5 to 20 treatments in total. (D)

## Treatment of neuroleptic malignant syndrome (NMS)

## In NMS, discontinue all dopamine antagonists. (C)

## In NMS, discontinue anticholinergic drugs. (S)

## In NMS supportive care should be provided. This consists of assessment and appropriate management of airway, ventilation, temperature and swallow. Fluid input/output should be monitored, and aggressive fluid resuscitation should be used where required. There should be assessment for hyperkalaemia, renal failure and rhabdomyolysis. There should be careful monitoring for complications such as cardiorespiratory failure, aspiration pneumonia, thromboembolism and renal failure, alongside early consideration of high-dependency care. (S)

## For mild, early NMS, characterised by mild rigidity, catatonia or confusion, temperature < 38⁰C and HR < 100, consider a trial of lorazepam. (C)

## For moderate NMS, characterised by moderate rigidity, catatonia or confusion, temperature 38 – 40⁰C and HR 100 – 120, consider a trial of lorazepam. Consider a trial of bromocriptine or amantadine. Consider ECT. (C)

## For severe NMS, characterised by severe rigidity, catatonia or coma, temperature > 40⁰C and HR > 120, consider a trial of lorazepam and consider dantrolene. Consider bromocriptine or amantadine. Consider ECT. (C).

## If clinical features persist, consider bilateral ECT 3 times weekly or, in severe cases, once or twice daily, until NMS abates. Continue ECT 3 times per week until there is sustained improvement to a total of 5 – 20 treatments. (C)

## Delay restarting antipsychotics by at least 2 weeks after resolution of an NMS episode to reduce the risk of recurrence. (C)

## Antipsychotic-induced catatonia

## When catatonia is attributed to antipsychotic administration, consider discontinuing the antipsychotic. (C)

## In more severe cases or cases that do not resolve with antipsychotic discontinuation, consider a trial of a benzodiazepine. (C)

## Once catatonia is treated, if an antipsychotic is still necessary, commence at a low dose and titrate gradually, closely monitoring for side effects. (S)

## Catatonia in children and adolescents

## Catatonia is known to occur in children as young as five years and clinicians should screen for catatonia whenever clinical suspicion exists. (S)

## Evaluation of catatonia aetiologies in children and adolescents should include the same range of disorders as found in adults. (S)

## When assessing for the presence of paediatric catatonia, the Pediatric Catatonia Rating Scale should be used. (C)

## First-line management for paediatric catatonia includes a lorazepam challenge test, lorazepam in increasing doses and bilateral ECT. (D)

## Catatonia in older adults

## In older adults, care should be taken to identify medical disorders underlying catatonia. (S)

## Catatonia should be considered in the differential diagnosis for an apparent rapidly progressive dementia or ‘failure to thrive’ clinical presentations in older adults. (S)

## First-line treatment of catatonia in the older adults consists of benzodiazepines, often at lower doses than among younger adults, and ECT. (D)

## Catatonia in the perinatal period

## If catatonia is severe and the woman suffers from a mental illness, the psychiatric and obstetric team should make a joint decision as to which inpatient setting is most appropriate for treatment. Contact between the mother and baby should be encouraged as much as is possible and appropriate. Psychiatric care should be provided by a psychiatrist experienced in the management of perinatal mental illness. (S)

## If catatonia is severe and presents high risks to the physical health of the mother and child, and treatment of the underlying condition has been ineffective or would lead to an unacceptable delay, specific treatment for catatonia should be considered. (S)

## The risks of any specific treatment should be carefully weighed against the risks of other treatments or no treatments. (S)

## Catatonia during pregnancy

## Screening and selection of patients for ECT should be conducted by a psychiatrist experienced in ECT, in consultation with both a psychiatrist with appropriate expertise in perinatal psychiatry and an obstetrician. (D)

## If delivery is expected within a few weeks, alternative options, such as induction of labour or Caesarean section should be considered by the obstetrician, anaesthetist, paediatrician and psychiatrist. (S)

## If specific treatment for catatonia is required, lorazepam at doses up to 4 mg / day should be considered initially. (S)

## If lorazepam is not effective at up to 4 mg / day, and the risks to the health of the mother and/or the child are high, the use of ECT can be considered (S)

## Catatonia during breastfeeding

## If treatment with lorazepam at doses higher than 4 mg / day is used, the mother should not breastfeed because of a lack of evidence of its safety. If possible and appropriate, lactation can be maintained during the period of high lorazepam dosing by expressing and discarding milk. (S)

## Women can resume breastfeeding after ECT treatments. (C)

## Catatonia in autism spectrum disorder

## Clinical vigilance is warranted for the assessment of catatonia in autism spectrum disorder given its high prevalence. (C)

## Diagnosis of catatonia in autism spectrum disorder requires a marked change from baseline presentation. (S)

## First-line interventions in mild cases of catatonia are psychological interventions and/or lorazepam, but the standard treatments for catatonia (i.e. benzodiazepines in escalating dosages and/or bilateral ECT) should be considered in moderate to severe cases. (D)

## Catatonia in kidney, liver and lung disease

- In renal impairment, lorazepam dosing does not usually need to be altered, but consider additional monitoring for side effects. (C)
- In mild or moderate hepatic impairment, lorazepam dosing does not usually need to be altered, but caution should be exercised when considering lorazepam in severe hepatic impairment. (B)
- In severe respiratory disease, consider giving ECT as a first-line treatment rather than benzodiazepines. (D)
