# Supplementary material for: Evidence-based consensus guidelines for the management of catatonia: Recommendations from the British Association for Psychopharmacology
Source: J Psychopharmacol. 2023 Apr 11;37(4):327–69. doi: 10.1177/02698811231158232 (PMC10101189; doi:10.1177/02698811231158232)
Supplement: sj-docx-2-jop-10.1177_02698811231158232 – Supplemental material for Evidence-based consensus guidelines for the management of catatonia: Recommendations from the British Association for Psychopharmacology [file sj-docx-2-jop-10.1177_02698811231158232.docx]

# Evidence-based consensus guidelines for the management of catatonia: recommendations from the British Association for Psychopharmacology

Plain language summary

Catatonia is a condition in which a person has problems with their movement, speech, behaviour and emotions. They might be stuck in one position or unable to speak. It can occur in some mental disorders, such as major depression, bipolar affective disorder and schizophrenia, but it may also occur in some medical conditions, such as brain inflammation or infections.

Although a person with catatonia may not be able to respond, they are often aware of their surroundings and what is being said, so the way in which you communicate with them is very important.

When a clinician assesses a person with catatonia, they should ask the person (and their carers) questions about what has been happening. They should also physically examine the person, which might involve trying to move their arms or listening to their chest. Depending on what they find, it might be appropriate to request tests such as a blood test or brain scan.

There might be treatments that need to be given for a person’s mental or physical illness, but there are also treatments specifically for catatonia. The most common treatment is a group of sedative medications called ‘benzodiazepines’ (“BEN-zoh-dy-A-zuh-peens”). In catatonia, benzodiazepines work for most people.

Some people with catatonia should be given a treatment known as electroconvulsive therapy (or ECT). This involves a person being given a general anaesthetic to put them to sleep. Then an electrical current is given, which causes a seizure. This is very effective for relieving catatonia in most people. In the UK, there are formal procedures that must be followed before a person is given ECT when they are unable to consent.

However, there is still a lot that we do not know about catatonia. Much of what we recommend is based on years of clinical experience, rather than rigorous research studies, so there is a need to research catatonia a lot more.
