# Supplementary material for: Evidence-based consensus guidelines for the management of catatonia: Recommendations from the British Association for Psychopharmacology
Source: J Psychopharmacol. 2023 Apr 11;37(4):327–69. doi: 10.1177/02698811231158232 (PMC10101189; doi:10.1177/02698811231158232)
Supplement: sj-pptx-3-jop-10.1177_02698811231158232 – Supplemental material for Evidence-based consensus guidelines for the management of catatonia: Recommendations from the British Association for Psychopharmacology [file sj-pptx-3-jop-10.1177_02698811231158232.pptx]

## Slide 1
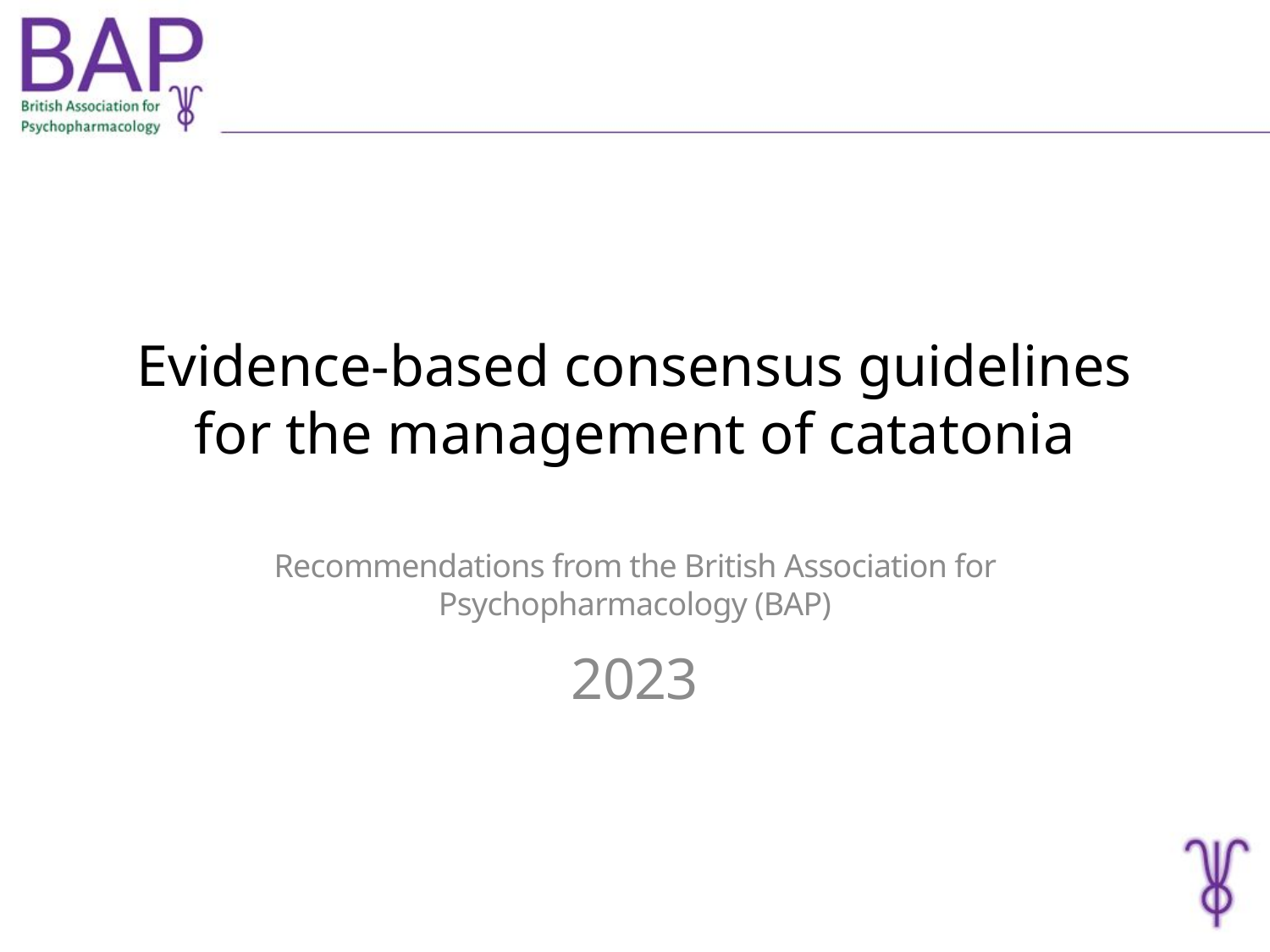

# Evidence-based consensus guidelines for the management of catatonia
Recommendations from the British Association for Psychopharmacology (BAP)
2023

## Slide 2
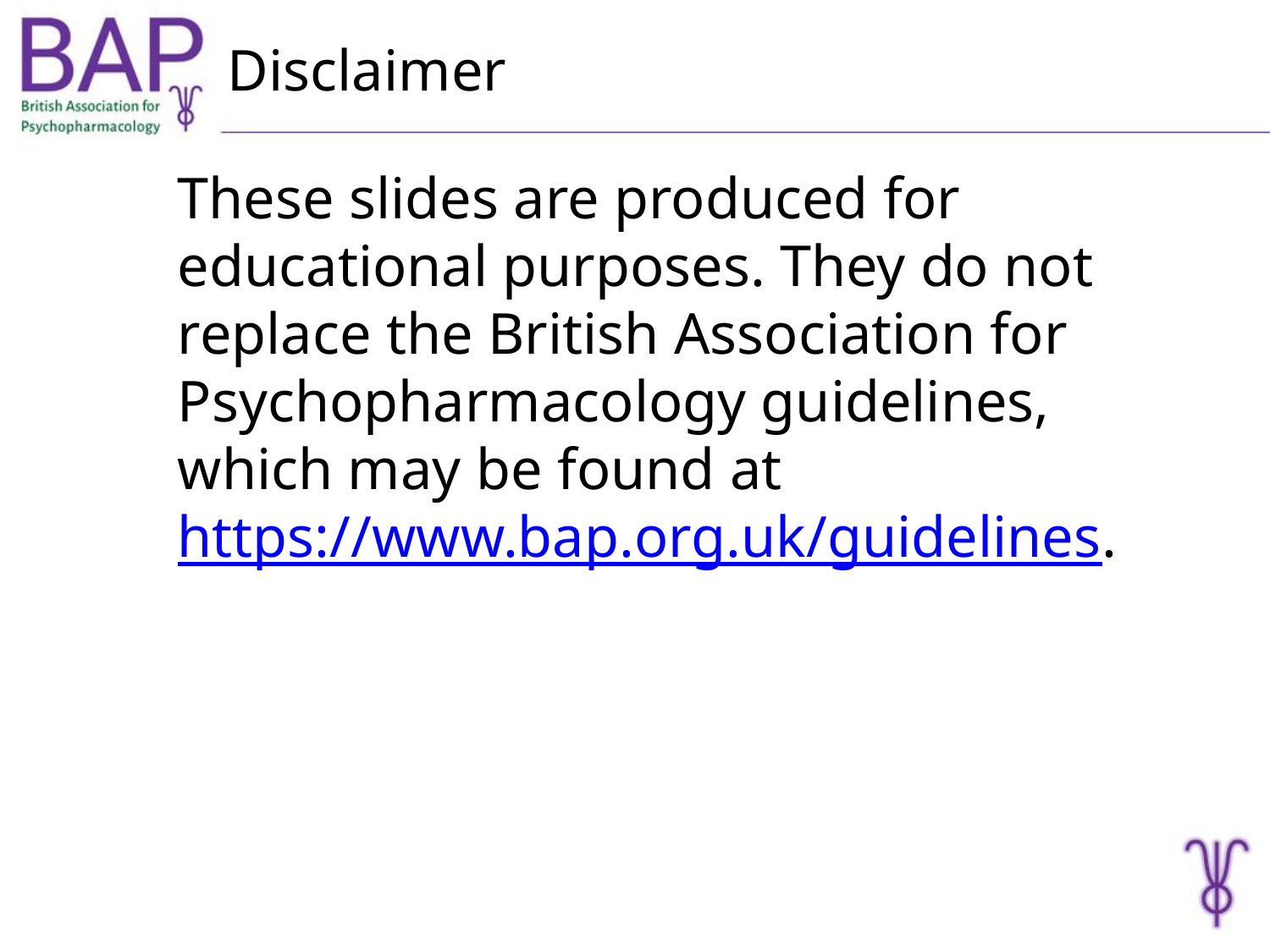

# Disclaimer
These slides are produced for educational purposes. They do not replace the British Association for Psychopharmacology guidelines, which may be found at https://www.bap.org.uk/guidelines.

## Slide 3
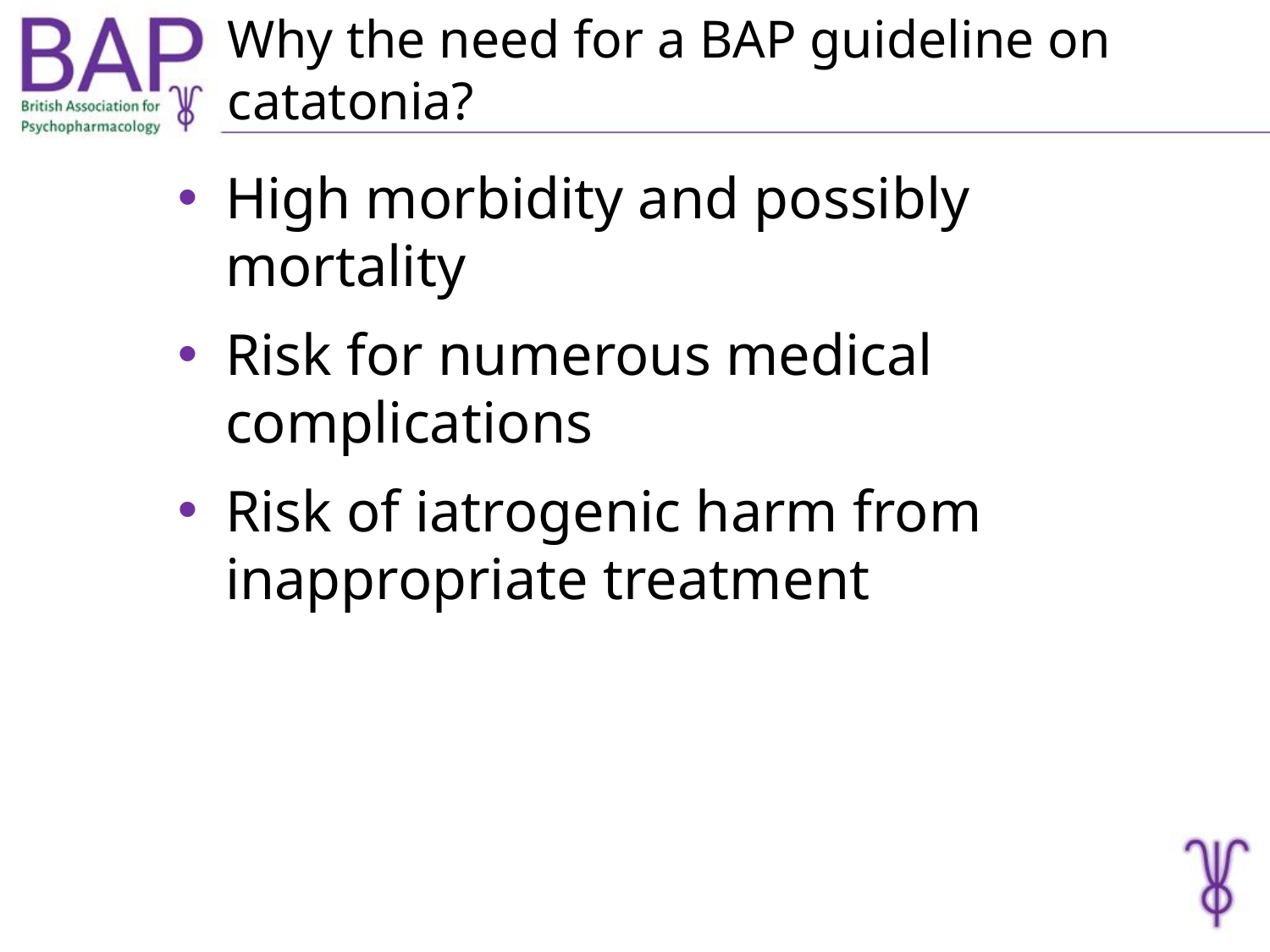

# Why the need for a BAP guideline on catatonia?
High morbidity and possibly mortality
Risk for numerous medical complications
Risk of iatrogenic harm from inappropriate treatment

## Slide 4
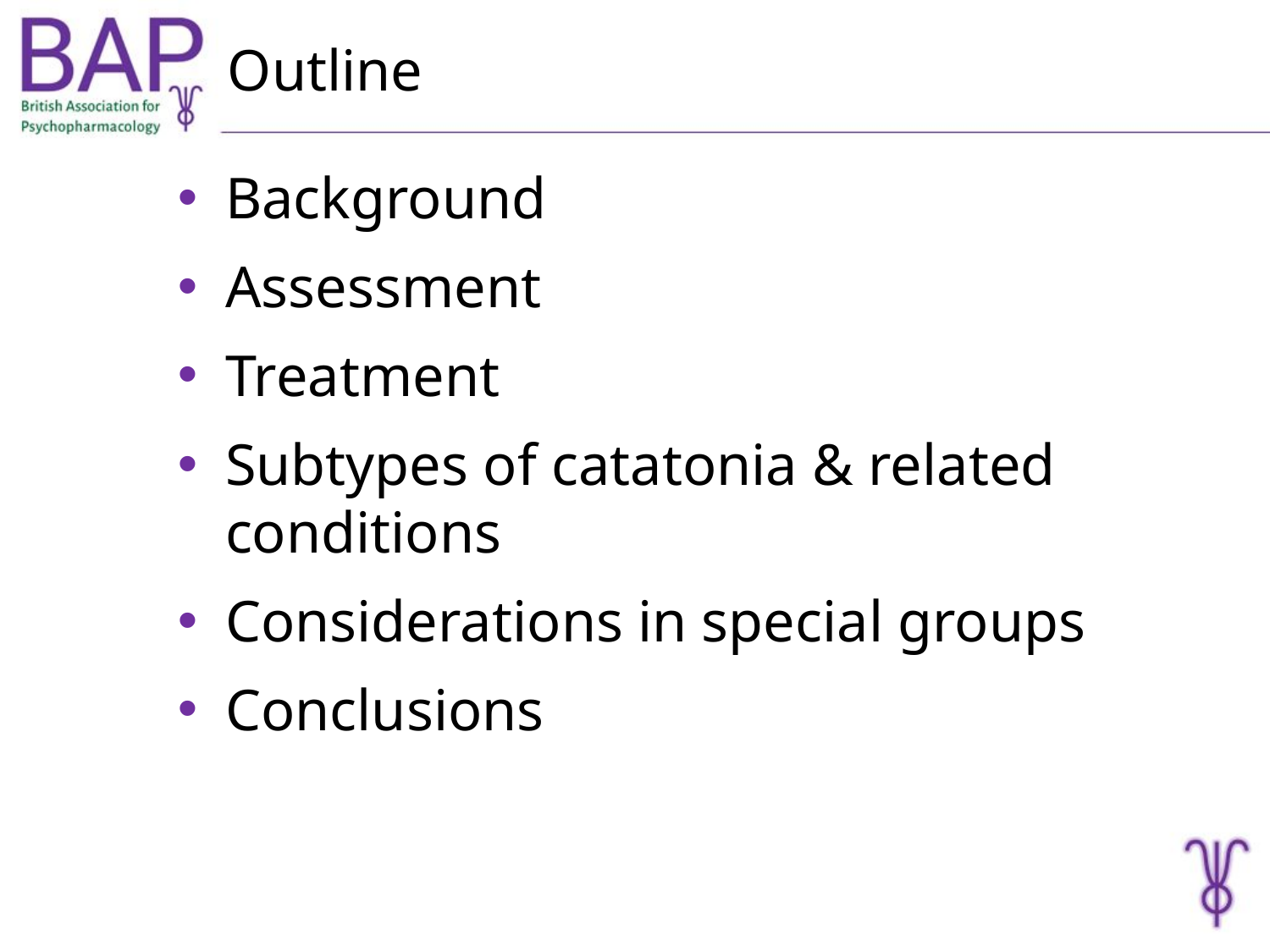

# Outline
Background
Assessment
Treatment
Subtypes of catatonia & related conditions
Considerations in special groups
Conclusions

## Slide 5
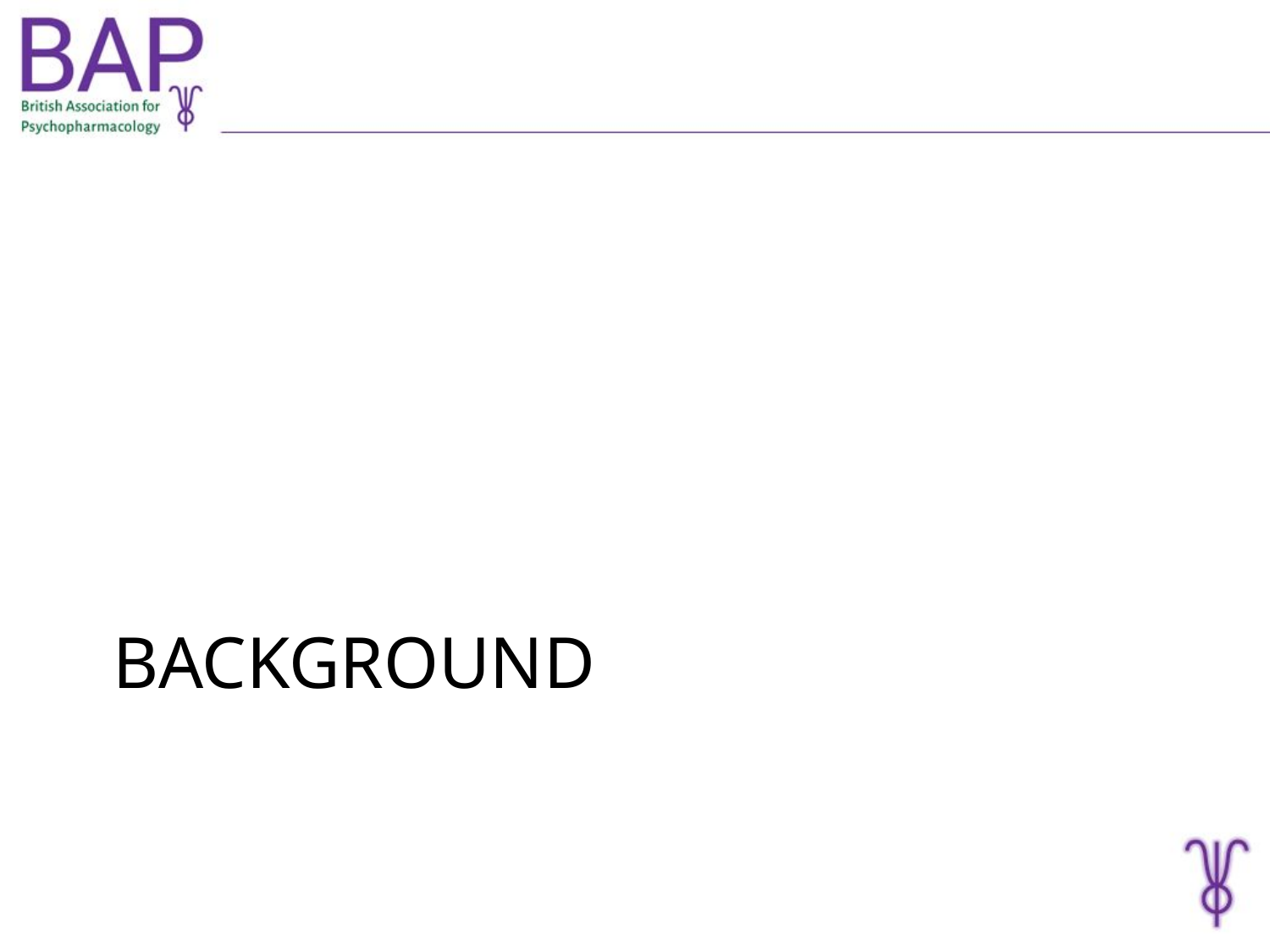

# Background

## Slide 6
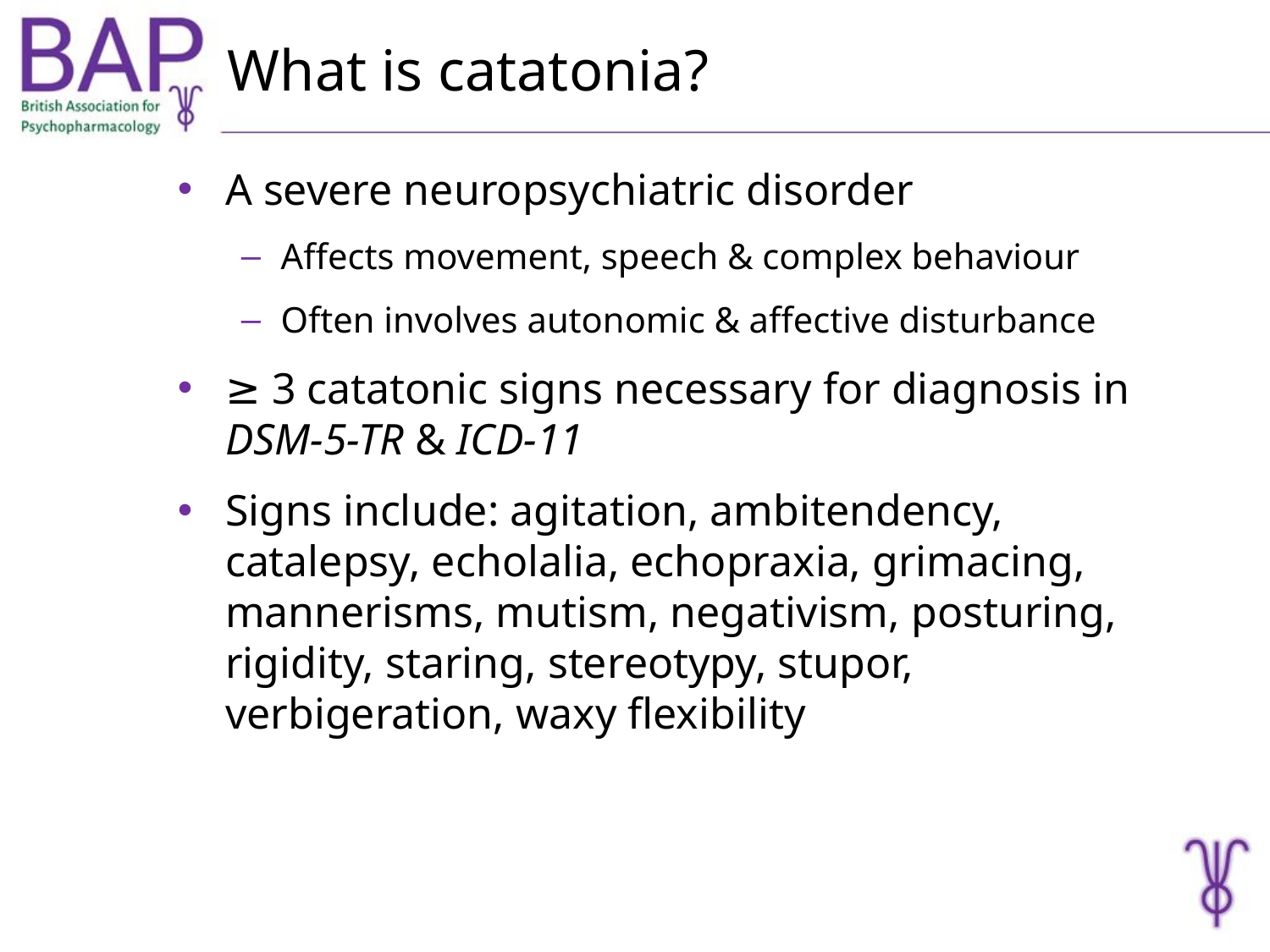

# What is catatonia?
A severe neuropsychiatric disorder
Affects movement, speech & complex behaviour
Often involves autonomic & affective disturbance
≥ 3 catatonic signs necessary for diagnosis in DSM-5-TR & ICD-11
Signs include: agitation, ambitendency, catalepsy, echolalia, echopraxia, grimacing, mannerisms, mutism, negativism, posturing, rigidity, staring, stereotypy, stupor, verbigeration, waxy flexibility

## Slide 7
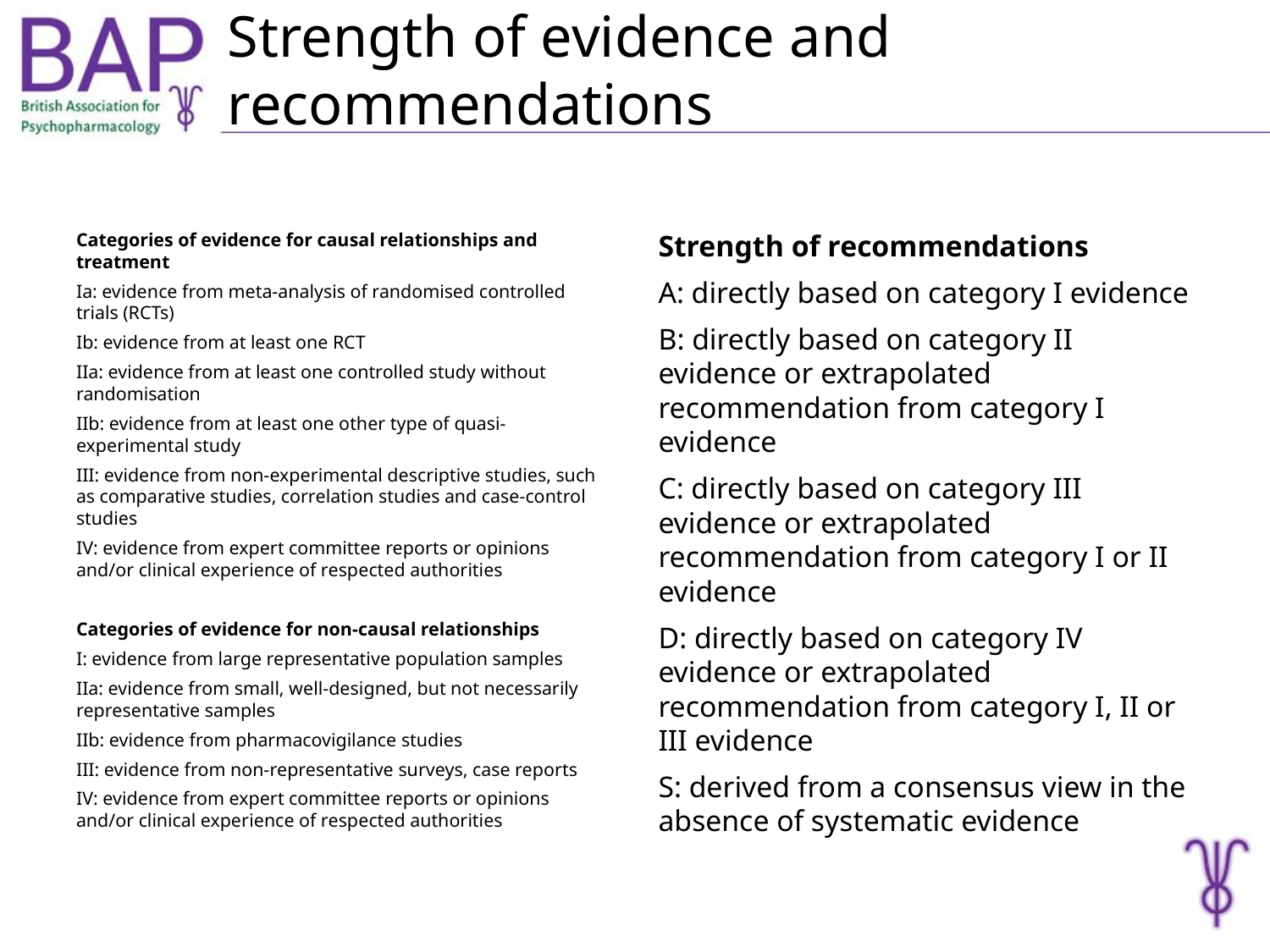

# Strength of evidence and recommendations
Categories of evidence for causal relationships and treatment
Ia: evidence from meta-analysis of randomised controlled trials (RCTs)
Ib: evidence from at least one RCT
IIa: evidence from at least one controlled study without randomisation
IIb: evidence from at least one other type of quasi-experimental study
III: evidence from non-experimental descriptive studies, such as comparative studies, correlation studies and case-control studies
IV: evidence from expert committee reports or opinions and/or clinical experience of respected authorities
Categories of evidence for non-causal relationships
I: evidence from large representative population samples
IIa: evidence from small, well-designed, but not necessarily representative samples
IIb: evidence from pharmacovigilance studies
III: evidence from non-representative surveys, case reports
IV: evidence from expert committee reports or opinions and/or clinical experience of respected authorities
Strength of recommendations
A: directly based on category I evidence
B: directly based on category II evidence or extrapolated recommendation from category I evidence
C: directly based on category III evidence or extrapolated recommendation from category I or II evidence
D: directly based on category IV evidence or extrapolated recommendation from category I, II or III evidence
S: derived from a consensus view in the absence of systematic evidence

## Slide 8
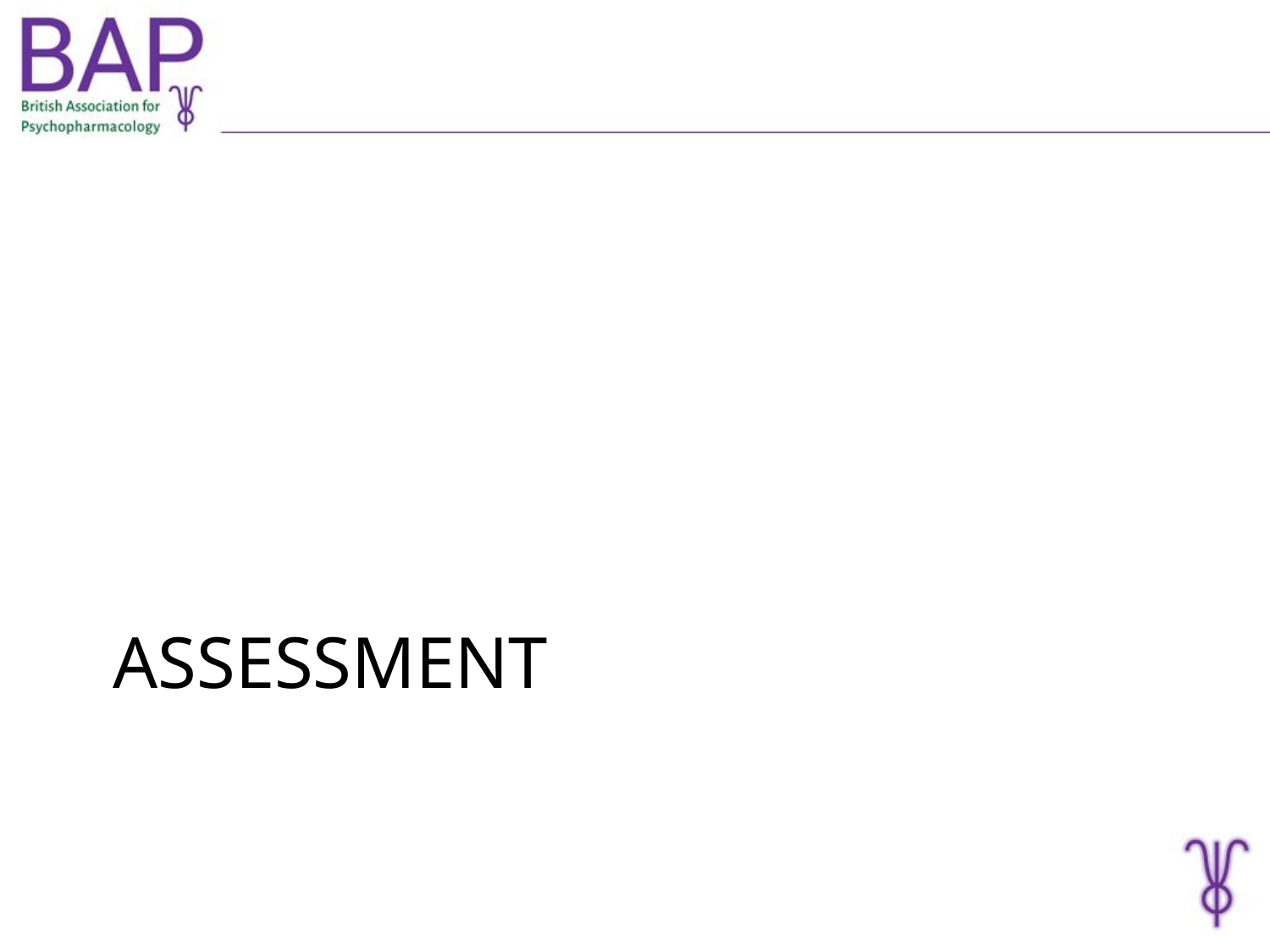

# Assessment

## Slide 9
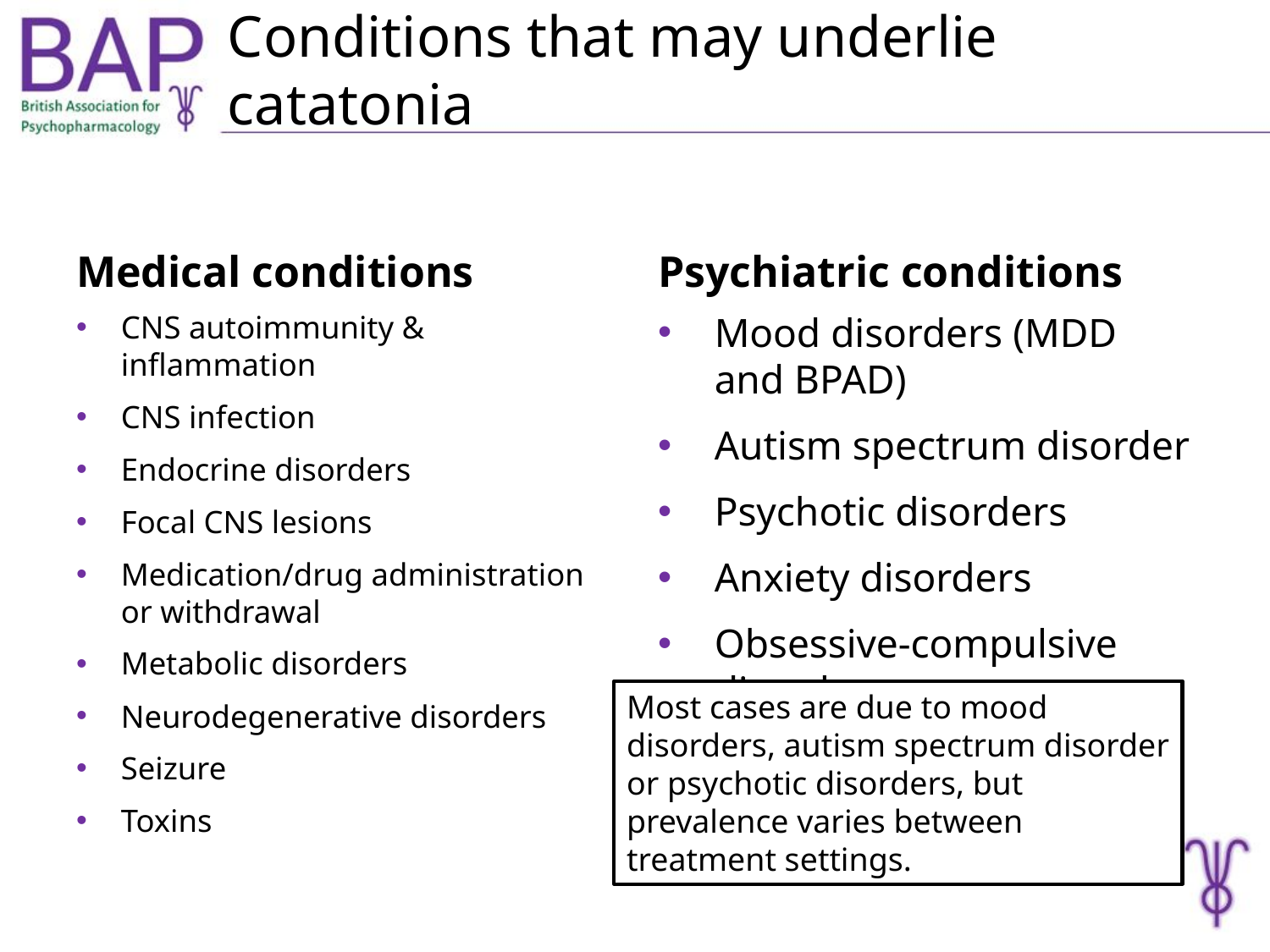

# Conditions that may underlie catatonia
Medical conditions
Psychiatric conditions
CNS autoimmunity & inflammation
CNS infection
Endocrine disorders
Focal CNS lesions
Medication/drug administration or withdrawal
Metabolic disorders
Neurodegenerative disorders
Seizure
Toxins
Mood disorders (MDD and BPAD)
Autism spectrum disorder
Psychotic disorders
Anxiety disorders
Obsessive-compulsive disorder
Post-traumatic stress disorder (PTSD)
Most cases are due to mood disorders, autism spectrum disorder or psychotic disorders, but prevalence varies between treatment settings.

## Slide 10
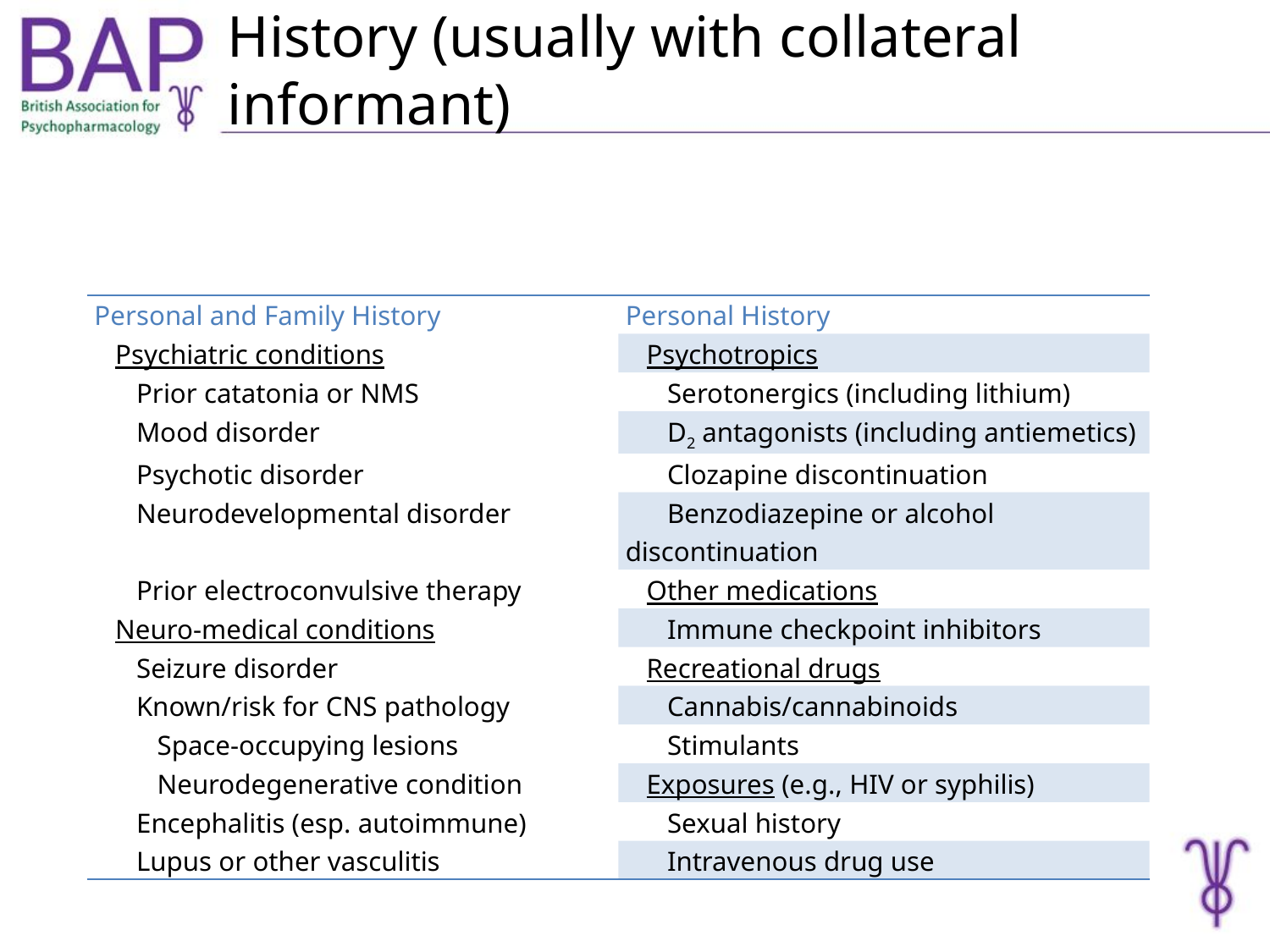

# History (usually with collateral informant)
| Personal and Family History | Personal History |
| --- | --- |
| Psychiatric conditions | Psychotropics |
| Prior catatonia or NMS | Serotonergics (including lithium) |
| Mood disorder | D2 antagonists (including antiemetics) |
| Psychotic disorder | Clozapine discontinuation |
| Neurodevelopmental disorder | Benzodiazepine or alcohol discontinuation |
| Prior electroconvulsive therapy | Other medications |
| Neuro-medical conditions | Immune checkpoint inhibitors |
| Seizure disorder | Recreational drugs |
| Known/risk for CNS pathology | Cannabis/cannabinoids |
| Space-occupying lesions | Stimulants |
| Neurodegenerative condition | Exposures (e.g., HIV or syphilis) |
| Encephalitis (esp. autoimmune) | Sexual history |
| Lupus or other vasculitis | Intravenous drug use |

## Slide 11
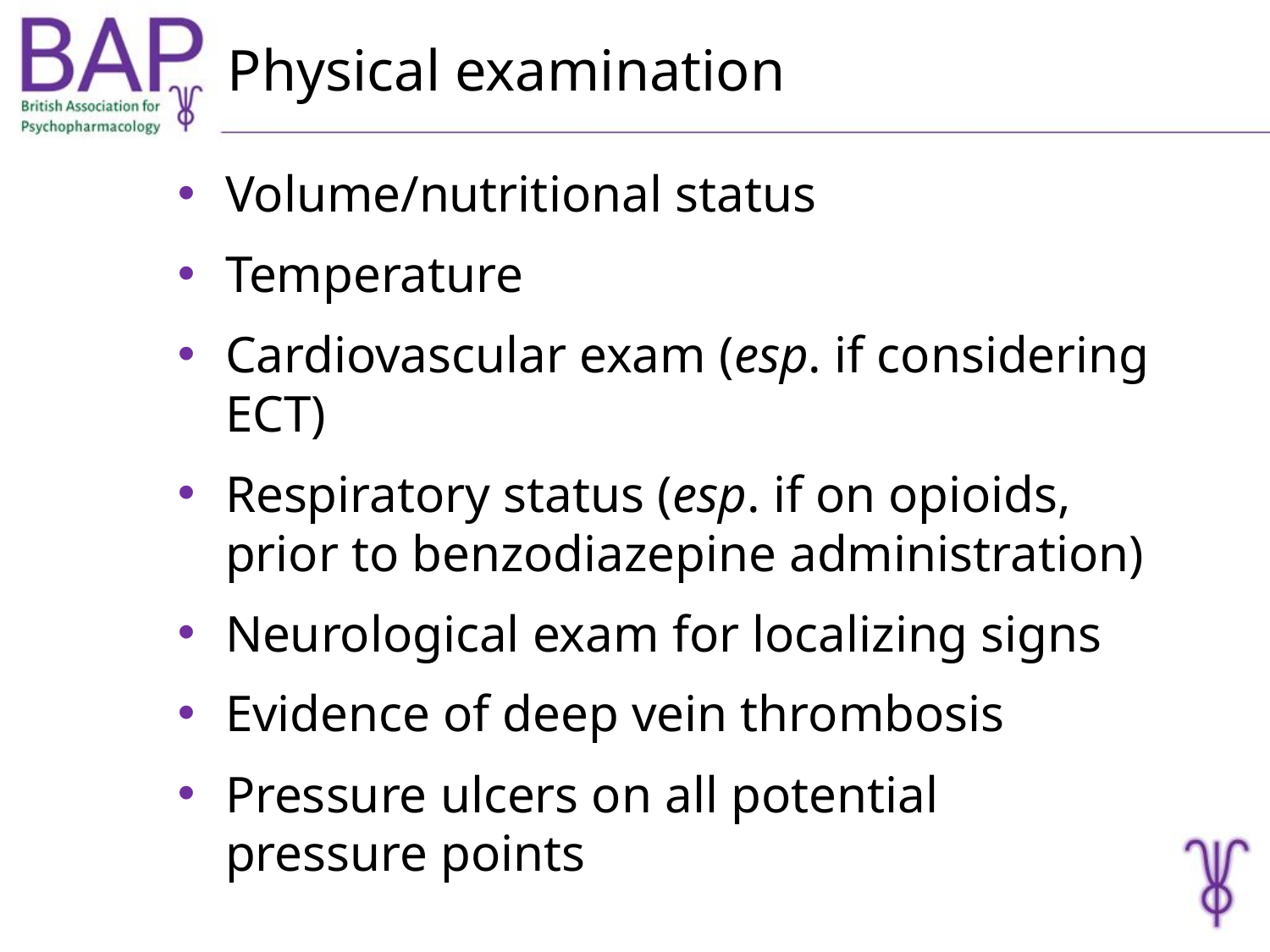

# Physical examination
Volume/nutritional status
Temperature
Cardiovascular exam (esp. if considering ECT)
Respiratory status (esp. if on opioids, prior to benzodiazepine administration)
Neurological exam for localizing signs
Evidence of deep vein thrombosis
Pressure ulcers on all potential pressure points

## Slide 12
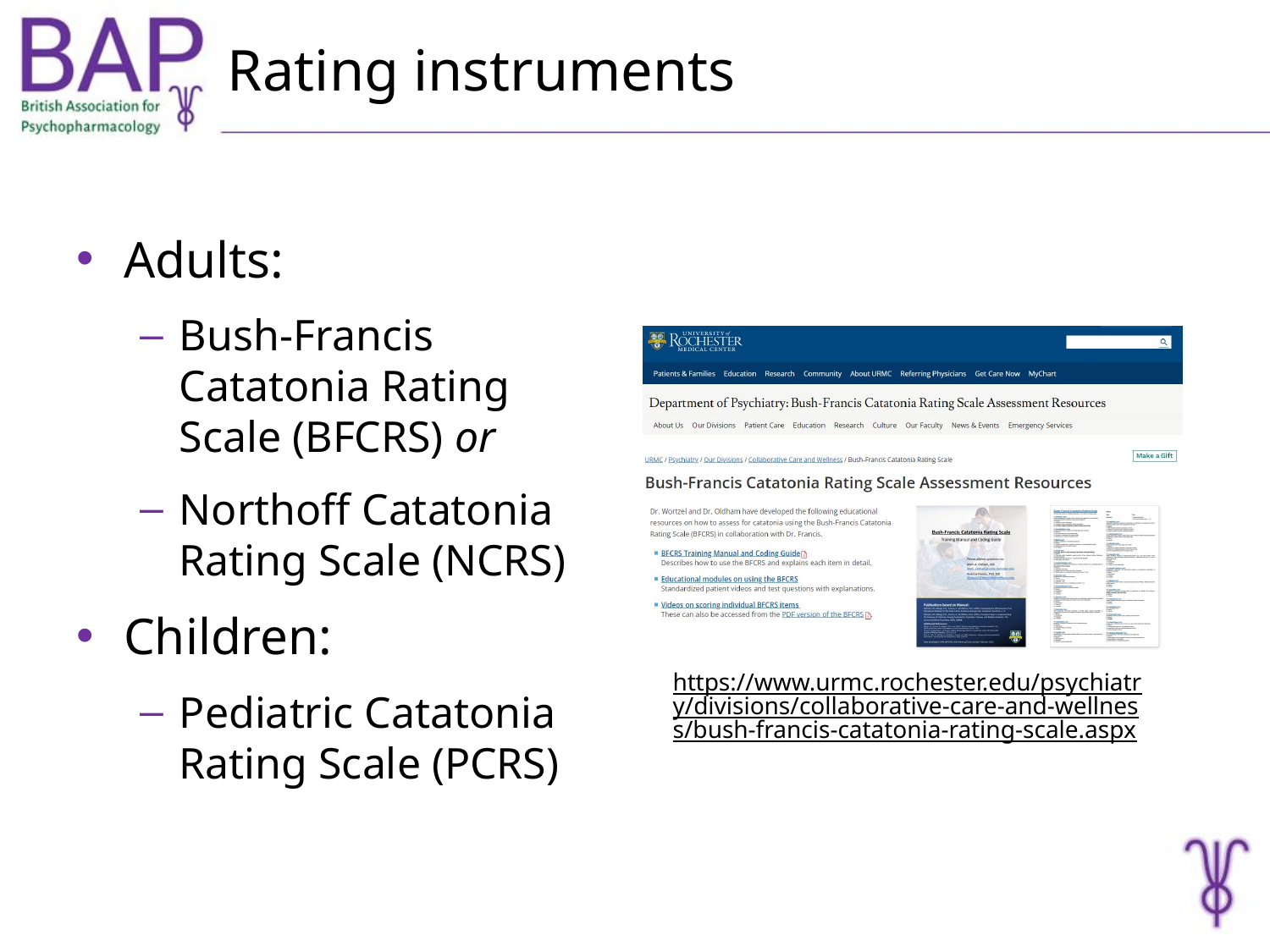

# Rating instruments
Adults:
Bush-Francis Catatonia Rating Scale (BFCRS) or
Northoff Catatonia Rating Scale (NCRS)
Children:
Pediatric Catatonia Rating Scale (PCRS)
https://www.urmc.rochester.edu/psychiatry/divisions/collaborative-care-and-wellness/bush-francis-catatonia-rating-scale.aspx

## Slide 13
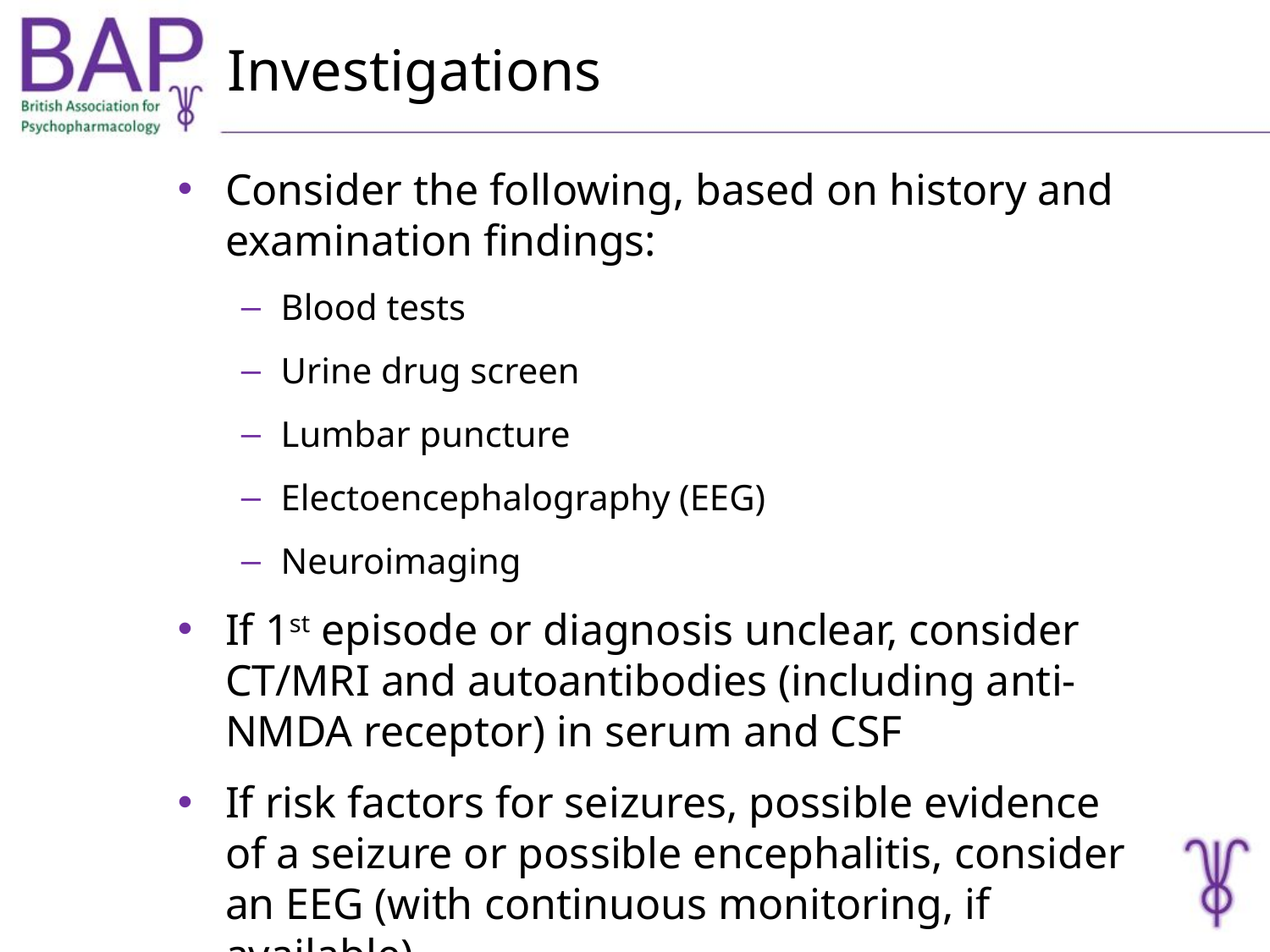

# Investigations
Consider the following, based on history and examination findings:
Blood tests
Urine drug screen
Lumbar puncture
Electoencephalography (EEG)
Neuroimaging
If 1st episode or diagnosis unclear, consider CT/MRI and autoantibodies (including anti-NMDA receptor) in serum and CSF
If risk factors for seizures, possible evidence of a seizure or possible encephalitis, consider an EEG (with continuous monitoring, if available)

## Slide 14
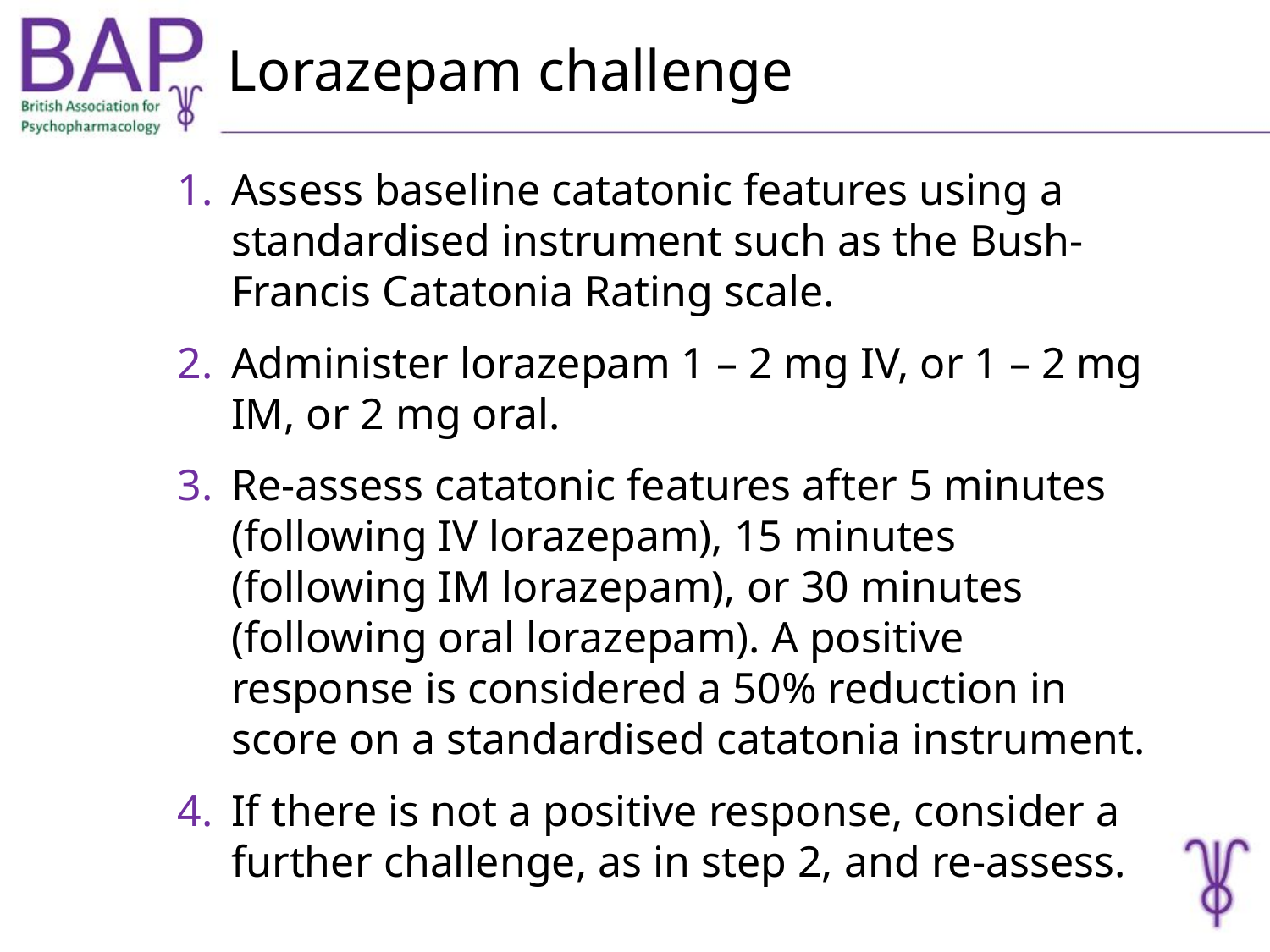

# Lorazepam challenge
Assess baseline catatonic features using a standardised instrument such as the Bush-Francis Catatonia Rating scale.
Administer lorazepam 1 – 2 mg IV, or 1 – 2 mg IM, or 2 mg oral.
Re-assess catatonic features after 5 minutes (following IV lorazepam), 15 minutes (following IM lorazepam), or 30 minutes (following oral lorazepam). A positive response is considered a 50% reduction in score on a standardised catatonia instrument.
If there is not a positive response, consider a further challenge, as in step 2, and re-assess.

## Slide 15
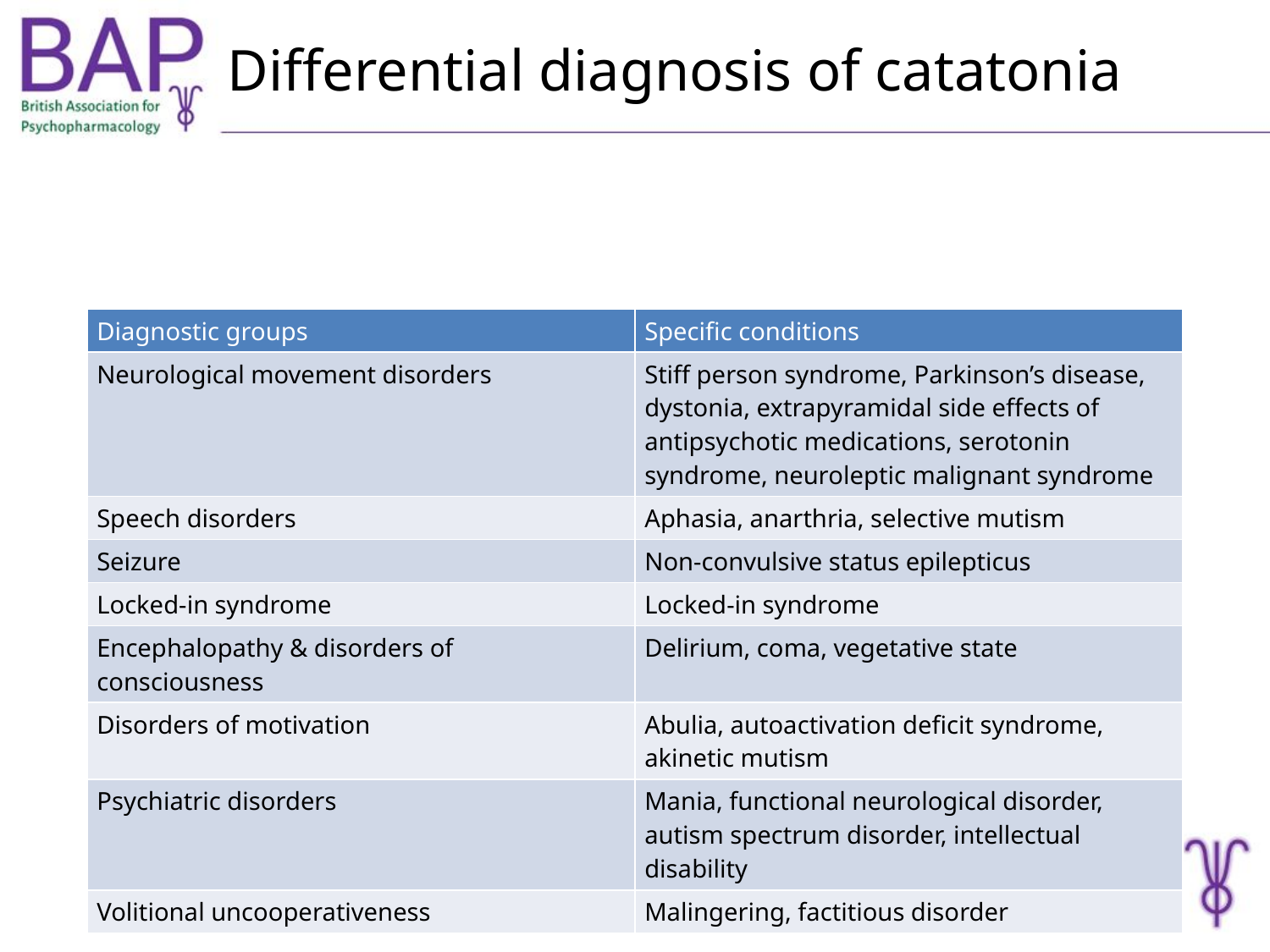

# Differential diagnosis of catatonia
| Diagnostic groups | Specific conditions |
| --- | --- |
| Neurological movement disorders | Stiff person syndrome, Parkinson’s disease, dystonia, extrapyramidal side effects of antipsychotic medications, serotonin syndrome, neuroleptic malignant syndrome |
| Speech disorders | Aphasia, anarthria, selective mutism |
| Seizure | Non-convulsive status epilepticus |
| Locked-in syndrome | Locked-in syndrome |
| Encephalopathy & disorders of consciousness | Delirium, coma, vegetative state |
| Disorders of motivation | Abulia, autoactivation deficit syndrome, akinetic mutism |
| Psychiatric disorders | Mania, functional neurological disorder, autism spectrum disorder, intellectual disability |
| Volitional uncooperativeness | Malingering, factitious disorder |

## Slide 16
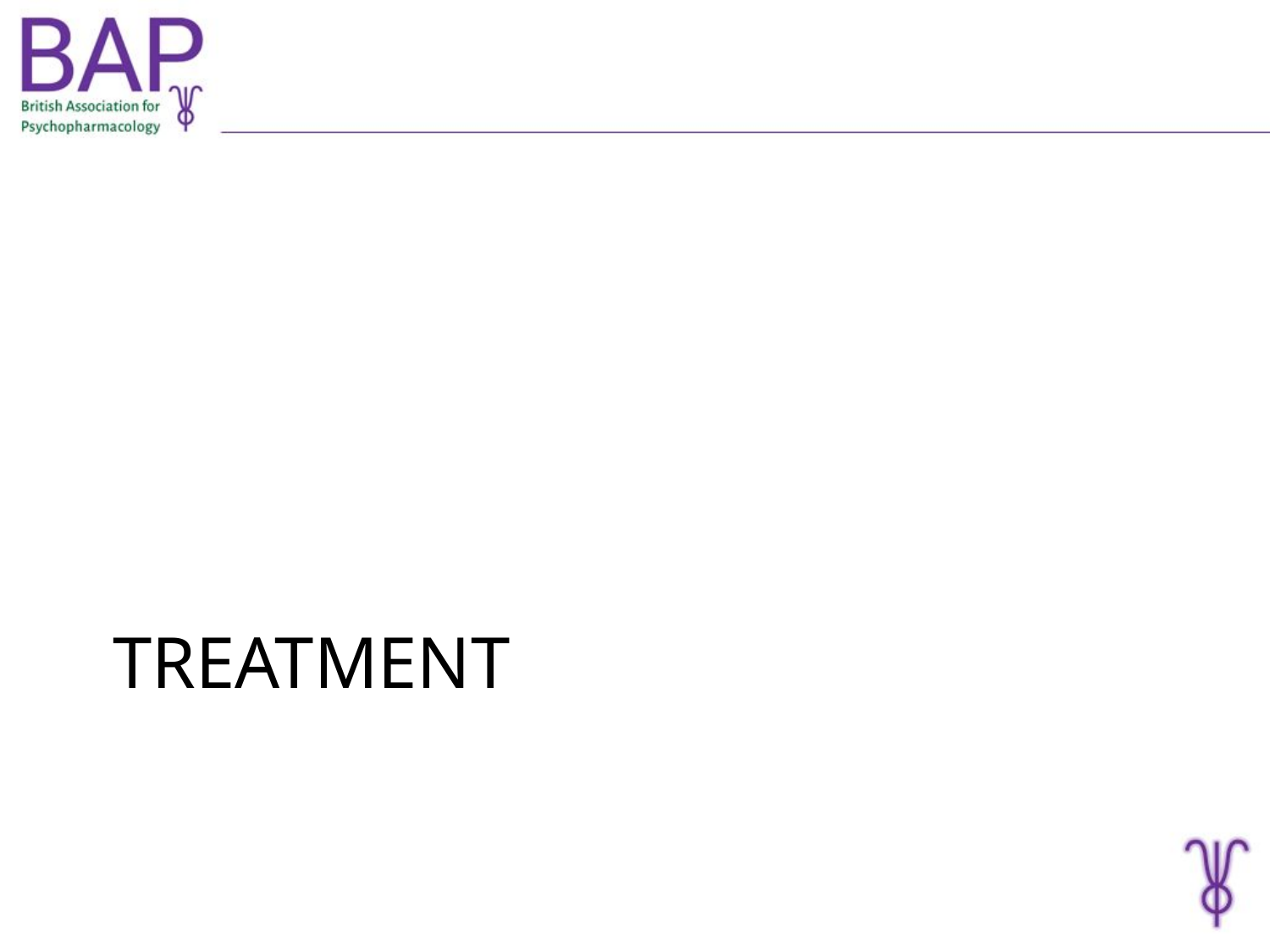

# Treatment

## Slide 17
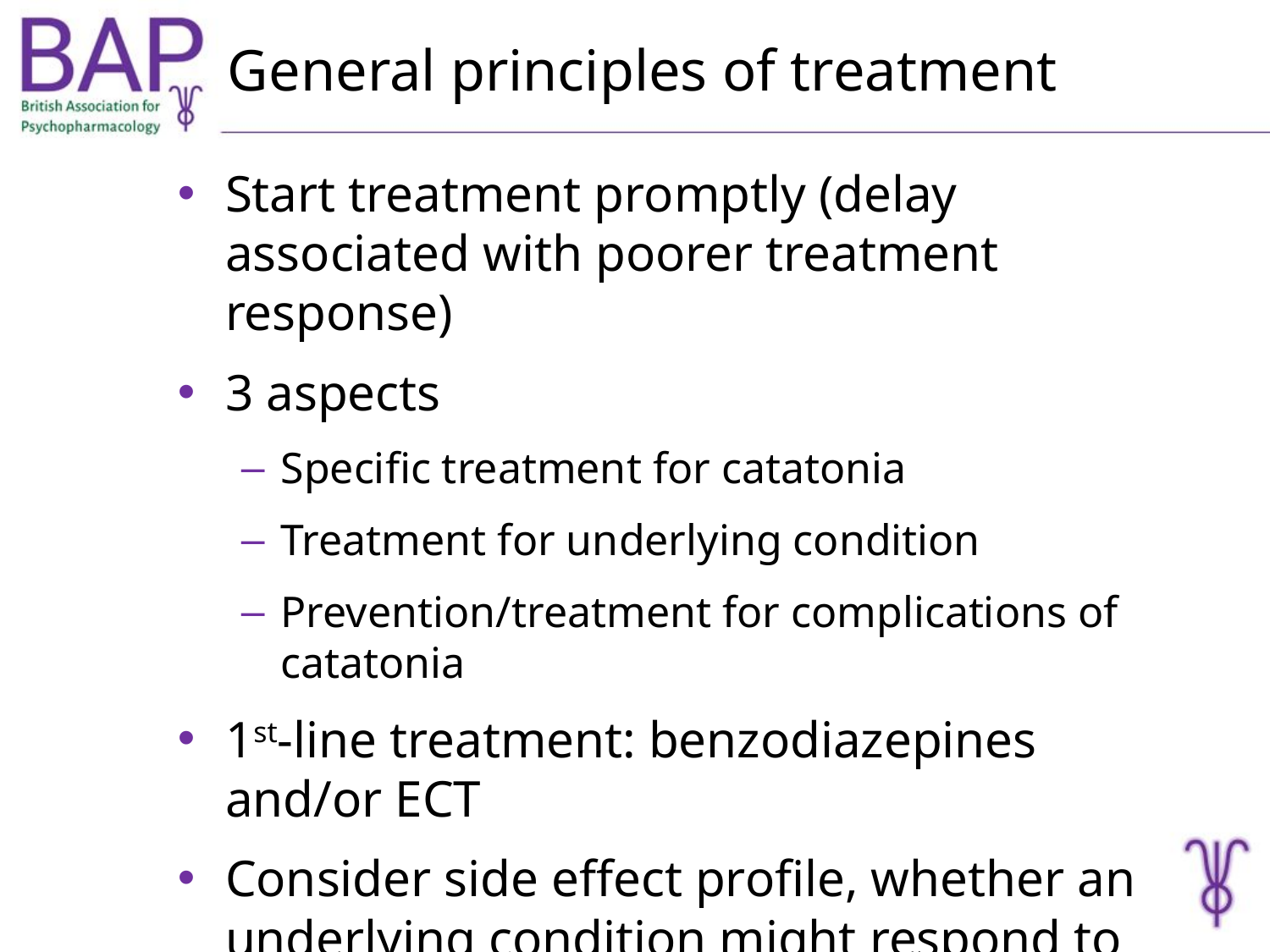

# General principles of treatment
Start treatment promptly (delay associated with poorer treatment response)
3 aspects
Specific treatment for catatonia
Treatment for underlying condition
Prevention/treatment for complications of catatonia
1st-line treatment: benzodiazepines and/or ECT
Consider side effect profile, whether an underlying condition might respond to ECT (e.g. depression) and local availability

## Slide 18
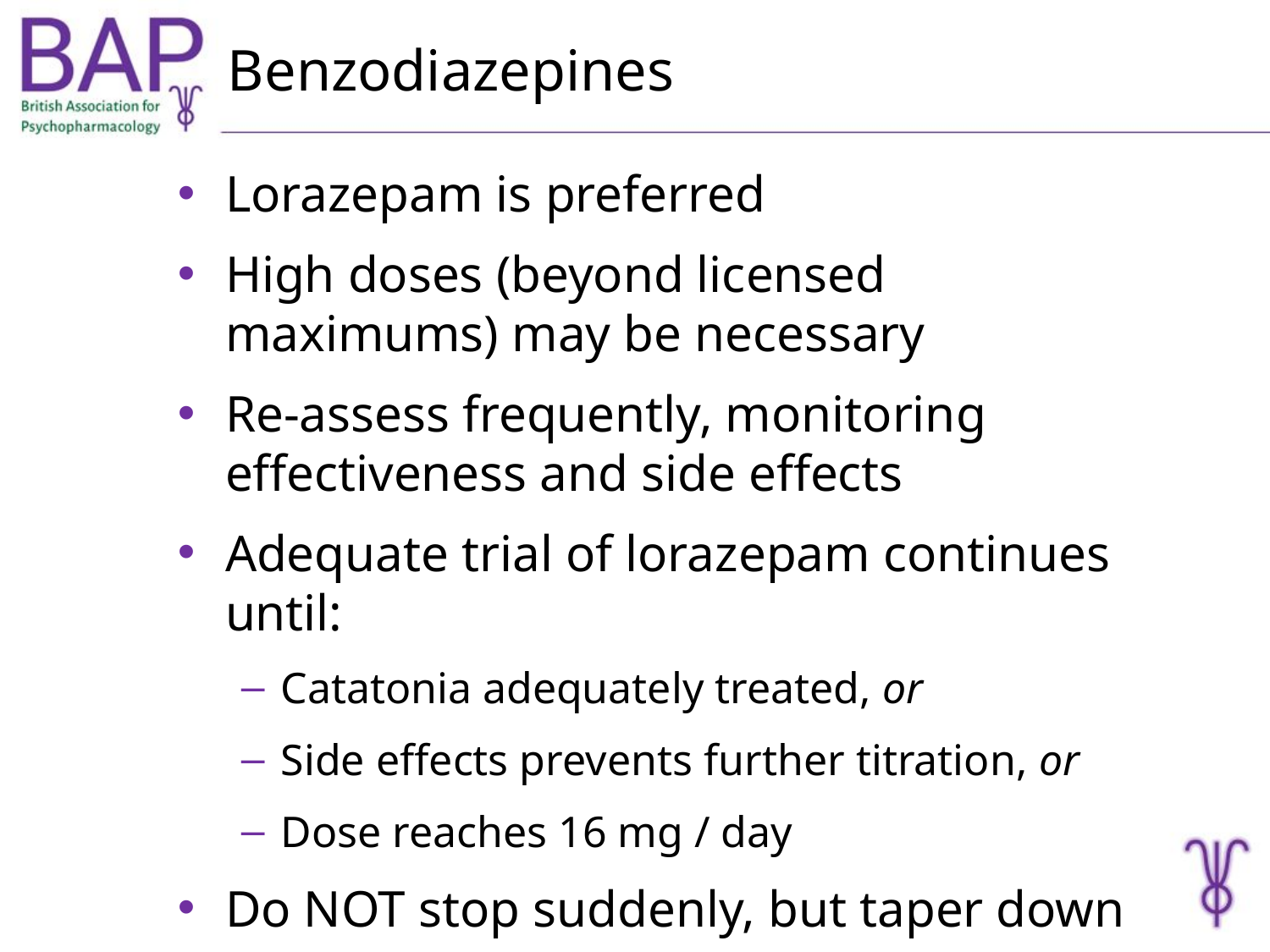

# Benzodiazepines
Lorazepam is preferred
High doses (beyond licensed maximums) may be necessary
Re-assess frequently, monitoring effectiveness and side effects
Adequate trial of lorazepam continues until:
Catatonia adequately treated, or
Side effects prevents further titration, or
Dose reaches 16 mg / day
Do NOT stop suddenly, but taper down

## Slide 19
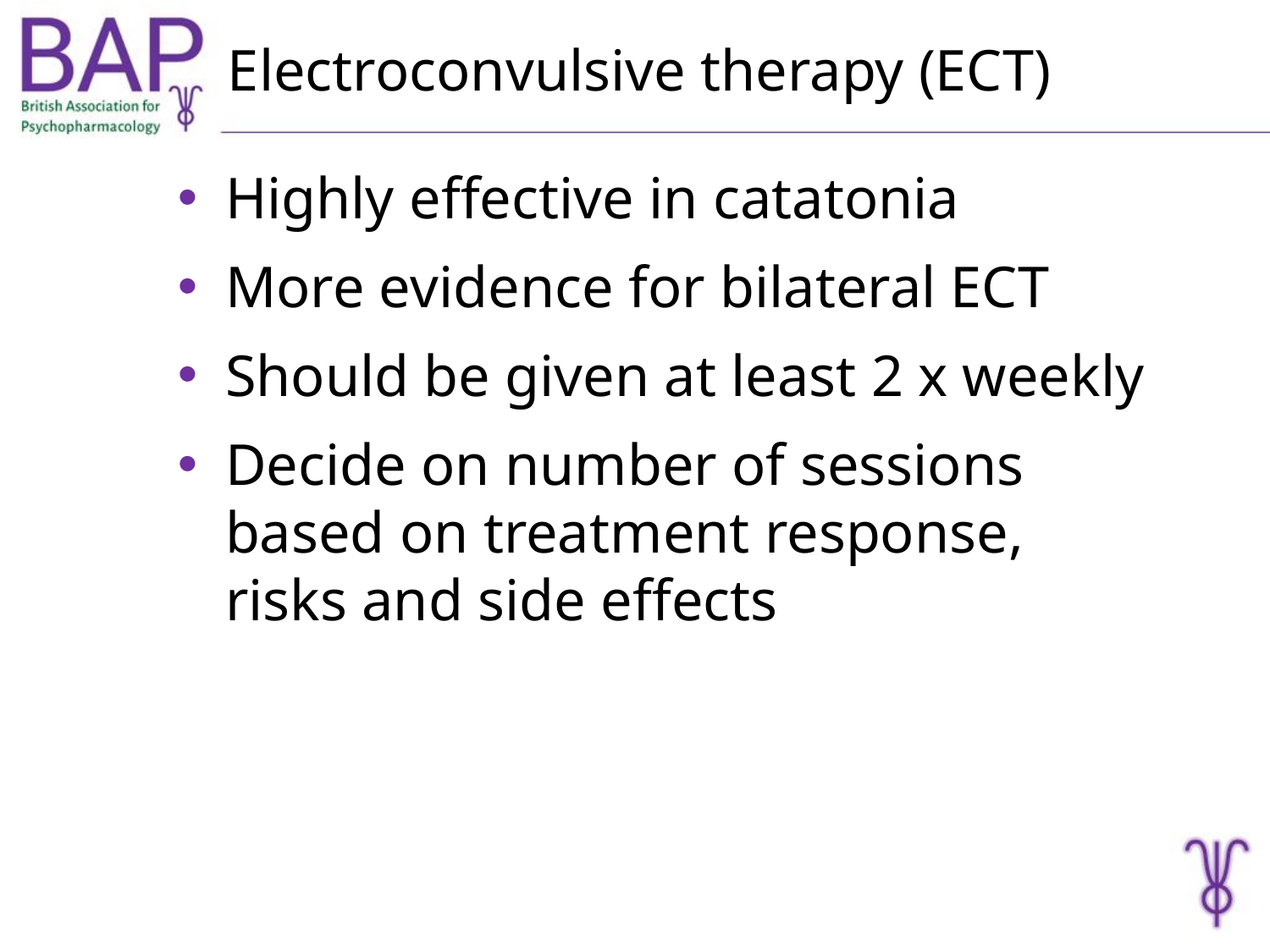

# Electroconvulsive therapy (ECT)
Highly effective in catatonia
More evidence for bilateral ECT
Should be given at least 2 x weekly
Decide on number of sessions based on treatment response, risks and side effects

## Slide 20
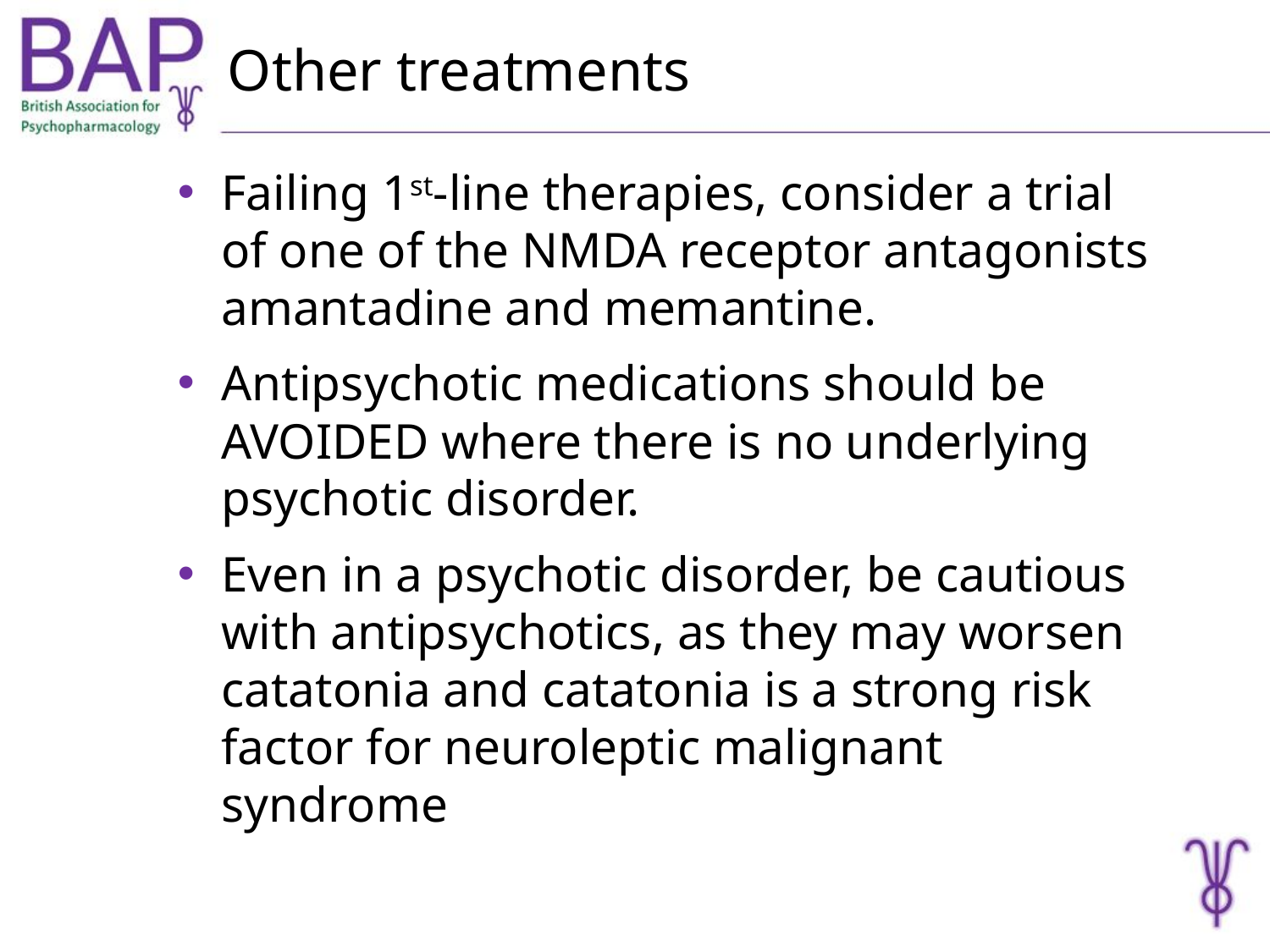

# Other treatments
Failing 1st-line therapies, consider a trial of one of the NMDA receptor antagonists amantadine and memantine.
Antipsychotic medications should be AVOIDED where there is no underlying psychotic disorder.
Even in a psychotic disorder, be cautious with antipsychotics, as they may worsen catatonia and catatonia is a strong risk factor for neuroleptic malignant syndrome

## Slide 21
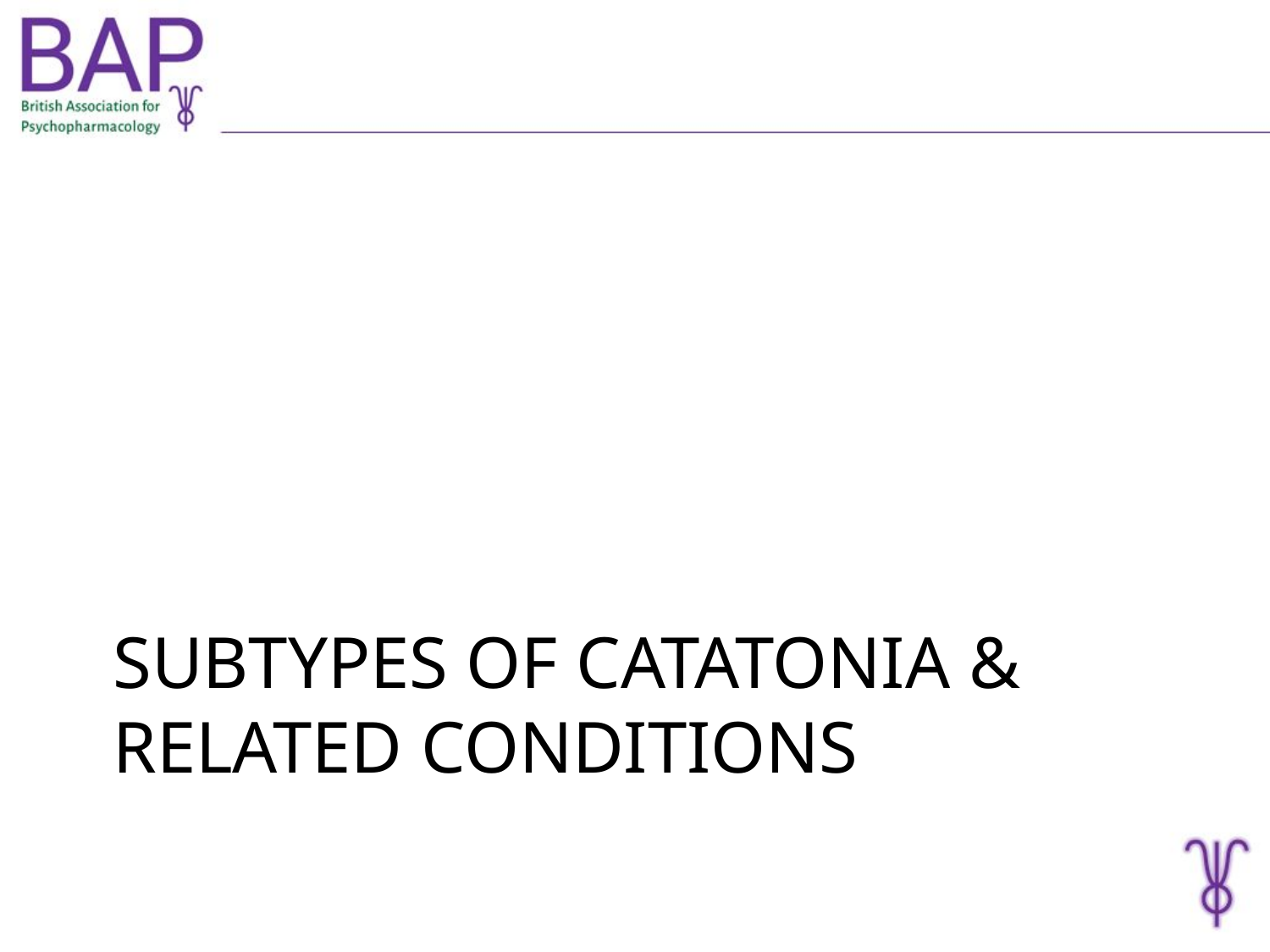

# Subtypes of catatonia & related conditions

## Slide 22
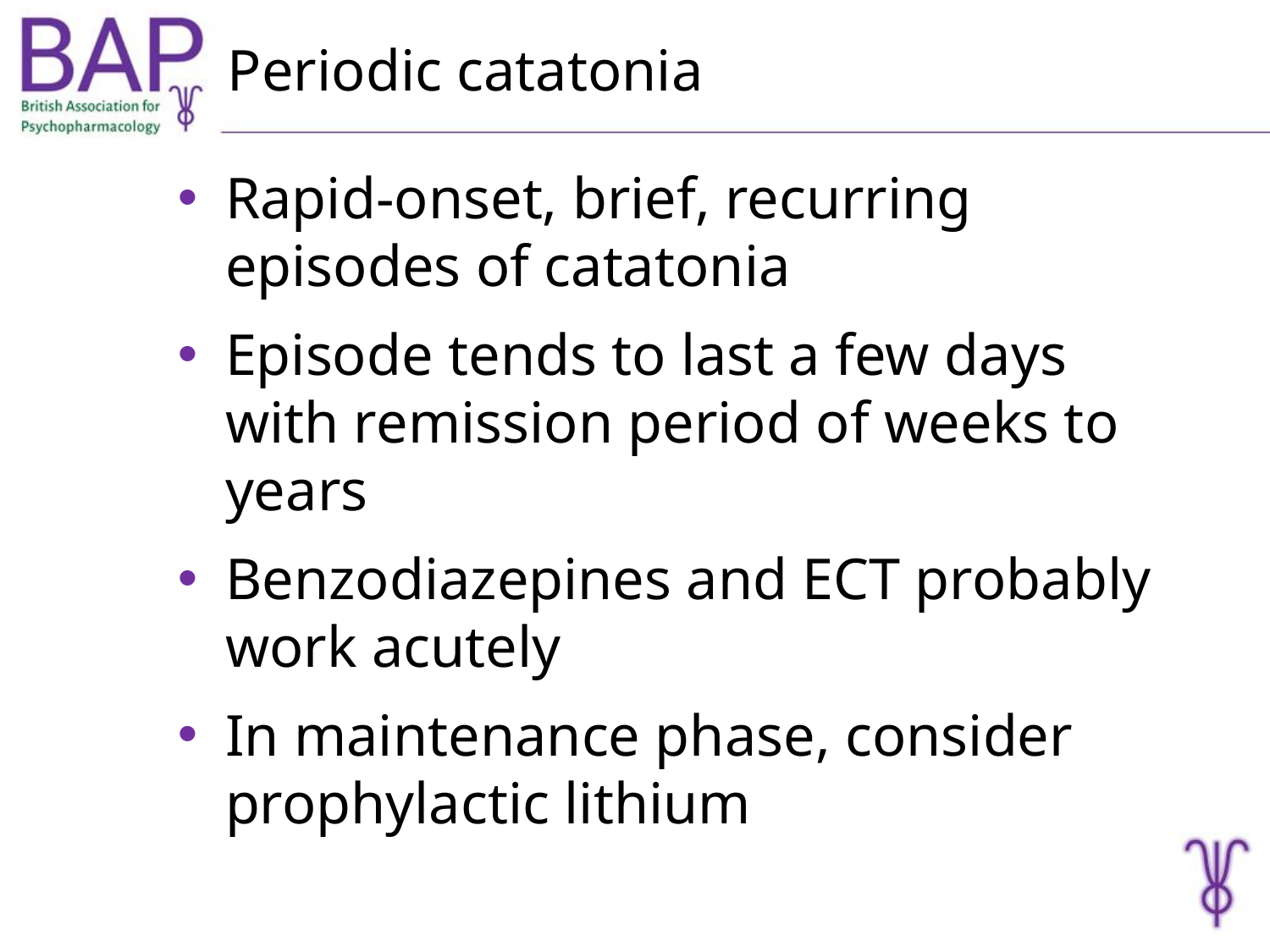

# Periodic catatonia
Rapid-onset, brief, recurring episodes of catatonia
Episode tends to last a few days with remission period of weeks to years
Benzodiazepines and ECT probably work acutely
In maintenance phase, consider prophylactic lithium

## Slide 23
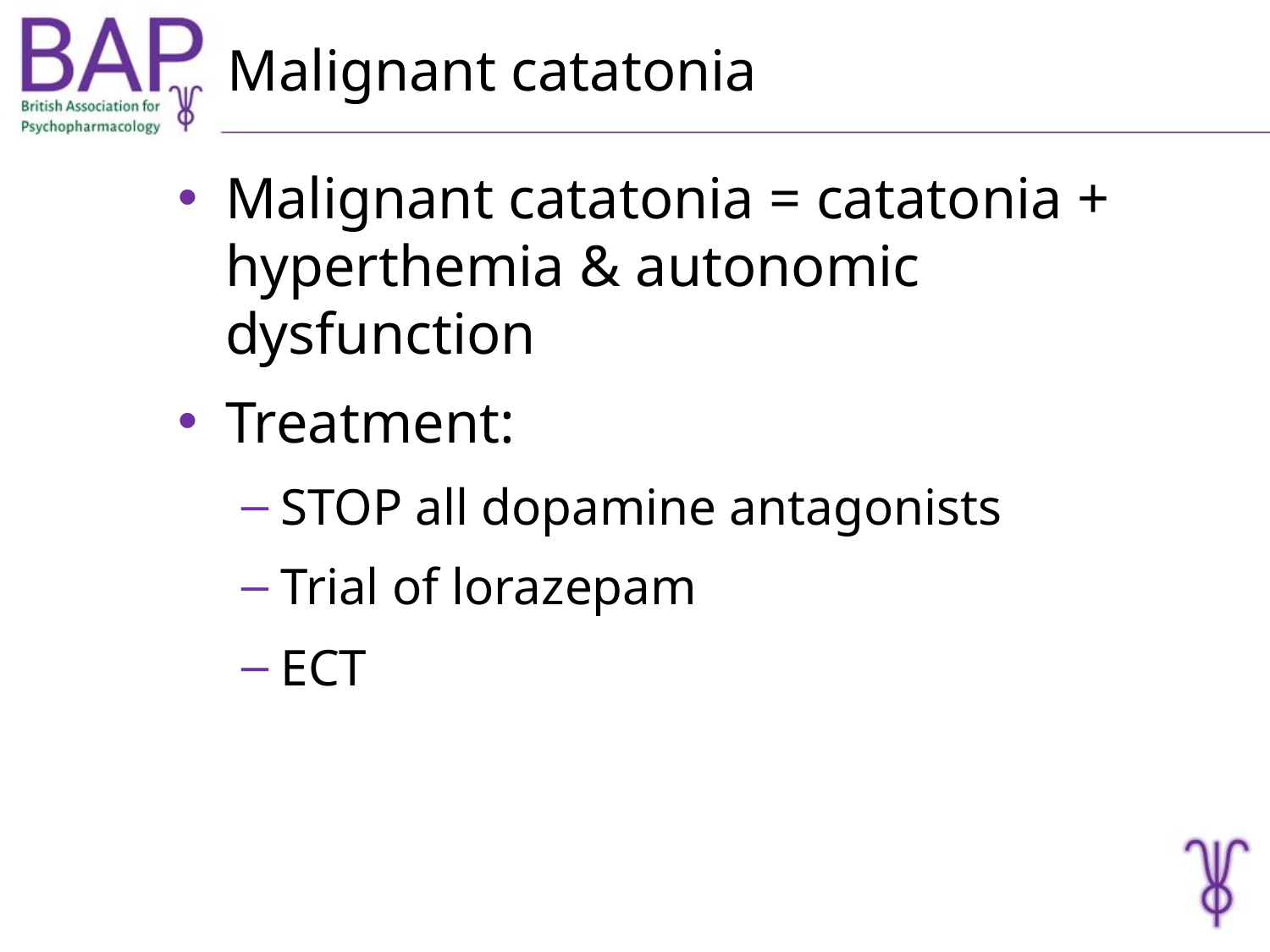

# Malignant catatonia
Malignant catatonia = catatonia + hyperthemia & autonomic dysfunction
Treatment:
STOP all dopamine antagonists
Trial of lorazepam
ECT

## Slide 24
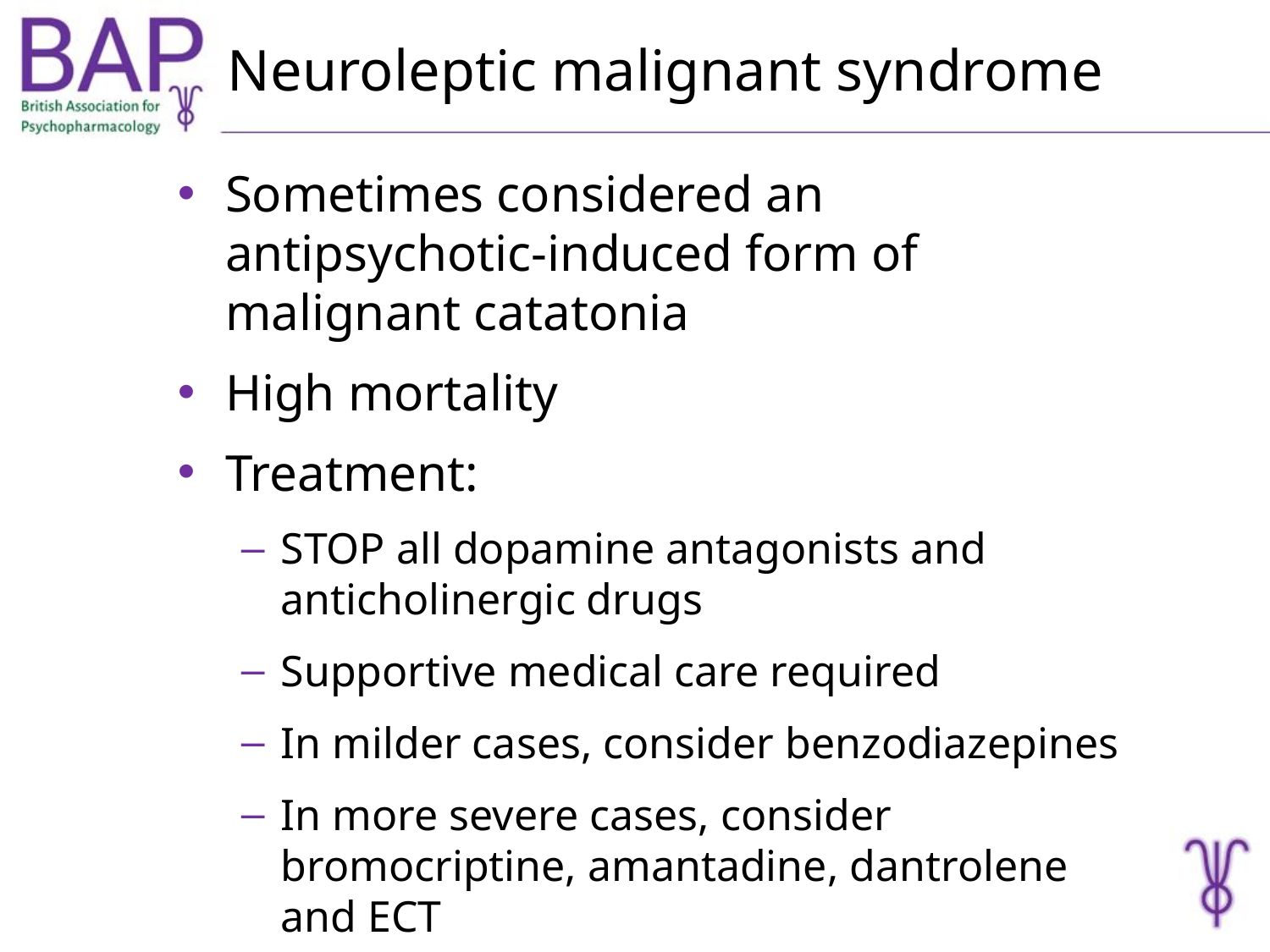

# Neuroleptic malignant syndrome
Sometimes considered an antipsychotic-induced form of malignant catatonia
High mortality
Treatment:
STOP all dopamine antagonists and anticholinergic drugs
Supportive medical care required
In milder cases, consider benzodiazepines
In more severe cases, consider bromocriptine, amantadine, dantrolene and ECT

## Slide 25
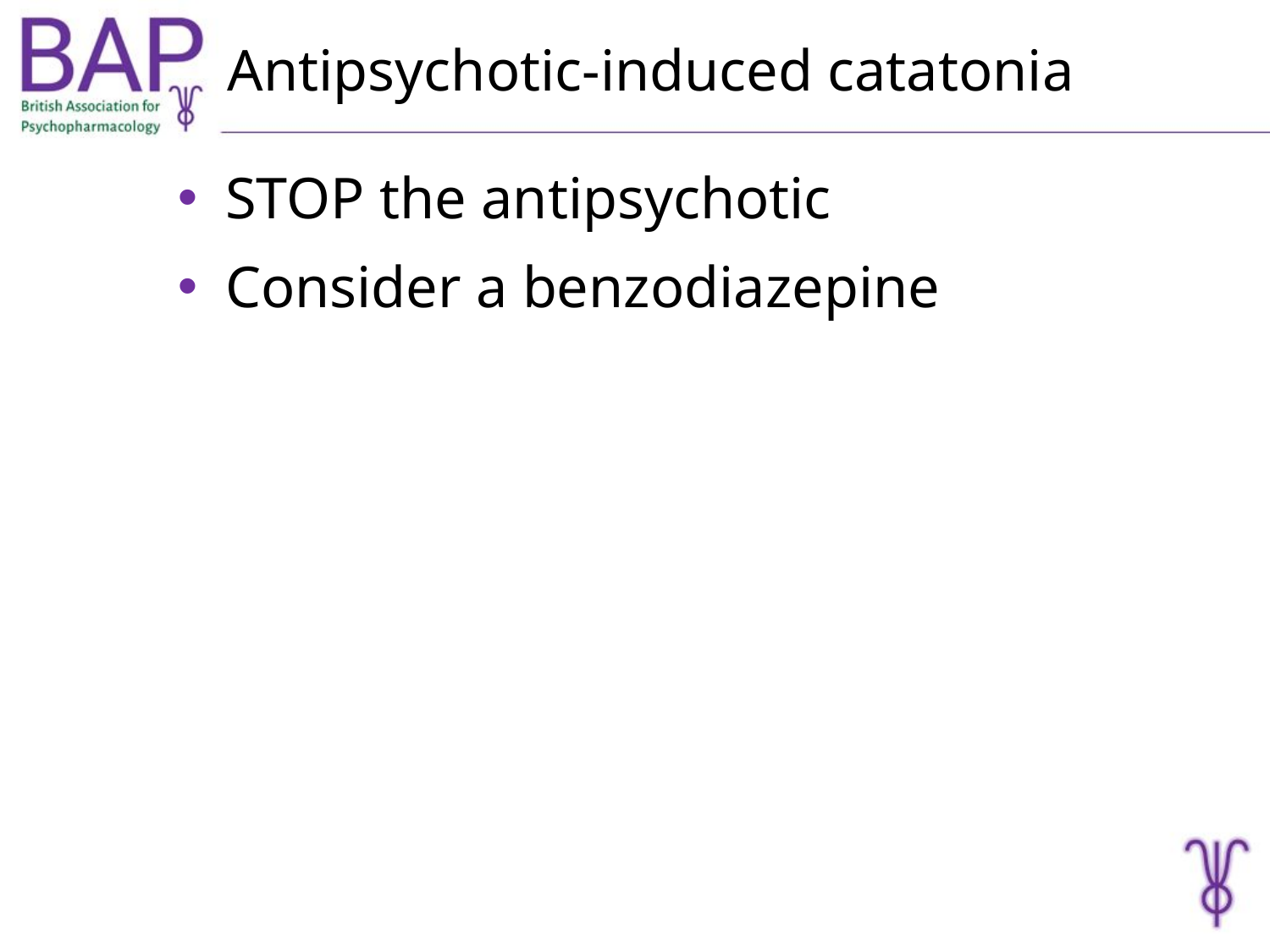

# Antipsychotic-induced catatonia
STOP the antipsychotic
Consider a benzodiazepine

## Slide 26
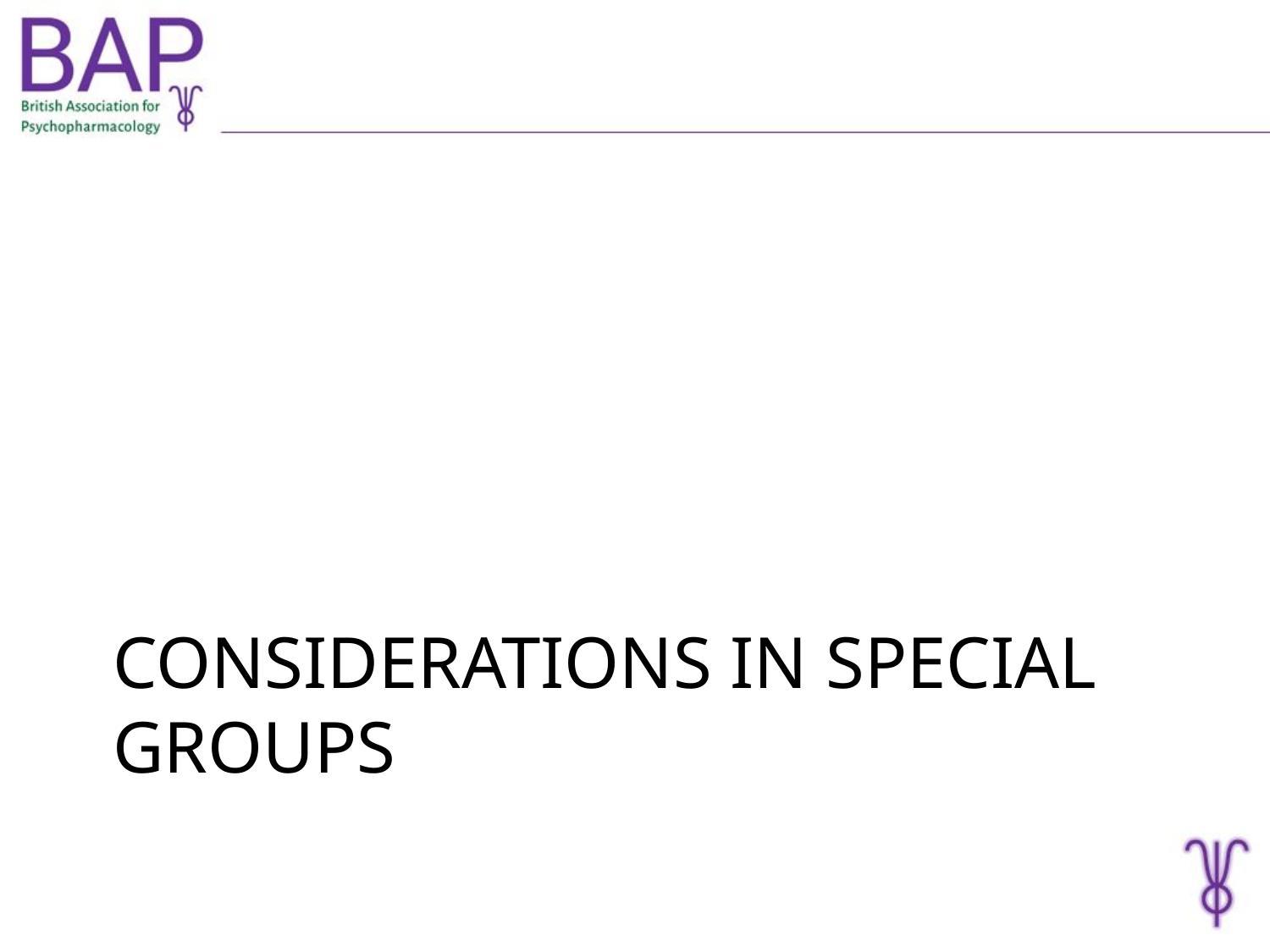

# Considerations in special groups

## Slide 27
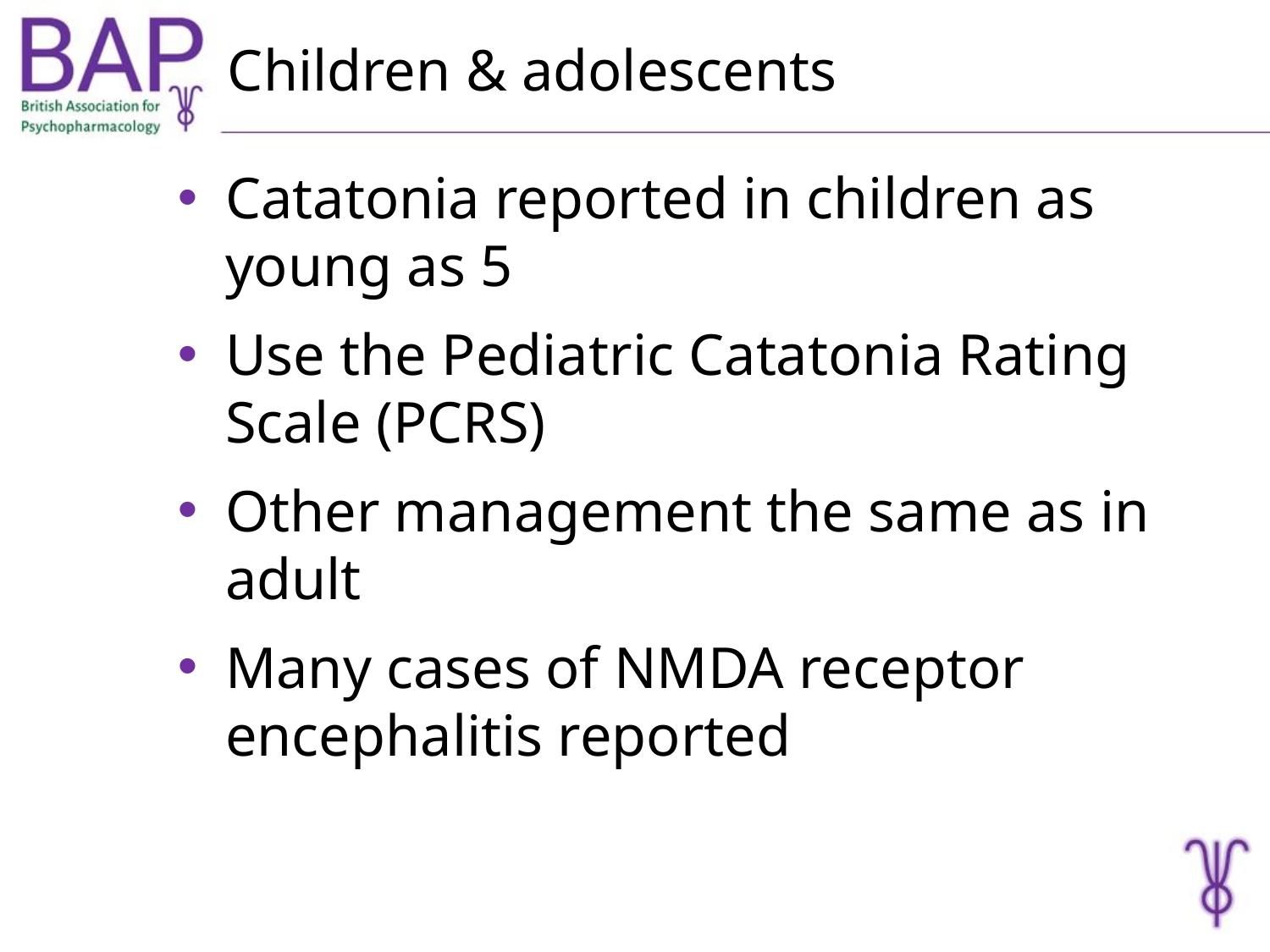

# Children & adolescents
Catatonia reported in children as young as 5
Use the Pediatric Catatonia Rating Scale (PCRS)
Other management the same as in adult
Many cases of NMDA receptor encephalitis reported

## Slide 28
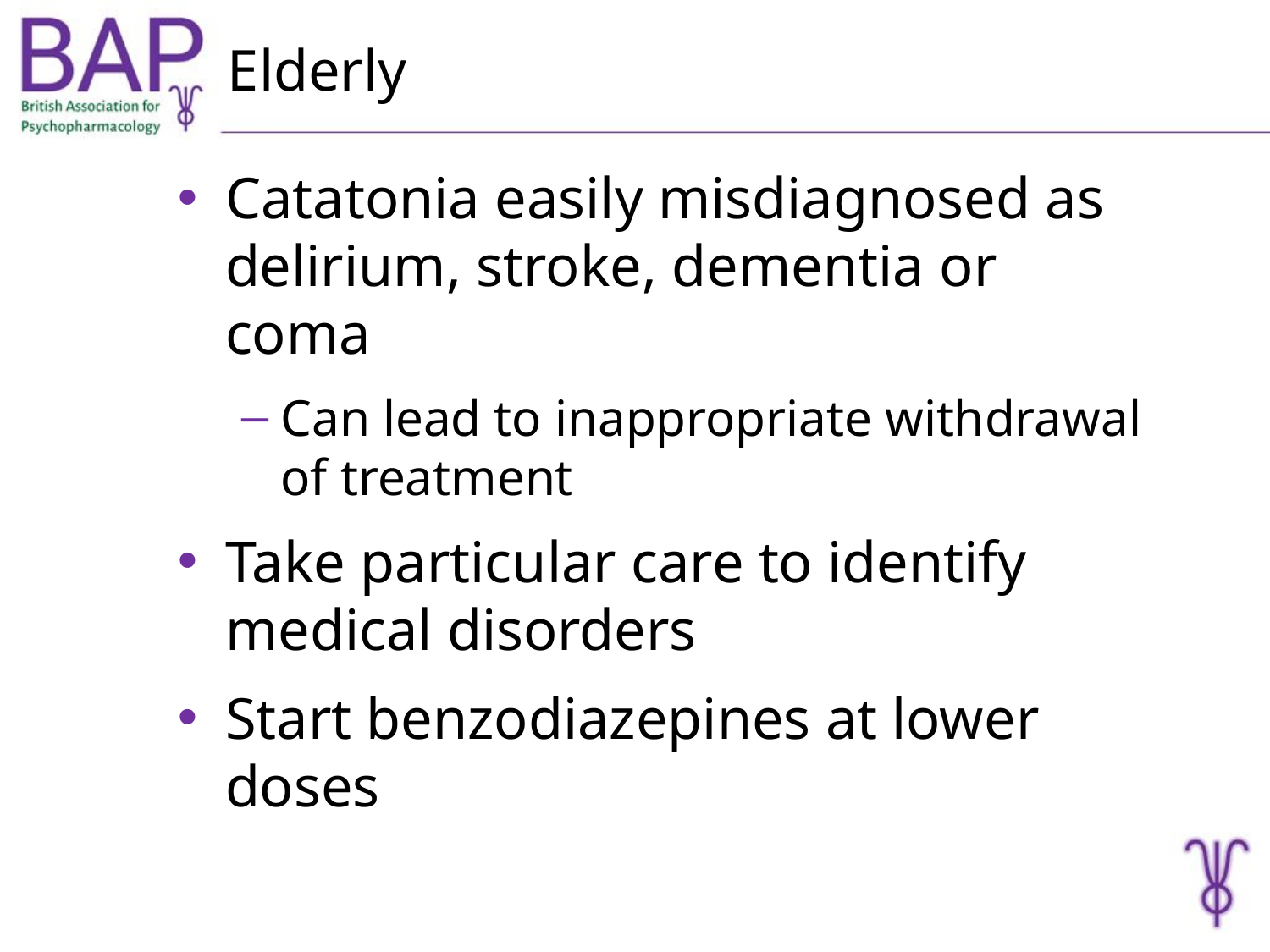

# Elderly
Catatonia easily misdiagnosed as delirium, stroke, dementia or coma
Can lead to inappropriate withdrawal of treatment
Take particular care to identify medical disorders
Start benzodiazepines at lower doses

## Slide 29
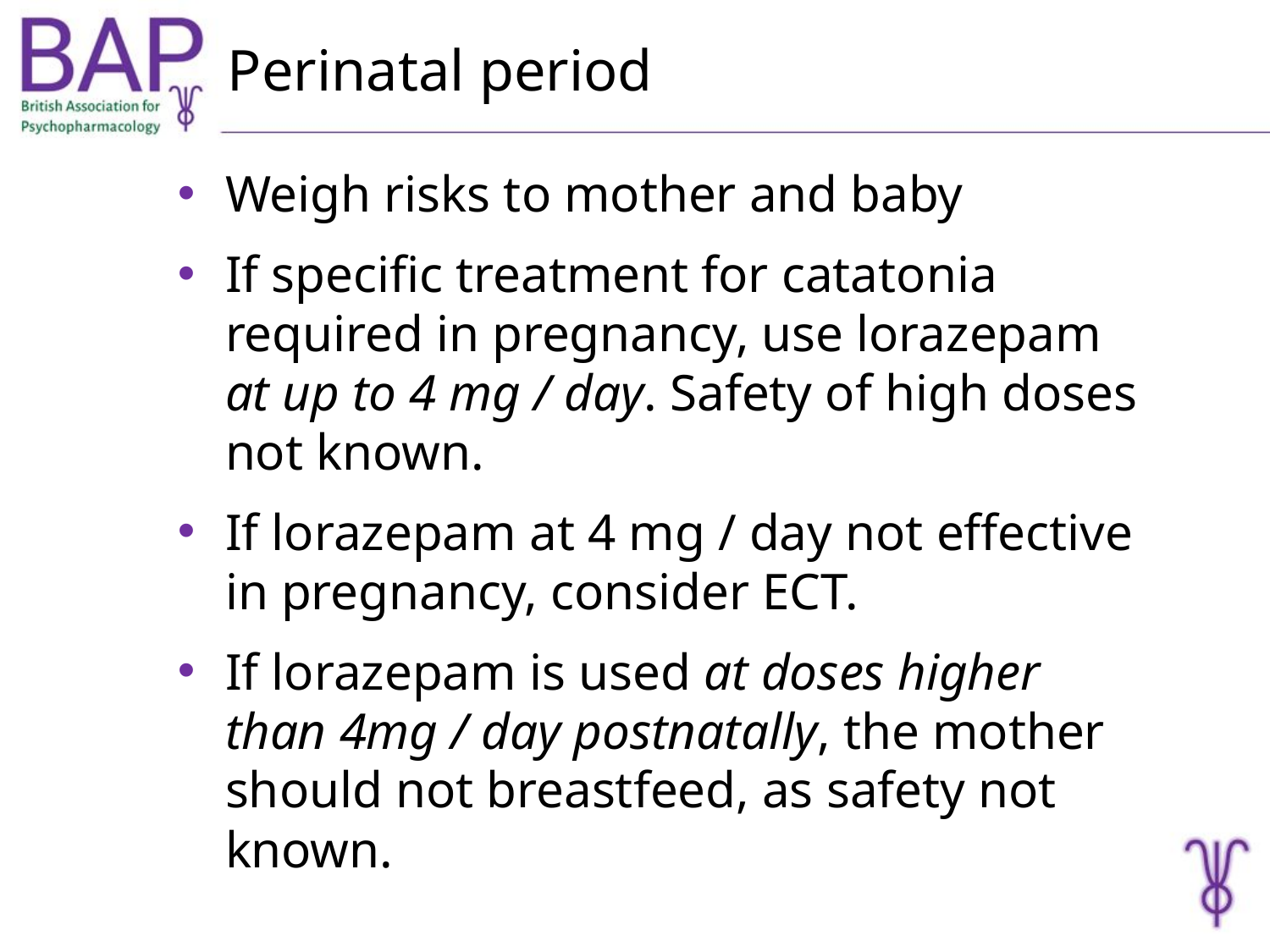

# Perinatal period
Weigh risks to mother and baby
If specific treatment for catatonia required in pregnancy, use lorazepam at up to 4 mg / day. Safety of high doses not known.
If lorazepam at 4 mg / day not effective in pregnancy, consider ECT.
If lorazepam is used at doses higher than 4mg / day postnatally, the mother should not breastfeed, as safety not known.

## Slide 30
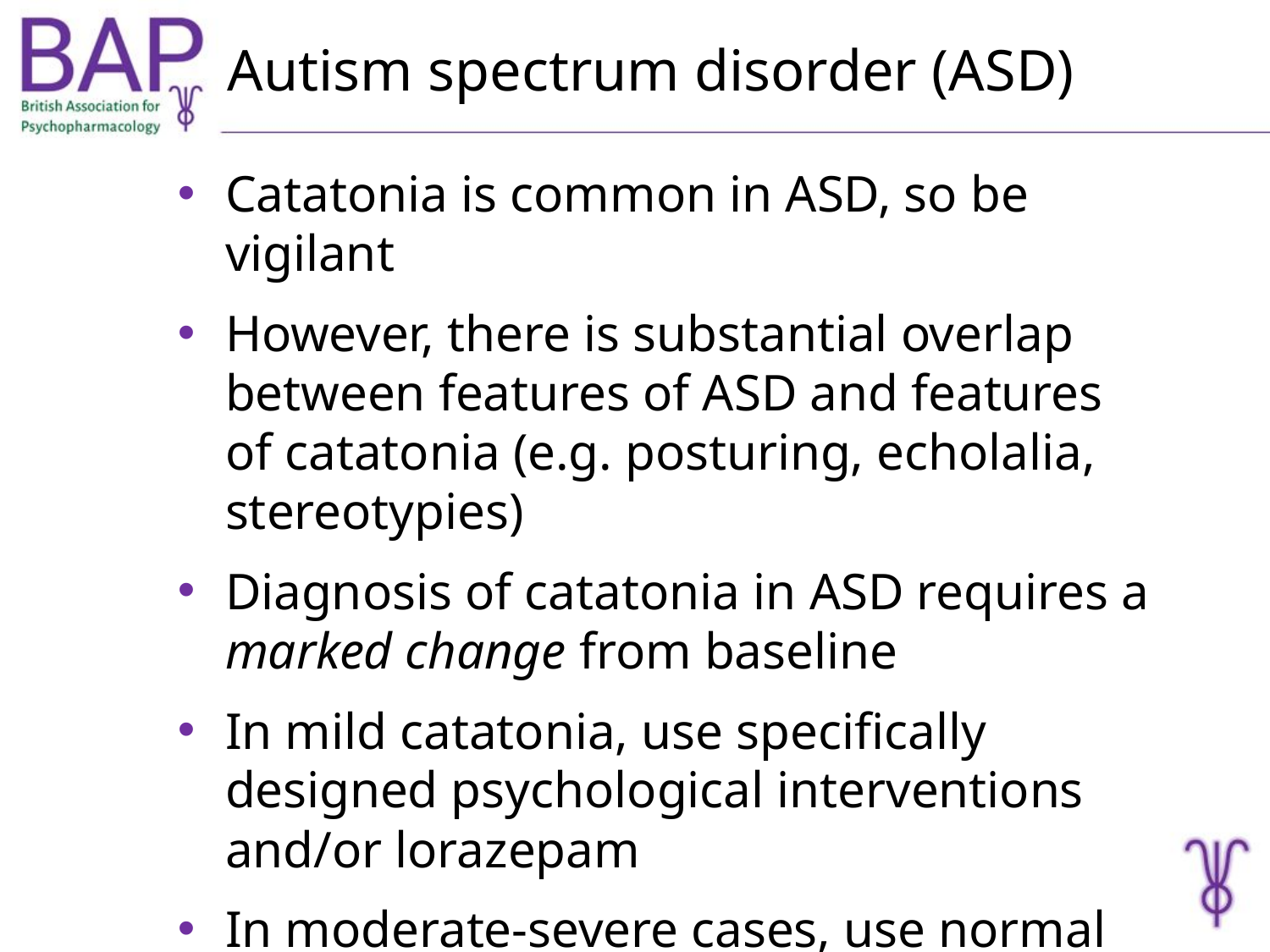

# Autism spectrum disorder (ASD)
Catatonia is common in ASD, so be vigilant
However, there is substantial overlap between features of ASD and features of catatonia (e.g. posturing, echolalia, stereotypies)
Diagnosis of catatonia in ASD requires a marked change from baseline
In mild catatonia, use specifically designed psychological interventions and/or lorazepam
In moderate-severe cases, use normal treatment algorithm

## Slide 31
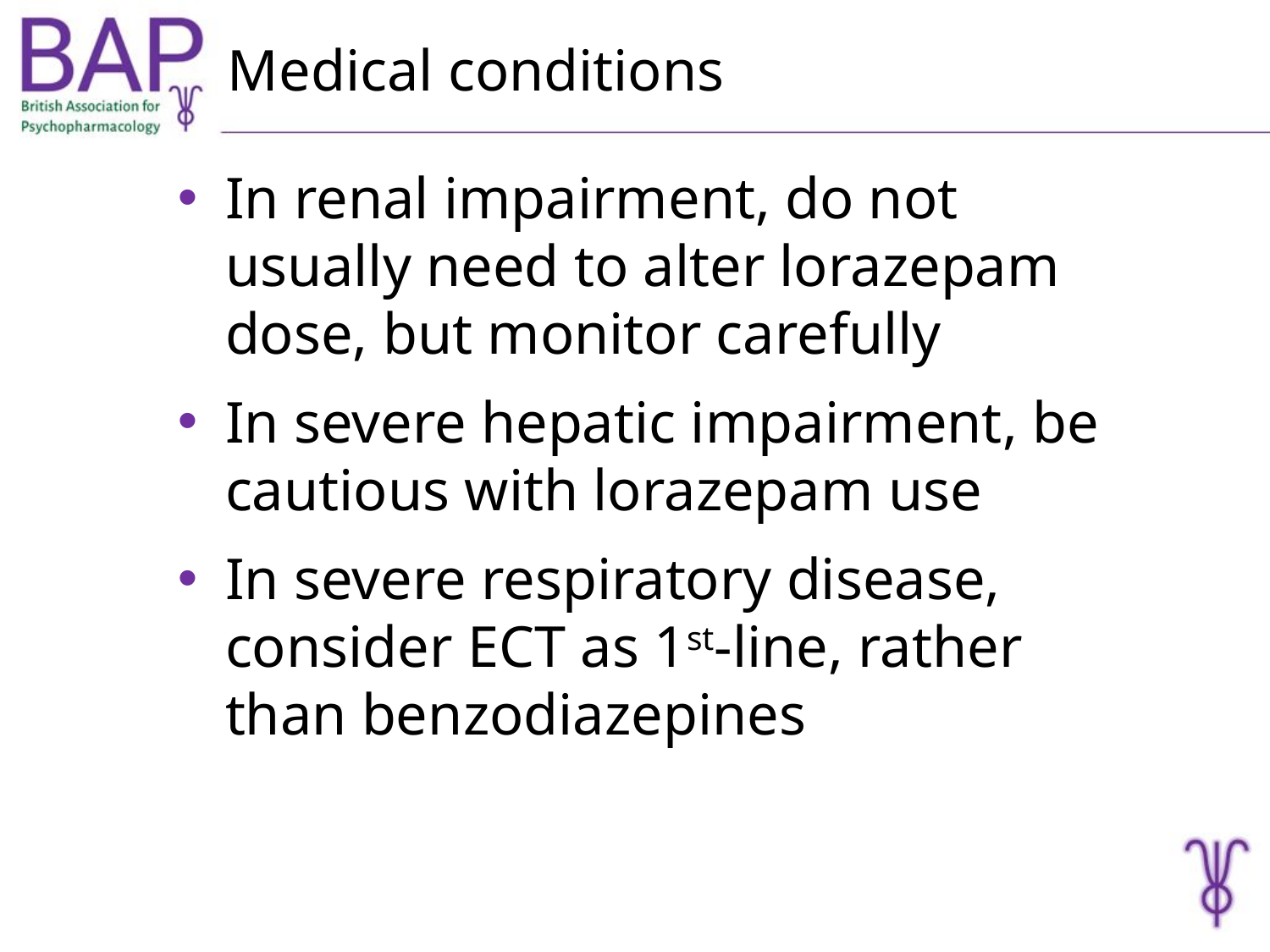

# Medical conditions
In renal impairment, do not usually need to alter lorazepam dose, but monitor carefully
In severe hepatic impairment, be cautious with lorazepam use
In severe respiratory disease, consider ECT as 1st-line, rather than benzodiazepines

## Slide 32
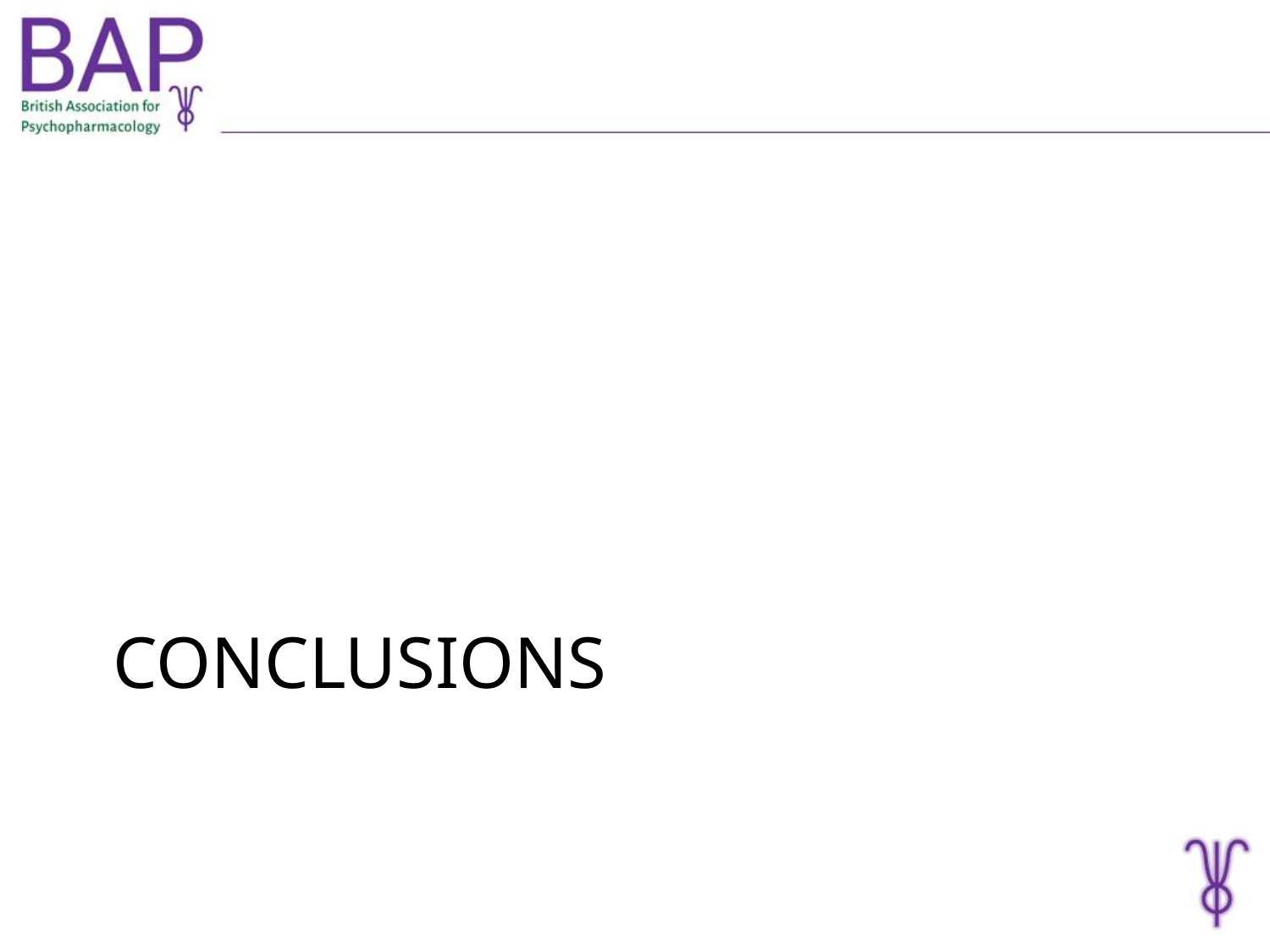

# Conclusions

## Slide 33
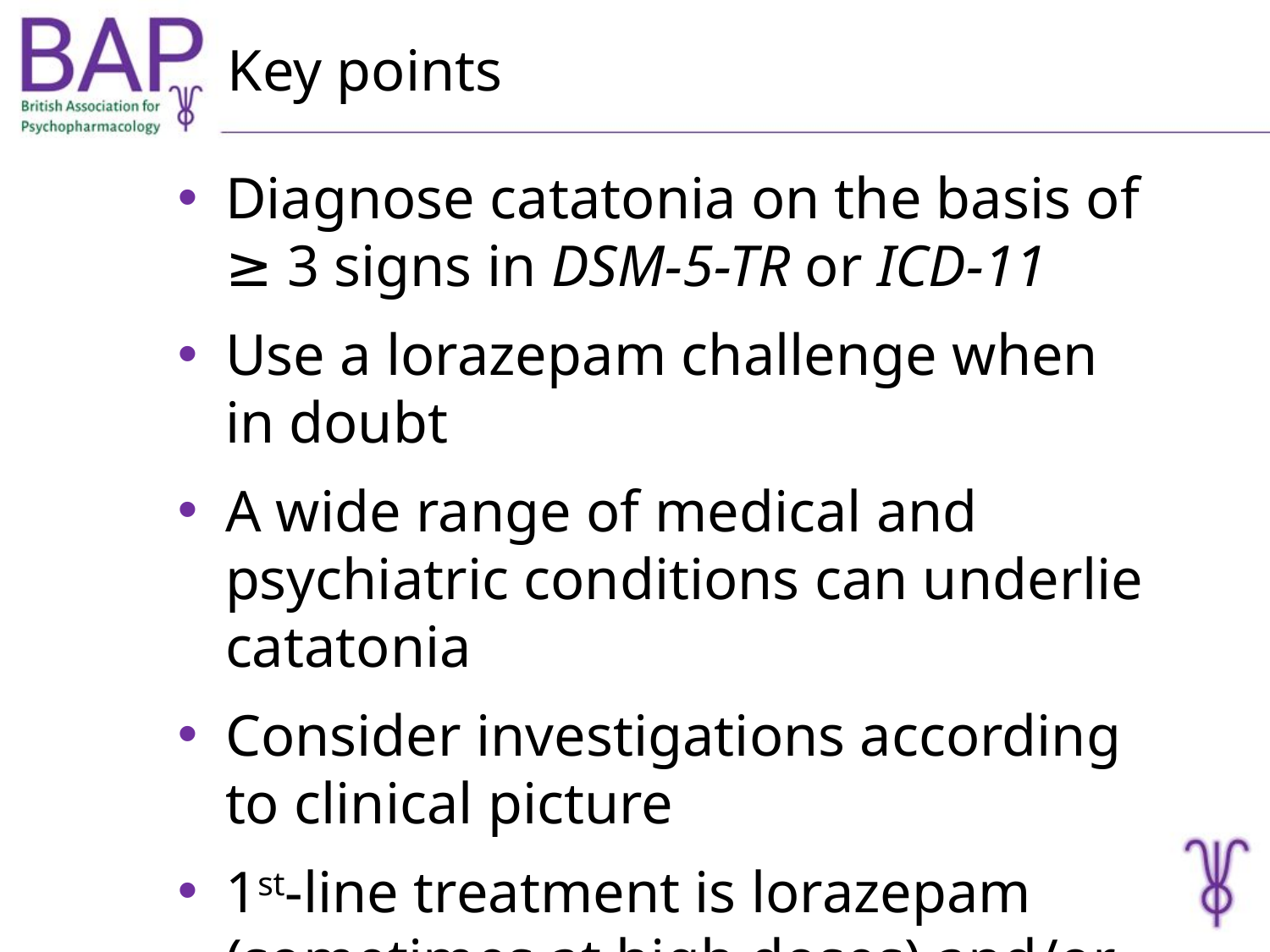

# Key points
Diagnose catatonia on the basis of ≥ 3 signs in DSM-5-TR or ICD-11
Use a lorazepam challenge when in doubt
A wide range of medical and psychiatric conditions can underlie catatonia
Consider investigations according to clinical picture
1st-line treatment is lorazepam (sometimes at high doses) and/or ECT

## Slide 34
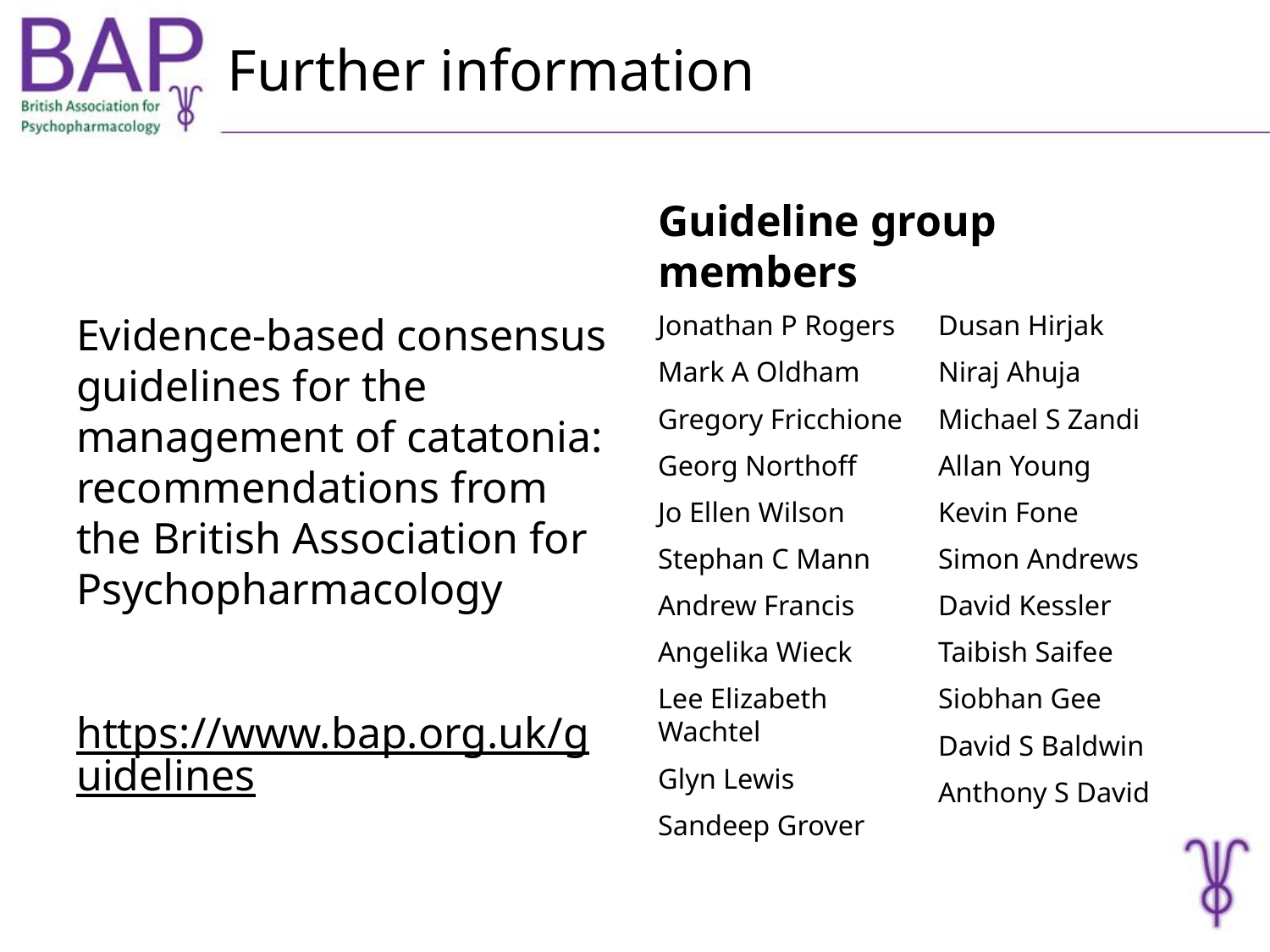

# Further information
Guideline group members
Evidence-based consensus guidelines for the management of catatonia: recommendations from the British Association for Psychopharmacology
https://www.bap.org.uk/guidelines
Jonathan P Rogers
Mark A Oldham
Gregory Fricchione
Georg Northoff
Jo Ellen Wilson
Stephan C Mann
Andrew Francis
Angelika Wieck
Lee Elizabeth Wachtel
Glyn Lewis
Sandeep Grover
Dusan Hirjak
Niraj Ahuja
Michael S Zandi
Allan Young
Kevin Fone
Simon Andrews
David Kessler
Taibish Saifee
Siobhan Gee
David S Baldwin
Anthony S David
